# Imaging brain activity during complex social behaviors in *Drosophila* with Flyception2

**DOI:** 10.1038/s41467-020-14487-7

**Published:** 2020-01-30

**Authors:** Dhruv Grover, Takeo Katsuki, Jinfang Li, Thomas J. Dawkins, Ralph J. Greenspan

**Affiliations:** 1 Kavli Institute for Brain and Mind, University of California, San Diego, La Jolla, CA USA; 2 Department of Electrical and Computer Engineering, University of California, San Diego, La Jolla, CA USA; 3 Division of Biological Sciences, University of California, San Diego, La Jolla, CA USA; 4 Department of Cognitive Science, University of California, San Diego, La Jolla, CA USA

**Keywords:** Fluorescence imaging, Optical imaging, Sensory processing, Social behaviour

## Abstract

Optical in vivo recordings from freely walking *Drosophila* are currently possible only for limited behaviors. Here, we expand the range of accessible behaviors with a retroreflective marker-based tracking and ratiometric brain imaging system, permitting brain activity imaging even in copulating male flies. We discover that P1 neurons, active during courtship, are inactive during copulation, whereas GABAergic mAL neurons remain active during copulation, suggesting a countervailing role of mAL in opposing P1 activity during mating.

## Abstract (web summary)

In vivo recordings of free-moving *Drosophila* neural activity are limited to clearly-separated flies in a stable focal plane. Here the authors improved on their Flyception imaging system to remove these constraints, and image neural activity in the male fly brain during various stages of the mating sequence.

## Introduction

Linking neural activity to complex behavior represents major ongoing work, not just in flies, but in most model organisms^1^. With the advent of genetically encoded calcium sensors, monitoring neural activity in vivo has been made possible with optical imaging techniques^2,3^. However, technologies that allow us to observe traces of neural activity across the spectrum of behaviors that animals can execute are rather limited.

Prior to this work, we developed an imaging paradigm to monitor neural activity in freely walking flies^4^. The method involved creating a chronic transparent window on the fly’s head to gain optical access to the brain, while allowing the animal to behave without restraints. Brain activity recordings were then performed by stabilizing this free-walking fly’s head under tracking and fluorescence camera sensors (1× magnification) by using two-axis galvanometer mirrors (at 1000 Hz). We demonstrated with this system, named Flyception, imaging of calcium transients in a male fly’s brain while he voluntarily engaged in courtship of a female.

Although this technique sheds light on the neural representation of naturalistic behaviors that had otherwise remained inaccessible under conventional imaging preparations, it was not without its limitations. For instance, the tracking algorithm relied on detecting the contour-based outline of the fly’s head. In situations when flies would make contact, this approach would merge their outlines, leading to a loss of tracking. Also, the cameras were focused on a fixed plane, and any behavior that caused the fly’s head to deviate from that focal range was not captured. As such, it was not possible to study brain activity in flies at all stages of the mating sequence, especially during copulation, when flies are in physical contact with each other, and the male, mounted on the female, moves out of the camera's focal range. Here, we describe Flyception2, a system that features three major enhancements to expand the behavioral repertoire accessible under the untethered imaging paradigm.

## Results

### Flyception2 system design

**Fig. 1:**
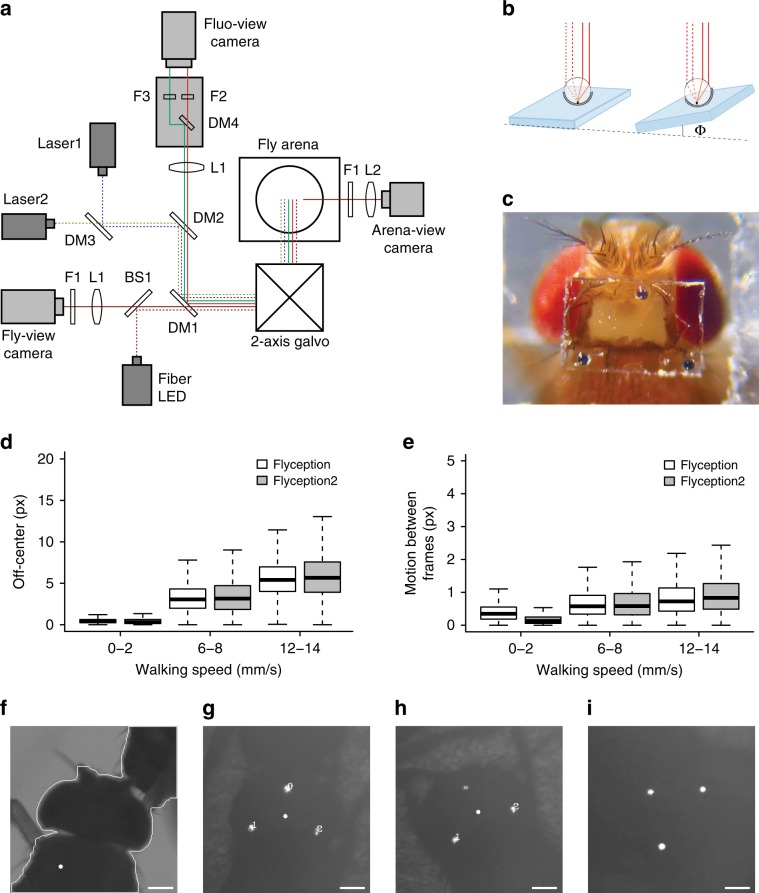
Retroreflector tracking and ratiometric fluorescence imaging of freely walking flies. **a** Schematic diagram of the tracking and fluorescence-imaging system. DM1, DM2, DM3, and DM4, dichroic mirrors; F1, F2, and F3, optical filters; L1 and L2, lenses. Solid lines shown in red, green, and brown indicate light paths for the red, green fluorescence, and arena-infrared backlighting, respectively. Dotted blue, yellow, and brown lines indicate light paths for the blue (473 nm), green–yellow (561 nm) laser illumination, and infrared (850 nm) overhead lighting, respectively. Galvo, galvanometer mirror. See Supplementary Fig. 1 for details. **b** Design and placement of the retroreflective marker on the coverslip used in this study. Dotted lines indicate incoming infrared light, and solid lines indicate the retroreflected light path back to the tracking camera. The right panel illustrates intact retroreflection despite tilting of the coverslip up to ~30°. **c** A view of the fly head with a surgically made optically clear brain-imaging window, and a retroreflective marker-based coverslip with three markers placed in a triangular pattern. See Supplementary Videos 1 and 2 for coverslip preparation and surgery protocol. **d** Comparison of head center position differences relative to the center of each fly-view image frame for different walking speeds under Flyception and Flyception2 tracking. px pixels. **e** Comparison of positional changes of the fly head between two consecutive frames in fly view for different walking speeds under Flyception and Flyception2 tracking. Boxplot whiskers are 1.5× interquartile range. **f** Flyception fly outline-based tracker merging contours of two flies due to a physical contact event. The white dot represents misidentification of focal fly head center position. **g** Flyception2 retroreflective marker-based tracking robustly tracking focal fly head center position during physical contact with another fly. **h** Tracking algorithm estimates the position of a single marker occluded from the tracking camera view, represented by a cross, to maintain the accurate head center position. **i** Example of intact retroreflection from a coverslip titled due to the male fly engaged in copulation.

First, we sought to improve the robustness of the tracking algorithm, especially when flies made physical contact with each other. To do so, we employed a method that tracks a retroreflective marker placed on the coverslip over the fly’s head^5^. The marker consisted of three 30-μm-diameter glass beads, hemispherically coated with aluminum, and attached along the edges of the imaging window in a triangular pattern (Fig. 1a–c, and Supplementary Movies 1 and 2). The retroreflective marker reflected the collimated infrared light directed at the fly through the galvanometer mirror system (positioned above the fly arena) and back up to the tracking camera that views the arena through the same mirror system (Supplementary Figs. 1 and 2). The tracking algorithm detected the illuminated markers as three bright spots on an otherwise dark background, the centroid of which was used as an estimate of the center of the fly’s head to update the angle of the galvanometer mirrors (Fig. 1g).

There are five principal advantages to this marker-based tracking approach over the previously employed contour-based tracking. First, it is computationally less expensive; tracking continues to function at a 1000-Hz update rate and potentially even beyond (Fig. 1d, e). Second, it requires a smaller field of view to initiate tracking, allowing for higher magnification or faster frame-rate tracking. Third, because the algorithm does not rely on the fly’s outline (Fig. 1f), it robustly tracks the head even in situations when the fly makes physical contact with other flies (e.g., during male courtship of a female fly) (Fig. 1g, h, and Supplementary Movie 3). Fourth, the retroreflector sends the incident light back to the source, regardless of the angle of incidence (for up to about 30°), making it possible to image the brain even when the fly tilts its head (Fig. 1i, Supplementary Fig. 8). Fifth, retroreflection is highly energy efficient compared with other light-emitting methods (e.g., fluorescence), and is suitable for high-frame-rate, short-exposure applications.

The second feature we developed is a system for fast-synchronous electronic control of the tracking and fluorescence camera lenses that permits real-time electronic adjustment of the lens’ focal planes. This allows manual tracking of the fly at different *z* planes over a range of several millimeters at variable step intervals (set to 100-μm steps for our experiments), as well as continuous tracking of the retroreflective marker along the *z* axis (i.e., autofocus). We implemented the command set used by a camera body to communicate with and control a detachable electronic focus lens (Supplementary Fig. 3), giving the user control of the ultrasonic motors in the lens’ assembly for manual or automatic bidirectional focal plane adjustment. This form of focus control offers a cost-effective solution with the precision, repeatability, synchrony, and speed needed to monitor brain activity during behaviors that involve height changes (Supplementary Figs. 4–6 and Supplementary Movie 4).

The third enhancement consists of a dual-excitation laser light path (473 and 561 nm) and emission light splitting optics that enable ratiometric imaging in the fly brain. The 473-nm laser was used to image calcium-dependent GCaMP (green), while the 561-nm laser was used for imaging calcium-independent tdTomato (red) fluorescence simultaneously in the same neuronal population at 50 Hz with a single sCMOS camera. This has made activity measurements more robust against motion artifacts, and allowed for comparison of neural activity among different behavioral states (Supplementary Fig. 7, and Supplementary Movies 5 and 6) and postures (Supplementary Fig. 8). The calcium-independent channel also allows for segmentation of the neurons without a priori information about their position and morphology. The system can, depending on use case, be easily adapted to image fluorescence markers of other wavelengths by configuring it with the appropriate excitation lasers and optical components (Supplementary Fig. 1).

Although our galvo-based tracking system robustly kept track of the retroreflective marker, not all video frames recorded were useful for quantification of GCaMP6s fluorescence signals; some frames are too blurry because the fly moved too fast, or the head was occluded by some body parts (often wings or legs) of the interacting fly, or the head is tilted beyond the range of the angle the retroreflective marker works. To ensure the quality of data analyzed and reproducibility of the analysis, we programmatically filtered frames to be used for dF/F calculation based on the following criteria: presence of three beads in the view, blurriness of the image (i.e., sharpness of the edge calculated by the Laplacian of Gaussian filter), and motion between frames (i.e., accuracy of tracking). After filtering, on average, about 75% of frames per 100-s recordings were found to pass the filtering, ensuring that the analysis would not include artifacts from out-of-focus images and nontrackable motion (Supplementary Fig. 15).

### Calcium imaging of olfactory projection neurons

To test our system’s ability for ratiometric imaging of calcium transients in the brain of free-walking flies, we presented odors to flies expressing GCaMP6s and tdTomato in olfactory projection neurons (*GH146-Gal4*), a region of the fly brain known to respond to odors^6^. When a 2-s pulse of ethanol odorant was presented to free-walking flies, we observed an increase in activity of these projection neurons in response to the odor (Supplementary Fig. 9a–d, and Supplementary Movie 7), largely consistent with previous findings under both tethered and free regimes^4,7^. Given that the flies were freely moving in the arena during the experiment, by analyzing their trajectories, we found that odorant application triggered flies to walk, and that after repeated presentations of the odor, flies tended to spend more time on the side of the arena where the odor port was located (Supplementary Fig. 9e, f). From a technical standpoint, activity changes were not affected by different walking speeds of the fly (Supplementary Fig. 9g), demonstrating the system’s fidelity in measuring brain activity in free-behaving animals. Furthermore, these forms of analyses and results also serve as examples of how the behavioral metrics that Flyception2 produces, such as animals’ trajectories and their higher-order moments, can be used to correlate stimulus, neural activity, and behavior.

### Calcium imaging of P1 neurons during courtship and mating

**Fig. 2:**
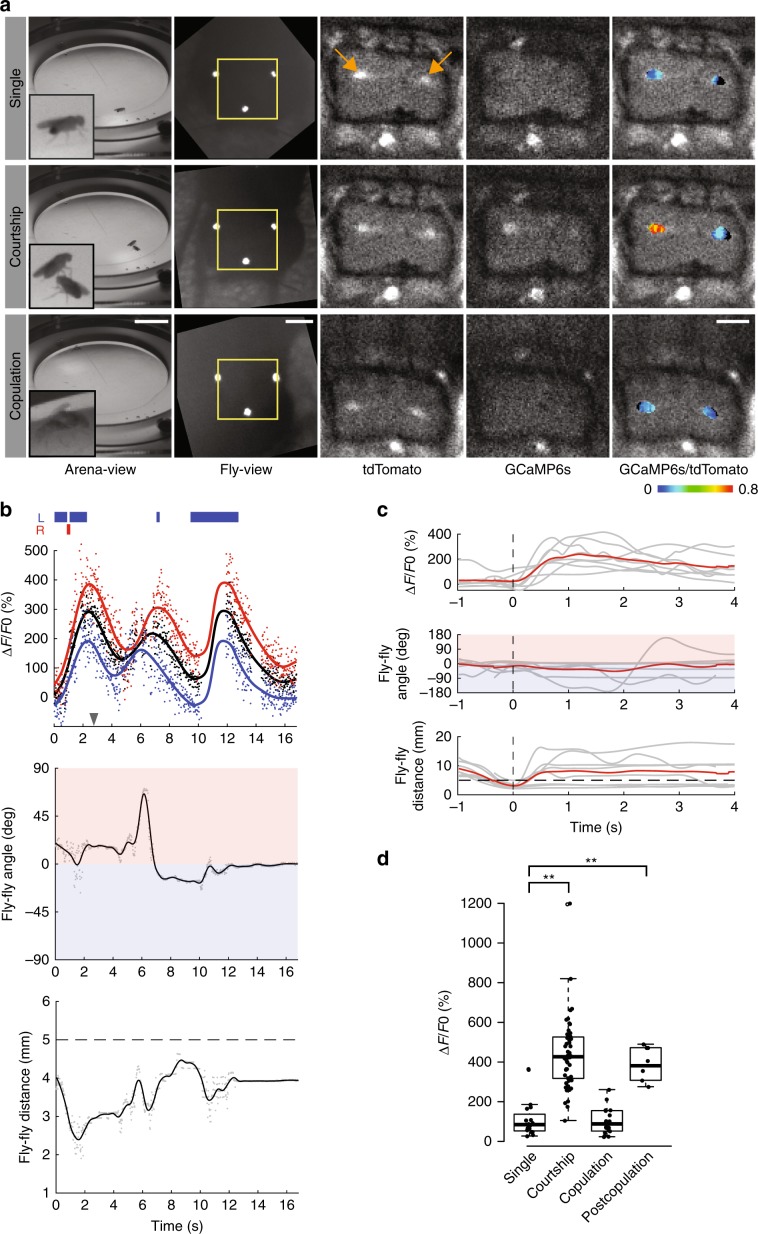
Activity of fru-expressing P1 neurons during naturally evoked courtship and copulation. **a** Top, a virgin male fly with *P1a-Gal4*, *UAS-GCaMP6s*, and *UAS-myr-tdTomato* transgenes. Middle, the same male fly courting a female fly. Bottom, the male fly copulating with the female fly. From left to right; arena-view, fly-view, fluo-view tdTomato channel, fluo-view GCaMP6s channel, and fluo-view tdTomato channel with a pseudocolor *F*_ratio_ = *F*_GCaMP6s_/*F*_tdTomato_ representation of the GCaMP6s signal. Yellow boxes indicate the areas shown in the fluo-view. Orange arrows indicate P1 neurons. **b** Top, *dF*_ratio_/*F*_ratio_ raw values for regions of interest encompassing both hemispheres of P1 neurons (black scatter), left hemisphere (blue scatter), and right hemisphere (red scatter), with corresponding LOESS (local polynomial regression) curve fits. Blue and red boxes indicate an extension of the left-wing and right-wing, respectively. The gray arrowhead indicates the time point at which the images in (**a**) the middle row were captured. Middle, angular difference of male fly’s heading relative to female fly. A positive (red region) or a negative (blue region) angular difference indicates the female fly is on the right-hand or left-hand side of the male, respectively. Bottom, euclidean distance between the flies. Dashed line at 5 mm indicates the upper distance threshold for interaction and contact events. **c** Top row, dF_ratio_/F_ratio_ values showing individual trial LOESS curve fits (gray), with mean curve (red) (*n* = 8). Middle row, angular difference (gray), with mean curve (red). Bottom row, euclidean distance between the male and female flies (gray), with mean curve (red). Traces in top and middle rows are time-locked to the bottom row, where flies are at their minimum distance less than 5 mm away from each other (dashed vertical line), with the additional criterion that prior to dropping below the distance threshold of 5 mm, they are at least 7 mm apart. **d** Activity of P1 neurons in naive males (*n* = 16), during courtship (*n* = 55), during copulation (*n* = 18), and during courtship after copulation (*n* = 6). **p < 0.001, Mann–Whitney *U* test. Scale bars, 10 mm for arena view, 200 μm for fly view, and 100 μm for fluo-view. Boxplot whiskers are 1.5× interquartile range.

With the aforementioned methods in place, we examined brain activity in male flies engaged in various stages of the mating sequence. We examined the activity patterns of a cluster of ~20 male-specific dorsal posterior *fruitless* gene-expressing neurons, namely the P1 interneurons, which are known to respond to female contact^8–10^. We used P1a split-Gal4 to express GCaMP6s and tdTomato specifically in the P1 interneurons and performed ratiometric imaging of these neurons in freely walking male flies (Fig. 2a, and Supplementary Movie 8). Consistent with previous results, we observed increased activity in P1 neurons as the male exhibited courtship behavior, approached, and contacted the female (Fig. 2a, b, and Supplementary Movie 9). Largely because of the marker-based tracking, we were able to observe complete interaction sequences of the two flies reliably, even when one fly’s head made direct contact with the other fly. By aligning multiple profiles of P1 neurons time-locked to the point when the two flies come within a minimum distance of 5 mm, we were able to reveal that increased activity of P1 neurons was preceded by a decreased distance between the male and female (Fig. 2c). In addition, by measuring fluorescence changes from each hemisphere, it was possible to capture sequences in which the activity of the P1 cluster on one side of the brain was stronger than that of the other side (Fig. 2a, b).

Little is known about neural dynamics in copulating flies and the roles of neurons implicated in courtship during this process, primarily because it has not been possible previously to visualize brain activity in flies engaged in mating with in vivo imaging techniques. The addition of electronic focusing adjustability of lenses in our setup allowed us to maintain focus on the male fly’s brain, as he mounted the female, and to observe the activity of P1 neurons during copulation in the same flies we used for recording courtship behavior. Strikingly, these neurons showed diminished activity during copulation as compared with the activity during courtship (*p* < 0.001, Mann–Whitney *U* test), despite physical contact with the female that drives activity in these neurons during courtship (Fig. 2a, d, and Supplementary Movie 10). Furthermore, we found that P1 activity returns post copulation when the male approaches and contacts the same female (Supplementary Fig. 10, and Supplementary Movie 11). These results suggest that the diminished GCaMP6s signal in P1 neurons during copulation is due to the fact that these neurons were modulated by the behavioral state of the animal.

### Calcium imaging of mAL GABAergic neurons during courtship and mating

**Fig. 3:**
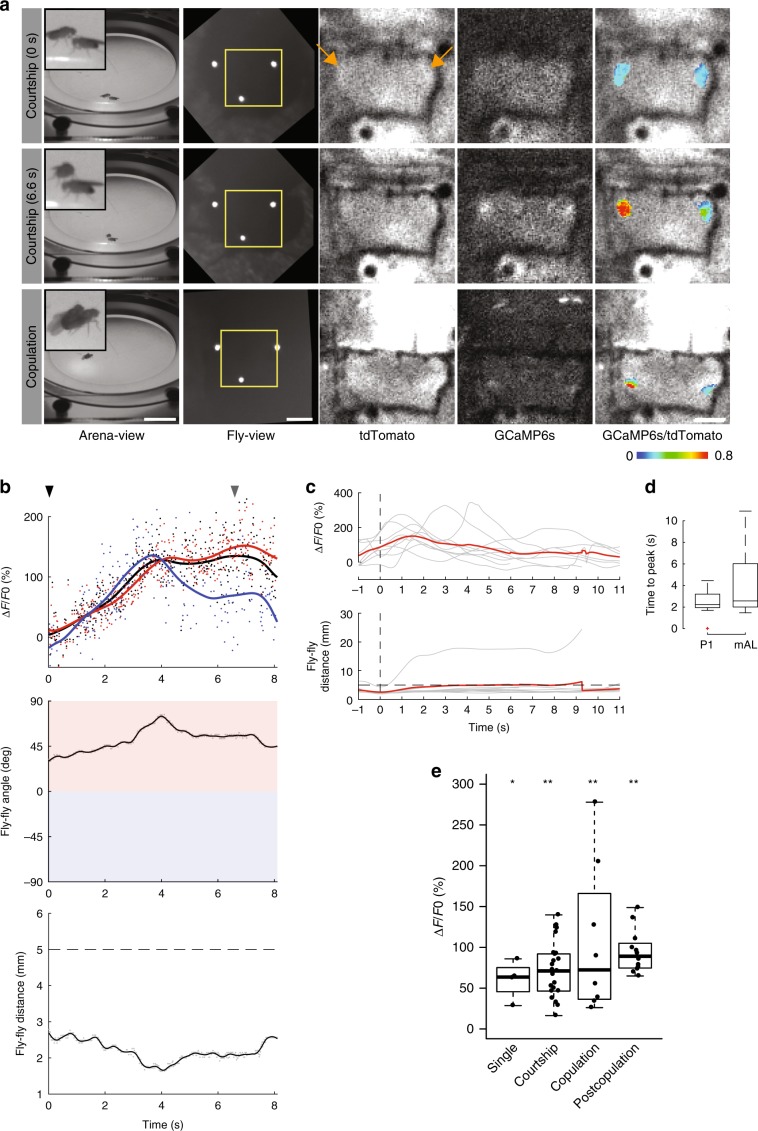
Activity of GABAergic mAL neurons during an interaction and copulation. **a** Top, a virgin male fly with *R25E04-Gal4*, *UAS-GCaMP6s*, and *UAS-myr-tdTomato* transgenes encountering a virgin female fly. Middle, the same male fly interacting with the female fly. Bottom, the male fly copulating with the female fly. From left to right; arena-view, fly-view, fluo-view tdTomato channel, fluo-view GCaMP6s channel, and fluo-view tdTomato channel with a pseudocolor *F*_ratio_ = *F*_GCaMP6s_/*F*_tdTomato_ representation of the GCaMP6s signal in mAL neurons. Yellow boxes indicate the areas shown in the fluo-view. Orange arrows indicate mAL neurons. **b** Top, *dF*_ratio_/*F*_ratio_ raw values for regions of interest encompassing both hemispheres of mAL neurons (black scatter), left hemisphere (blue scatter), and right hemisphere (red scatter), with the corresponding LOESS (local polynomial regression) curve fits (black, blue, and red, respectively). The black and gray arrowheads indicate the time points at which the images in (**a**) top row and middle row were captured, respectively. Middle, angular difference of male fly’s heading relative to female fly (gray) with LOESS curve fit (black). A positive (red region) or a negative (blue region) angular difference indicates the female fly is on the right-hand or left-hand side of the male, respectively. Bottom, euclidean distance between the male and female flies. Dashed line at 5 mm indicates upper distance threshold for interaction and contact events. (**c**) Top, *dF*_ratio_/*F*_ratio_ values showing individual trial LOESS curve fits (gray), with mean curve (red) (*n* = 9). Bottom, euclidean distance between the male and female flies. Top traces are time-locked to the bottom row, where flies are at their minimum distance less than 5 mm away from each other. **d** Boxplot of comparison of time to peak activity between interacting P1 and mAL flies, calculated as the time interval from minimum distance to peak *dF*_ratio_/*F*_ratio_ activity. **e** Activity of mAL neurons in naive males (*n* = 4), during courtship (*n* = 23), during copulation (*n* = 8), and during interactions after copulation (*n* = 12). *p < 0.005, **p < 0.001, Mann–Whitney *U* test. Scale bars, 10 mm for arena view, 200 μm for fly view, and 100 μm for fluo-view. Boxplot whiskers are 1.5× interquartile range.

During courtship, excitation of P1 neurons has been shown to be opposed by inhibition by a set of sexually dimorphic GABAergic interneurons (~30 cells/hemisphere), namely the mAL neurons^10,11^. By acting as an antagonist, mAL neurons have been implicated in playing a role in balancing the relative excitation and inhibition onto P1 neurons to shape species-specific pheromone responses^12^. We therefore hypothesized that these mAL neurons might be active and involved in inhibition of P1 neurons during copulation, rationalizing our earlier observation of P1 inactivity during mating. We used a driver that labels a set of ~12 mAL neurons per hemisphere (*R25E04-Gal4*) and expressed both GCaMP6s and tdTomato in these neurons, to perform ratiometric brain imaging of male flies engaged in mating. In accordance with our hypothesis, we found these neurons to be consistently active during copulation (Fig. 3a, e, and Supplementary Movie 13), suggesting that their antagonistic role in modulating P1 neurons might extend to mating.

In addition, prior to copulation, when the male encountered the female fly, we observed activity in mAL neurons, both when the male showed no proclivity toward female courtship (Fig. 3 and Supplementary Movie 14), and when rejected by the female (Supplementary Movie 15). Furthermore, we observed a lowering of mAL-related activity before the male initiated an interaction and displayed courtship (Supplementary Movie 16). By aligning multiple profiles of mAL activity in a similar way to that of P1 (aligned in time by an interaction event, Fig. 2c), we observed that mAL activity peaks appeared to have a larger variation in timing than P1 (Fig. 3c, d). After copulation, mAL neurons again displayed activity when the male encountered the same female (Fig. 3e, Supplementary Fig. 11, and Supplementary Movie 17). Taken together, these observations highlight the intricacies of mAL involvement in male courtship and mating, a detailed characterization of which is possible with the richness of data that can be acquired by the Flyception2 system.

This study describes three technical innovations to a nascent technology for brain imaging in freely walking flies. To demonstrate the power of the system, we chose to study mating behavior that has remained an experimentally intractable (under in vivo imaging) yet ethologically critical paradigm, and to allow new aspects of behavioral physiology, not accessible previously, to be revealed by this technical innovation. In sum, the continued development of such methods is vital to shining a spotlight onto the neural instantiation of naturally occurring complex behavior.

## Methods

### Fly stocks

*P1a-Gal4* was a gift from K. Asahina (Salk Institute). *R25E04-Gal4* and *GH146-Gal4* were obtained from the Bloomington Drosophila Stock Center (#49125 and #30026, respectively). *20XUAS-GCaMP6s, UAS-myr-tdTomato* was a gift from A. Calhoun (Princeton). *UAS-CD8-GFP* and *UAS-myr-tdTomato* were gifts from W. Joiner (UCSD).

### Experimental protocol

Flies were reared at 25 °C on a standard cornmeal–molasses–agar medium. We used adult male flies (6–10 days old) for surgery and imaging experiments. Detailed genotypes are provided in Supplementary Table 1. The fly arena was thoroughly cleaned with 70% ethanol a day prior to the experiment. Prior to adding fresh water in the moat and loading flies, the arena was again wiped down with a moist wiper (Kimwipe). A single naive male fly (with a surgically created brain window) was first gently loaded into the arena without anesthesia. The fly was allowed to acclimatize to the arena in the dark for ~5 min before tracking and fluorescence recordings were initialized.

For odor pulse experiments, a single 2-s pulse of ethanol odorant was delivered to the fly arena during each recording. For courtship and mating experiments, after baseline recordings for the single male were completed (usually 1–2 recordings), a single virgin female fly was then added into the arena, and courtship recordings commenced.

### Preparing retroreflective bead marker coverslips

In our experiments, we UV-glued three barium titanate solid glass microspheres (BTGMS-4.15 ϕ5–22 μm, Cospheric) to a diced 0.05-mm-thick coverslip (No. 000, Matsunami) (diced to 400 × 600 μm) positioned over the fly’s head (Fig. 1c). The beads were manually placed on the coverslip such that they resembled the vertices of an isosceles triangle with its apex facing in the forward direction of the fly. The first bead was positioned centrally along one long edge of the coverslip to coincide with the intersection of the anterior and medial axes of the fly body. The second and third beads were positioned along the opposite long edge of the coverslip, coinciding with the posterior lateral edges of the fly’s head.

The following procedure was used to glue the beads to the diced coverslip windows. First, small amounts of UV glue (KOA-300, Poly-Lite) were placed at the three locations on the coverslip with an insect pin (Size 00, Fine Science Tools). Retroreflective beads, spread out on a glass slide, were then picked up individually with an insect pin using capillary action and then placed on the spots of glue on the coverslip. The orientation of the beads on the coverslip was critical for achieving good retroreflective properties. As the beads were hemispherically coated with aluminum, maximum retroreflection was achieved by placing the beads with the glass side up and the aluminum half-shell side toward the coverslip. We used an upright microscope under 4× magnification and with reflective illumination (white mounted LED and 50:50 reflectance:transmission ratio beamsplitter mirror in the cube, MCWHL5 and BSW11R, Thorlabs) to ensure that beads were in the correct orientation. Beads in the correct orientation would appear bright and circular (Supplementary Fig. 2a–d). A UV spot-curing system (LED-200, Electro-Lite) was then used to cure the UV glue for 60 s. This simple marker arrangement enabled robust tracking of the fly’s head at high frame rates, while simultaneously allowing for obstructionless access to the brain for in vivo optical recording.

An alternative procedure that uses a standard student stereomicroscope with side-lit fiber LED illumination (Supplementary Fig. 2e) is also a possibility. In this scenario, when side-lit, beads in the correct orientation appear dark when viewed through the eyepiece. On beads that are tilted, however, varying degrees of light might reflect off the outer aluminum coating up to the eyepiece and appear brighter. We demonstrate this concept in Supplementary Fig. 2f and Supplementary Movie 1.

### Fly brain window surgery

The fly brain window surgery procedure is largely based on our previous work^4^, except for the addition of a marker-based coverslip glued to the head (Fig. 1c, and Supplementary Movie 2). Briefly, the procedure involves immobilizing a fly to a surgical platform with tape on a temperature-controlled stage set to 4 °C. Without covering the head with saline, the cuticle was removed from the dorsal side of the head by hand dissection using fine forceps (Dumont #5SF, Fine Science Tools, or Tip size A, resharpened at Corte Instruments). Immediately after removing the cuticle, air sacs, and fat bodies, a small amount of two-component transparent silicon adhesive KWIK-SIL (World Precision Instruments) was applied to the opening with the tip of a forcep. A No. 000 coverslip with retroreflective bead markers, prepared in advance, was then attached to the head with KWIK-SIL to create a flat surface for better optical performance. We glued the head to the thorax with KWIK-SIL to minimize head motion. We then released the surgically manipulated flies from the mounting platform and allowed them to recover on standard fly food for at least 1 day. Flies that recovered typically showed walking speeds >10 mm s^−1^ and negative geotaxis, and they were used for imaging experiments.

In our hands, flies largely survived the surgery procedure and recovered within 1–2 days with success rates of 88% (43 out of 49 total flies) for *P1a»GCaMP6s-tdTomato* male flies, 93% (52 out of 56 total flies) for *mAL»GCaMP6s-tdTomato* male flies, and 100% (four out of four flies) for *GH146»GCaMP6s-tdTomato* male flies.

Of the flies that recovered successfully from the surgery, we performed mating experiments on 16 *P1a»GCaMP6s-tdTomato* and 24 *mAL»GCaMP6s-tdTomato* male flies. All experimental flies exhibited courtship behaviors (interactions with females, tapping, wing extension, and chasing). The copulation success rate for *P1a»GCaMP6s-tdTomato* male flies was 63% (10 out of 16 flies), and for *mAL»GCaMP6s-tdTomato* male flies it was 50% (12 out of 24 flies). We performed odor pulse experiments on all four *GH146»GCaMP6s-tdTomato* male flies.

### Fly arena

The fly behavioral arena consisted of a transparent acrylic chamber with a concave elliptical arena of dimensions 44 mm (major axis) × 40 mm (minor axis), and surrounded by a 10-mm-wide water-filled moat^4^. A custom AR-coated (500–850 nm) glass window (76 mm diameter, 0.7 mm thickness) was placed above the arena to contain the flies during an experiment. A two-axis galvanometer mirror system was mounted 68.17 mm above the arena, with the center of the secondary mirror vertically aligned with the arena center and with its major axis as well (GVS012, Thorlabs). The concave surface was designed to keep the fly’s head orthogonal in relation to the rotating mirror system throughout the arena. Because the primary and secondary galvanometer mirror centers were separated from each other by 15.174 mm, rotations of the two mirrors produced arcs of different curvatures, leading to the concave arena contour with an elliptical perimeter.

### Odor delivery

Olfactory stimulation experiments were performed using a custom-built odor delivery system that allowed presentation of either humidified air, or when triggered, a 2-s pulse of ethanol odorant into the fly behavioral arena. First, a three-way pneumatic valve manifold system (Automation Direct, BVM-3425 and BVS-32C1-12D) was constructed to direct inflowing dry air into one of two outlet ports. The outlet ports were connected to 10 ml glass vials filled with either 3–4 ml of ethanol odorant or water. Airflow was switched between the two ports using an Arduino (Arduino Uno) controlled by the tracking software. When triggered by the user, the flow was programmed to switch from humidified air to ethanol for 2 s and then automatically back to humidified air. Finally, airflow into the fly behavioral arena was regulated using a mass flow controller (Alicat Scientific, MC-2SLPM-D) at a rate of 200 ml min^−1^.

### Imaging equipment

The arena and fly-view tracking cameras used were the Flea3 USB3.0 (FL3-U3-13Y3M-C, Point Grey) and the Gazelle Camera Link (GZL-CL-22C5M-C, Point Grey), set to resolutions of 512 × 512 px at 50 Hz and 240 × 240 px at 1000 Hz, respectively. We used a full configuration camera link frame grabber (XCelera PX4, OR-X4C0-XPF00, Teledyne Dalsa) for image acquisition from the fly-view camera. The camera used for fluorescence imaging was a Prime sCMOS (Photometrics), set to a resolution of 2048 × 256 px at 50 Hz, and controlled by Micro-Manager^13^.

The arena-view camera was fitted with a fixed focal 8-mm c-mount lens (M0814-MP2 2/3, Computar). The fly-view and fluo-view cameras used Canon EF 180 mm f/3.5 L Macro USM lenses. Apertures of both macro lenses were set at f/4.0 to match the aperture of the intermediate optical components to prevent ghost images. The macro lenses were attached to the two cameras via EOS EF-mount to C-mount adapters (Fotodiox). A light-tight tube system was created between the lens mount and the fly-view camera sensor (SM1A9, SM1A10, SM1L03, Thorlabs) to include a red longpass (625 nm, 84746, Edmund Optics). Image-splitting optics (W-VIEW GEMINI, Hamamatsu) were placed in the light path of the fluo-view camera (between the lens and camera) for simultaneous image acquisition of dual-wavelength images to allow for ratiometric imaging. These included a 580 nm dichroic beamsplitter (FF580-FDi01-25×36, Semrock), and two bandpass filters for GCaMP (520/35 nm, FF01-520/35-25, Semrock) and tdTomato (607/36 nm, FF01-607/36-25, Semrock) emission wavelengths. The distance between the fluo-view camera lens mounting flange and image-splitting optics was set to roughly 76 mm (SM1A9, SM1A10, and SM1L20, Thorlabs), determined empirically to achieve an optimal balance among image magnification, light efficiency, and sharpness.

### *Z*-focus lens control

For integrating electronic focal plane adjustment into the system, we built the interface used for communication between the camera and lens body of a standard DSLR camera (Canon EOS). This allowed electronic control of the lens motor system of both the fly-view and fluo-view camera lenses for simultaneous electronic focus control. For the physical connection we modified the EOS EF-mount to C-mount adapters (Fotodiox) used to connect the lenses to the fly- and fluo-view cameras. Holes were drilled in the adapter, giving access to the interface pins on the mounting flanges of the lenses and wires were attached, creating a custom harness for each lens (Supplementary Fig. 3a, b). Both harnesses were connected to the Serial Peripheral Interface module of a standard Arduino Uno microcontroller, via a custom PCB shield, which was programmed to mimic the behavior of the camera body (Supplementary Fig. 3c). Command signals were then sent simultaneously to both lenses controlled by the tracking software via a custom interface between the NI DAQ and the Arduino’s input pins. The tracking software included the capability to move the focus of the lenses in both directions, in arbitrary step increments as well as full sweeps. For our experiments, we used 10-step increments per command, and lens focus position was adjusted by no more than 50 steps to compensate for the height differential in the male fly’s head position due to copulation. The ultrasonic motor lenses (USM) were chosen over standard lenses for reasons of focus repeatability and image stability (Supplementary Fig. 4).

Although the courtship and mating behavior we report here did not require autofocus, to further extend the applicability of our technology, we have implemented an autofocus mechanism to our system. The autofocus capability was achieved by continuously monitoring the variance of Laplacian of the fly-view image, and compensating for the drop of variance by electronically moving the lens focus. Because the variance of Laplacian does not tell the direction of out-of-focus, we move the lens focus in a positive or negative direction randomly. If a lens motion decreases the variance of Laplacian, that means that the lenses have moved in the wrong direction; in this case, we move the lenses two steps in the opposite direction. If, on the other hand, a lens motion increases the variance of Laplacian, that means that the lenses have moved in the correct direction; thus, we keep the lenses moving in the same direction, until the variance of Laplacian surpasses the previous baseline. We have tested different step sizes of the lens motion, sampling rate of the variance of Laplacian, and thresholds for detecting the drop of the variance of Laplacian, and found that a step size of 1, sampling rate of 50 Hz, and variance threshold of 5, resulted in a robust tracking of motion along the *z* axis (Supplementary Figs. 5 and 6, and Supplementary Movie 4).

### Tracking and fluorescence illumination

The arena was backlit with a custom-made diffuse 4 × 4 in, 850-nm infrared LED light (~1 mW cm^−2^). We used this diffused backlit illumination to track multiple flies using the arena-view camera.

We used an 850-nm collimated fiber-coupled LED (M850F2 and F240SMA-850, Thorlabs) as an incident light source for fly-view tracking. The fiber light was retroreflected off beads glued to the coverslip over the fly’s head back onto the fly-view camera.

We used 50-mW blue (473 nm) and yellow (561 nm) lasers (Gem, Laser Quantum) to activate GCaMP6s and tdTomato signals, respectively, in the brain. The lasers were set to average power densities of 4.85 mW cm^−2^ (blue) and 3.8–4.77 mW cm^−2^ (yellow) when measured at the arena surface, and at those power levels, they had no detectable photobleaching effect over ten consecutive 100-s recordings^4^ (Supplementary Fig. 13).

### Mirror alignment and light path

The center of the galvanometer’s secondary mirror (placed in a custom-machined holder) was vertically aligned with the center of the arena at a height of 68.17 mm, and in line with the major axis of the arena.

The fly-view camera was aligned with the center of the primary galvanometer mirror, and placed behind a far-red super-resolution long-pass dichroic beamsplitter (660 nm, Di03-R660-T1-25×36, Semrock). Also placed in the light path (between the far-red dichroic beamsplitter and camera) was a 10:90 (reflectance:transmission ratio) plate beamsplitter (BSN11R, Thorlabs). This plate beamsplitter was used to direct infrared collimated fiber light down to the arena through the galvanometer mirrors, as well as to allow retroreflected infrared light back up from the fly to the fly-view camera (Supplementary Fig. 1b, c).

The fluorescence-imaging camera was placed in an orthogonal position in relation to the fly-view camera and aligned with the reflected light path of the far-red long-pass dichroic filter. A second dual-band dichroic beamsplitter (488/561 nm, Di01-R488/561-25×36, Semrock) was placed in the fluo-view camera’s light path, permitting only the transmission of green GCaMP6s fluorescence and red tdTomato fluorescence to the fluo-view camera (Supplementary Fig. 1b, c).

The two lasers were placed in an orthogonal direction to the fluo-view camera, their beams merged with a combination of kinematic mirrors (M1, Newport) and a green long-pass dichroic beamsplitter (LM01-503-25, Semrock), and directed toward the reflected light path of the aforementioned dual-band dichroic beamsplitter. The laser beams were reflected off the two dichroic filters glued to the custom-machined galvanometer assembly holder, onto the two galvanometer mirrors, down to the arena and, consequently, to the fly’s head. We placed a 2× beam expander (BE02M/A, Thorlabs) in the laser beam path to increase the surface area of the laser spot, and provide consistent illumination of the fly (Supplementary Fig. 1a).

### Real-time tracking and mirror update

The system was initialized with tracked positions of the focal fly in the arena camera view. A custom nearest-neighbor-based algorithm was used to track the identity of multiple flies in the arena view at 50 Hz. A mapping of 2D image coordinates in the arena-view to real-world coordinates in the arena was precomputed offline by detecting the position of a laser beam spot on the arena surface for varied galvanometer mirror positions. This mapping was then used in real time to back-project 2D pixel centroids of the detected flies to real-world coordinates. The galvanometer mirrors were directed to the 2D tracked location of a user-defined fly in the arena. The arena-view tracker was used to provide a coarse approximation of the focal fly’s position in the arena, and initially guide the galvanometer mirrors to the approximate location of the fly’s head.

Once the focal fly’s head was in view of the fly-view camera running in parallel, control of the galvanometer mirror system switched to receive positional updates from the fly-view camera tracker at 1000 Hz. The positional deviation of the fly’s head from the center of the fly-view image was calculated in each frame, and the galvanometer mirrors were rotated to compensate for the deviation. We used a resolution test target (R1L3S10P, Thorlabs) to determine the scaling coefficient to convert fly-view pixels to rotation angles for the galvanometer mirrors (Supplementary Fig. 14c). The image analysis routines for both the arena- and the fly-view cameras were implemented in Microsoft Visual C++ using computer vision (OpenCV) and multithreaded programming (OpenMP) libraries.

The galvanometer analog position range was set from −10 to +10 V with an output scaling factor of 0.5 V deg^−1^. The 2D fly position in real-world coordinates was converted to voltages for each output channel and relayed to the galvanometer servo driver board using a 16-bit digital-to-analog converter (PCIE-6351, National Instruments). The PCIe DAQ offered low-latency voltage to mirror updates (~400 μs). The total latency from galvanometer response time to camera frame grabbing and fly position determination and tracking update were well within the fly-view camera frame rate of 1000 Hz.

### Fly-view head tracking

A retroreflective marker-based tracking approach was developed to robustly track the fly’s head position in the fly-view image frame. In our experiments, we glued three retroreflective beads (30 μm in diameter) to coverslip windows positioned over the fly’s head, that, when connected, resembled the shape of an isosceles triangle facing the forward direction of the fly (Fig. 1c). The tracking algorithm, however, was designed to be generic and therefore agnostic of bead number. Incident light from the collimated IR fiber was retroreflected back up to the fly-view camera, with the three beads (if oriented correctly) appearing as bright white circles in an otherwise dark camera image. A global intensity threshold followed by morphological operations for noise elimination were applied to the raw image acquired from the fly-view camera, resulting in a binary image of the beads subtracted from the background. The centroid of the three beads was then used as an estimate of the center of the fly’s head, and this position was used to update the galvanometer mirror positions (Fig. 1g). In situations when a single bead might be obstructed from view, its position was estimated using the location information of the remaining beads (Fig. 1h), thus maintaining accurate track of the fly’s head center (Supplementary Movie 3).

### Video-recording procedure and output

Recording of the three camera images (arena view, fly view, and fluo view) was manually triggered to start at roughly the same time. The exposure timing and frame rate of the arena-view, fly-view, and fluo-view image acquisition were controlled by external triggers generated using FPGA hardware-based clock timers on a MyRIO device (National Instruments). Furthermore, to generate a visually detectable cue to align the frames of the three cameras temporally, a flash (TT560, Neewer) was used. The MyRIO timer also triggered the firing of the flash, set to coincide with the trigger onset of the arena-view, fly-view, and fluo-view cameras (Supplementary Fig. 12). The arena-view camera was set to capture 512 × 512 px images at 50 Hz with a 3-ms exposure; the fly-view camera captured 240 × 240 px images at 1000 Hz with a 0.9-ms exposure; the fluo-view camera captured 2048 × 256 px images at 50 Hz with an 18-ms exposure. Each recording typically lasted 100 s. Arena-view and fly-view sequences were saved in an uncompressed video format known as fly movie format^14^, and fluo-view data were saved in a tiff format. Included in each recording are camera timestamp log files, an arena-view background image, and arena- and fly-view output trajectory files (see the Supplementary Software user manual for output trajectory file specifications).

### Spatial resolution

Because the numerical aperture (NA) of our optical system was limited by the primary mirror in the galvo assembly, 10 mm in diameter and located 83.3 mm away from the specimen (fly’s head), the NA of the current Flyception2 configuration was 0.06. This configuration set the lateral resolution limit of the system to 4.3 μm and the depth of field to 140 μm under green light (520 nm). Because the macro lens for the fluo-view camera was set to 1× magnification, the spatial resolution of the fluorescence image was limited by the 6.5-μm pixel size of the sCMOS camera. Considering the Nyquist frequency, the resolution limit of our fluorescence-imaging system was 13 μm, consistent with the image of a resolution test target captured by our system (Supplementary Fig. 14).

### Behavioral metrics acquisition

As described above, the arena-view camera provides a full view of the behavioral arena with a resolution of 512 × 512 px at 50 Hz. The system tracks each fly in real time, concurrently saving the uncompressed raw arena-view camera video and a trajectory file containing the real-time *xy*-positional data and pixel size of each fly in the arena. Close proximity or contact between flies in arena view could lead to merging of their detected contours and consequently an interchanging of identity. It is important to note that the identity switching in arena view has no effect on tracking the head of the focal fly in fly view. Nevertheless, to aid studies of social behavior that require accurate tracking of the behavior of interacting flies (and to better correlate those behaviors with imaging of brain activity), we provide a k-means clustering-based offline arena-view video tracker that can separate merged contours and maintain accurate tracking (Supplementary Movies 3, 4–17), even during copulation events^15^ (Supplementary Movies 10 and 13). As part of this offline tracking software, we also provide the user with the ability to manually label, on a frame-by-frame basis, specific behavioral events of interest. For example, in our study, we hand-labeled left- and right-wing extension events using this strategy (Fig. 2b). However, these raw videos are also amenable to characterization of stereotyped behaviors with unsupervised learning techniques^16^.

The fly-view camera, focused on the fly’s head with 1× optical magnification at 1000 Hz, provides an order-of-magnitude higher spatial and temporal resolution of the focal fly’s behavior (body/head angle, front leg movement, and tapping, contact events), as well as a partial view of the second interacting fly (in our study, the female) (Supplementary Movie 12). Along with the raw fly-view video, a trajectory file containing the head center position (in pixels and real-world coordinates) and corresponding galvanometer mirror angles is saved for each frame in the video. The heading angle of the focal fly is derived using the location of the beads on the retroreflective marker coverslip placed on the fly’s head. Given that the beads were manually placed on the coverslip so that they resembled the vertices of an isosceles triangle with its apex facing in the forward direction of the fly, the heading angle is determined by taking the difference between the base of the isosceles triangle and the horizontal line. Using the raw position and angle data, various higher-order moments of the subjects’ trajectories and behavior (e.g., distance between flies, velocity, fly–fly angle, and angle to the stimulus) are then derived and output with the analysis scripts.

Finally, all these data are synchronized with the fluo-view camera that provides simultaneous recordings of GCaMP and tdTomato fluorescence intensities (2048 × 256 pixels at 50 Hz) for ratiometric quantification of all labeled neurons, or with user-defined regions of interest.

### Ratiometric fluorescence quantification

In short, we measured fluorescence intensity by creating a mask of the labeled neurons for each fluo-view video frame in the calcium-independent (tdTomato) channel, and ratiometrically comparing the intensity values of the corresponding pixels from both GCaMP and tdTomato channels in that masked region (Supplementary Fig. 15a). We performed postacquisition image analyses using R (CRAN), with its image processing software packages EBImage^17^, dipr (https://github.com/tkatsuki/dipr), and Flyception2R (https://github.com/tkatsuki/Flyception2R).

As described above, the raw fluo-view video consisted of both GCaMP and tdTomato channels, simultaneously captured on a single sCMOS sensor using image-splitting optics. This raw video was first split and cropped to size for each of the two fluo-view channels. The split videos were then spatially aligned using a fast-normalized cross-correlation template-matching algorithm based on the frame in which the flash was detected. We then scaled the fly-view videos to match the fluo-view videos, after which the fly- and fluo-view videos were spatially aligned using the same template-matching algorithm.

Next, as described in detail below, we calculated transformation functions for registering the videos from each fly-view frame so that the fly is always facing the bottom of the image. A mask of the retroreflective beads was first created by applying an adaptive threshold to each frame of the fly-view video (Supplementary Fig. 15b, c). The angle of the marker, determined as the angle between the base of the isosceles triangle and the horizontal line, was used to compensate for rotation. Translational compensation was achieved by offsetting the centroid of the triangular marker from the center of the fly-view image (Supplementary Fig. 15d), resulting in near-stationary fly images throughout the video. The same rotation and translation functions were applied to the fluo-view videos (Supplementary Fig. 15e, f). Frames with large motion (motion_thresh = 10 in the Flyception2R analysis script) or blur (focus_thresh = 950) were omitted from further analysis. Image blurriness was quantified by applying a Gaussian filter followed by a Laplacian filter (LoG filter) to the tdTomato fluo-view video. Frames with less than three beads in the view were also removed from analysis.

A user-defined rectangular region(s) of interest (ROI) was then set to mask the tdTomato-labeled brain region (Supplementary Fig. 15e, g). A 3 × 3 median spatial filter was applied to each frame to smooth acquisition noise. A binary mask of labeled neurons was created by applying adaptive thresholding to each frame of the tdTomato channel (Supplementary Fig. 15h). We further refined the binary segmentation mask by applying a series of morphological operations. Erosion followed by dilation (morphological opening) was applied with a diamond structuring element of size 3, and holes in the segmented regions were appropriately filled. A second global thresholding removed bits in binary mask that corresponded to pixels less than the mean of user-specified per-frame quantile of pixel intensities within the ROI. This was done to remove relatively low-intensity pixels and further refine the segmentation mask. Small isolated regions in the mask were removed if that region’s area was below a user-specified threshold to remove any spuriously labeled pixels. Background fluorescence intensities were sampled from a ring of pixels surrounding the final segmented regions for each channel, and were subtracted from the segmented pixels. The GCaMP6s signal was measured as a ratio, after background subtraction, of green fluorescence (GCaMP6s) over red calcium-independent fluorescence (tdTomato): *F*_ratio_ = (*F*_green_ − *F*_green_background_)/(*F*_red_ − *F*_red_background_) within the masked regions and shown in a rainbow pseudocolor (Supplementary Fig. 15i–k).

We calculated GCaMP6s activity levels at different stages of mating behaviors by first fitting a LOESS curve (span = 0.2) to the raw-intensity time-series data, and then computing the difference of maximum *F*_ratio_ values from a baseline level: *dF*/*F* = (*F*_ratio_ − *F*_ratio_baseline_)/*F*_ratio_baseline_. For P1 experiments, we obtained the baseline level for each fly by taking the mean of the minimum smoothed *F*_ratio_ values of video sequences for the fly before introducing a female in the arena (i.e., naive male) (Fig. 2, Supplementary Fig. 10). For mAL experiments, because mAL neurons showed activity even in naive flies, we obtained the baseline level by taking the mean of the minimum smoothed *F*_ratio_ values for video sequences that showed a signal change during interaction events prior to copulation (Fig. 3, Supplementary Fig. 11). Comparisons of group means were performed using the Mann–Whitney *U* test.

### Reporting summary

Further information on research design is available in the Nature Research Reporting Summary linked to this article.

## Supplementary information

Supplementary Information

Description of Additional Supplementary Files

Supplementary Movie 1

Supplementary Movie 2

Supplementary Movie 3

Supplementary Movie 4

Supplementary Movie 5

Supplementary Movie 6

Supplementary Movie 7

Supplementary Movie 8

Supplementary Movie 9

Supplementary Movie 10

Supplementary Movie 11

Supplementary Movie 12

Supplementary Movie 13

Supplementary Movie 14

Supplementary Movie 15

Supplementary Movie 16

Supplementary Movie 17

Supplementary Software 1

Reporting Summary

## Data Availability

The data sets generated and/or analyzed during this study are available from the corresponding author on reasonable request.

## Code availability

The Flyception2 source code, user manual, and drawings for custom-made components are provided as Supplementary Software 1. The latest source code and user manual are also freely available for download from a public repository at https://github.com/dgrover/flyception2. Also included in the repository are various mechanical and electrical drawings for fabricating custom-made components. Analysis routines along with a sample dataset are available for download from https://github.com/tkatsuki/Flyception2R. For a full parts list, see Supplementary Table 2.
