# Supplementary material for: Imaging brain activity during complex social behaviors in Drosophila with Flyception2
Source: Nat Commun. 2020 Jan 30;11:623. doi: 10.1038/s41467-020-14487-7 (PMC6992788; doi:10.1038/s41467-020-14487-7)
Supplement: Supplementary file 2 — Description of Additional Supplementary Files [file 41467_2020_14487_MOESM2_ESM.pdf]

## Description of Additional Supplementary Files

**Supplementary Movie Legends.**

**Supplementary Movie 1. Preparing retroreflective bead marker coverslips.** Three 30 μm diameter glass beads hemi-spherically coated with aluminum (glass side up) are placed along the edges of a coverslip diced to 400x600 μm in a triangular pattern.

**Supplementary Movie 2. Surgical procedure to create chronic brain imaging window in freely behaving flies.** Transparent chronic brain imaging window is created on the fly's head followed by the placement of the retroreflective marker coverslip.

**Supplementary Movie 3. 1,000 Hz tracking of a male fly courting a female fly.** A male fly with an imaging window and a retroreflective marker being tracked at 1,000 Hz update rate while naturally courting and making contacts with a female fly. Tracking is maintained through occlusion of a bead (26 sec). Left, arena-view sequence recorded at 50 Hz, middle, 2D real-world coordinate representation of the male and female flies in arena (major axis - 44 mm, minor axis - 40 mm, black circle outline), right, fly-view sequence recorded at 1,000 Hz. In arena-view, blue dot represents tracked centroid of the male fly, and red dot represents tracked centroid of the female fly. In center real-word view, the blue line indicates the head direction of the male fly (blue), and the red line indicates the direction of the female fly (red) relative to the male fly. Frame rate of video is reduced to 1/2x speed (25 Hz).

**Supplementary Movie 4. Lens autofocus of moving retroreflective marker coverslip.** A retroreflective marker coverslip is manually moved up to 300 μm in both positive and negative z-directions which is compensated for, in real-time, by movement of the lens to automatically keep the resulting image in focus. From left to right, fly-view images of focused but moving three-bead based retroreflective marker coverslip, time series of lens step adjustment to maintain coverslip focus, and change in variance of Laplacian from baseline measure (recorded at initiation of autofocus) used to determine lens movement direction.

**Supplementary Movie 5. Activity of calcium-independent ratio-metric imaging of fru-expressing P1 neurons in a male fly isolated in the arena.** A virgin male fly with *P1a-Gal4*, *UAS-CD8-GFP*, and *UAS-myr-tdTomato* transgenes. From left to right; arena-view, fly-view, fluo-view tdTomato channel, fluo-view GFP channel, and fluo-view tdTomato channel with a pseudo-color $F_{ratio} = F_{GFP}/F_{tdTomato}$ representation of the ratio-metric signal in P1 neurons. In arena-view, blue dot represents tracked centroid of the male fly. Frames marked with the slashed-o symbol were excluded from fluorescence quantification based on image quality (see Methods). Frame rate of video is reduced to 1/2x speed (25 Hz).

**Supplementary Movie 6. Activity of calcium-independent ratio-metric imaging of fru-expressing P1 neurons during naturally evoked courtship.** A virgin male fly with *P1a-Gal4*,

*UAS-CD8-GFP*, and *UAS-myr-tdTomato* transgenes naturally courting a female fly. From left to right; arena-view, fly-view, fluo-view tdTomato channel, fluo-view GFP channel, and fluo-view tdTomato channel with a pseudo-color $F_{ratio} = F_{GFP}/F_{tdTomato}$ representation of the ratio-metric signal in P1 neurons. In arena-view, blue dot represents tracked centroid of the male fly, and red dot represents tracked centroid of the female fly. Frames marked with the slashed-o symbol were excluded from fluorescence quantification based on image quality (see Methods). Frame rate of video is reduced to 1/2x speed (25 Hz).

**Supplementary Movie 7. Activity of olfactory projection neurons in response to an ethanol odor pulse in a free walking fly.** A male fly with *GH146-Gal4*, *UAS-GCaMP6s*, and *UAS-myr-tdTomato* transgenes. From left to right; arena-view, fly-view, fluo-view tdTomato channel, fluo-view GCaMP6s channel, and fluo-view tdTomato channel with a pseudo-color $F_{ratio} = F_{GCaMP6s}/F_{tdTomato}$ representation of the GCaMP6s signal in *GH-146* olfactory projection neurons. In arena-view, blue dot represents tracked centroid of the male fly. Frames marked with a green dot indicate 2 s pulsing of ethanol odorant in the arena. Frames marked with the slashed-o symbol were excluded from fluorescence quantification based on image quality (see Methods). Frame rate of video is reduced to 1/2x speed (25 Hz).

**Supplementary Movie 8. Activity of fru-expressing P1 neurons in a male fly isolated in the arena.** A virgin male fly with *P1a-Gal4*, *UAS-GCaMP6s*, and *UAS-myr-tdTomato* transgenes. From left to right; arena-view, fly-view, fluo-view tdTomato channel, fluo-view GCaMP6s channel, and fluo-view tdTomato channel with a pseudo-color $F_{ratio} = F_{GCaMP6s}/F_{tdTomato}$ representation of the GCaMP6s signal in P1 neurons. In arena-view, blue dot represents tracked centroid of the male fly. Frames marked with the slashed-o symbol were excluded from fluorescence quantification based on image quality (see Methods). Frame rate of video is reduced to 1/2x speed (25 Hz).

**Supplementary Movie 9. Activity of fru-expressing P1 neurons during naturally evoked courtship.** A virgin male fly with *P1a-Gal4*, *UAS-GCaMP6s*, and *UAS-myr-tdTomato* transgenes courting a female fly. From left to right; arena-view, fly-view, fluo-view tdTomato channel, fluo-view GCaMP6s channel, and fluo-view tdTomato channel with a pseudo-color $F_{ratio} = F_{GCaMP6s}/F_{tdTomato}$ representation of the GCaMP6s signal in P1 neurons. In arena-view, blue dot represents tracked centroid of the male fly, and red dot represents tracked centroid of the female fly. Frames marked with the slashed-o symbol were excluded from fluorescence quantification based on image quality (see Methods). Frame rate of video is reduced to 1/2x speed (25 Hz).

**Supplementary Movie 10. Activity of fru-expressing P1 neurons in a copulating male fly.** A male fly with *P1a-Gal4*, *UAS-GCaMP6s*, and *UAS-myr-tdTomato* transgenes copulating with a female fly. From left to right; arena-view, fly-view, fluo-view tdTomato channel, fluo-view

GCaMP6s channel, and fluo-view tdTomato channel with a pseudo-color $F_{ratio} = F_{GCaMP6s}/F_{tdTomato}$ representation of the GCaMP6s signal in P1 neurons. In arena-view, blue dot represents tracked centroid of the male fly, and red dot represents tracked centroid of the female fly. Frames marked with the slashed-o symbol were excluded from fluorescence quantification based on image quality (see Methods). Frame rate of video is reduced to 1/2x speed (25 Hz).

**Supplementary Movie 11: Activity of fru-expressing P1 neurons during courtship after copulation.** A male fly with *P1a-Gal4*, *UAS-GCaMP6s*, and *UAS-myr-tdTomato* transgenes interacting with a female fly after successful mating with the same female. From left to right; arena-view, fly-view, fluo-view tdTomato channel, fluo-view GCaMP6s channel, and fluo-view tdTomato channel with a pseudo-color $F_{ratio} = F_{GCaMP6s}/F_{tdTomato}$ representation of the GCaMP6s signal in P1 neurons. In arena-view, blue dot represents tracked centroid of the male fly, and red dot represents tracked centroid of the female fly. Frames marked with the slashed-o symbol were excluded from fluorescence quantification based on image quality (see Methods). Frame rate of video is reduced to 1/2x speed (25 Hz).

**Supplementary Movie 12. An illustration of adaptability of Flyception2 for finer resolution recordings of male fly's behavior coupled with activity of fru-expressing P1 neurons during naturally evoked courtship.** A virgin male fly with *P1a-Gal4*, *UAS-GCaMP6s*, and *UAS-myr-tdTomato* transgenes courting a female fly. From left to right; arena-view, fly-view, fluo-view tdTomato channel, fluo-view GCaMP6s channel, and fluo-view tdTomato channel with a pseudo-color $F_{ratio} = F_{GCaMP6s}/F_{tdTomato}$ representation of the GCaMP6s signal in P1 neurons. Higher intensity infra-red illumination of the backlight placed under the fly behavior arena provides better visibility of the male fly's behavior in fly-view during interactions with the female and may be beneficial for certain studies. In arena-view, blue dot represents tracked centroid of the male fly, and red dot represents tracked centroid of the female fly. Frames marked with the slashed-o symbol were excluded from fluorescence quantification based on image quality (see Methods). Frame rate of video is reduced to 1/2x speed (25 Hz).

**Supplementary Movie 13. Activity of GABAergic mAL neurons in a copulating male fly.** A male fly with *R25E04-Gal4*, *UAS-GCaMP6s*, and *UAS-myr-tdTomato* transgenes copulating with a female fly. From left to right; arena-view, fly-view, fluo-view tdTomato channel, fluo-view GCaMP6s channel, and fluo-view tdTomato channel with a pseudo-color $F_{ratio} = F_{GCaMP6s}/F_{tdTomato}$ representation of the GCaMP6s signal in mAL neurons. In arena-view, blue dot represents tracked centroid of the male fly, and red dot represents tracked centroid of the female fly. Frames marked with the slashed-o symbol were excluded from fluorescence quantification based on image quality (see Methods). Frame rate of video is reduced to 1/2x speed (25 Hz).

**Supplementary Movie 14. Activity of GABAergic mAL neurons during an interaction.** A virgin male fly with *R25E04-Gal4*, *UAS-GCaMP6s*, and *UAS-myr-tdTomato* transgenes

interacting with a female fly. From left to right; arena-view, fly-view, fluo-view tdTomato channel, fluo-view GCaMP6s channel, and fluo-view tdTomato channel with a pseudo-color $F_{ratio} = F_{GCaMP6s}/F_{tdTomato}$ representation of the GCaMP6s signal in mAL neurons. In arena-view, blue dot represents tracked centroid of the male fly, and red dot represents tracked centroid of the female fly. Frames marked with the slashed-o symbol were excluded from fluorescence quantification based on image quality (see Methods). Frame rate of video is reduced to 1/2x speed (25 Hz).

**Supplementary Movie 15. Activity of GABAergic mAL neurons after an unsuccessful courtship attempt.** A virgin male fly with *R25E04-Gal4*, *UAS-GCaMP6s,* and *UAS-myr-tdTomato* transgenes during an unsuccessful courtship attempt of a female fly. From left to right; arena-view, fly-view, fluo-view tdTomato channel, fluo-view GCaMP6s channel, and fluo-view tdTomato channel with a pseudo-color $F_{ratio} = F_{GCaMP6s}/F_{tdTomato}$ representation of the GCaMP6s signal in mAL neurons. In arena-view, blue dot represents tracked centroid of the male fly, and red dot represents tracked centroid of the female fly. The male exhibits courtship (11 sec), after which we observe an increase in mAL activity (17 sec). Frames marked with the slashed-o symbol were excluded from fluorescence quantification based on image quality (see Methods). Frame rate of video is reduced to 1/2x speed (25 Hz).

**Supplementary Movie 16. Activity of GABAergic mAL neurons during a female initiated interaction followed by male courtship.** A virgin male fly with *R25E04-Gal4*, *UAS-GCaMP6s*, and *UAS-myr-tdTomato* transgenes approached by a female and subsequently attempts to court her. From left to right; arena-view, fly-view, fluo-view tdTomato channel, fluo-view GCaMP6s channel, and fluo-view tdTomato channel with a pseudo-color $F_{ratio} = F_{GCaMP6s}/F_{tdTomato}$ representation of the GCaMP6s signal in mAL neurons. In arena-view, blue dot represents tracked centroid of the male fly, and red dot represents tracked centroid of the female fly. mAL activity is observed during female initiated interactions (9 sec), which is followed by a lowering of activity and ultimately male courtship (33 sec). Frames marked with the slashed-o symbol were excluded from fluorescence quantification based on image quality (see Methods). Frame rate of video is reduced to 1/2x speed (25 Hz).

**Supplementary Movie 17. Activity of GABAergic mAL neurons during an interaction after copulation.** A male fly with *R25E04-Gal4*, *UAS-GCaMP6s*, and *UAS-myr-tdTomato* transgenes interacting with a female fly after successful mating with the same female. From left to right; arena-view, fly-view, fluo-view tdTomato channel, fluo-view GCaMP6s channel, and fluo-view tdTomato channel with a pseudo-color $F_{ratio} = F_{GCaMP6s}/F_{tdTomato}$ representation of the GCaMP6s signal in mAL neurons. In arena-view, blue dot represents tracked centroid of the male fly, and red dot represents tracked centroid of the female fly. Frames marked with the slashed-o symbol were excluded from fluorescence quantification based on image quality (see Methods). Frame rate of video is reduced to 1/2x speed (25 Hz).

**Supplementary Software Legend.**

**Supplementary Software 1. Flyception2 source code and system component schematics.**
