# Supplementary material for: Imaging brain activity during complex social behaviors in Drosophila with Flyception2
Source: Nat Commun. 2020 Jan 30;11:623. doi: 10.1038/s41467-020-14487-7 (PMC6992788; doi:10.1038/s41467-020-14487-7)
Supplement: Supplementary file 20 — Supplementary Software 1 [file 41467_2020_14487_MOESM20_ESM.zip › Supplementary Software 1/flyception2_tracking/myRIO/Flyception2_Trigger/documentation/myRIO Custom FPGA Project Documentation.html]

myRIO Custom FPGA Project


# myRIO Custom FPGA Project

You can use the **myRIO Custom FPGA Project** template as a starting point for creating myRIO applications with your custom FPGA code. With the **myRIO Custom FPGA Project** template, you can easily modify the code of the myRIO shipping FPGA personality.

This template is suitable for applications that have the following characteristics:

- Contain multiple time-critical elements that need to run in parallel.
- Require more precise timing than the myRIO RT controller can achieve.
- Use a type of I/O that is not available with the myRIO shipping FPGA personality.

The following example applications can benefit from the use of this template:

- A check engine auto scanner that supports the CAN protocol.
- An audio effects generator that analyzes an audio sample and simultaneously generates multiple effects.

## Table of Contents

System Requirements

Understanding the Components of This Template

Running This Template

Modifying This Template

Related Information

---


## System Requirements

### Development System

- LabVIEW Full or Professional Development System
- LabVIEW Real-Time Module
- LabVIEW FPGA Module
- Xilinx compilation tools for Vivado
- LabVIEW myRIO Toolkit

Use the LabVIEW FPGA Module to create custom FPGA code. Use the myRIO VIs and the LabVIEW Real-Time Module to create real-time (RT) code. The FPGA code and the RT code run in parallel.

### Hardware

- myRIO

## Understanding the Components of This Template

This template consists of the following VIs:

- **FPGA Main Default.vi/FPGA High Throughput.vi**—Contains the myRIO shipping FPGA personality. This VI compiles and runs on the myRIO FPGA target. You can modify this VI to suit your application needs. NI recommends that you only add new FPGA code to this VI and do not modify the existing code. This VI might not correctly work with the myRIO VIs if you modify the existing code.  
    
  **Note**  NI provides two types of myRIO FPGA personalities: default FPGA personality and high-throughput FPGA personality. By default, you can use the default FPGA personality with the myRIO Toolkit. Visit ni.com/info and enter the Info Code ex6g5a to learn more about how to use the high-throughput FPGA personality.
- **RT Main.vi**—Contains the main myRIO application. This VI performs the following tasks:
  - Downloads the code from the FPGA Main Default VI/FPGA High Throughput VI to the myRIO FPGA target and runs the code.
  - Sets the custom FPGA reference so that the myRIO VIs can work with the custom FPGA code.
  - Acquires and processes data from the user button on the myRIO and accesses any custom FPGA I/O.

The RT Main VI in this template uses a Flat Sequence structure that executes frames from left to right:

The Flat Sequence structure in this template executes the following frames from left to right:

1. **Initialize myRIO with custom FPGA**—Initializes the application. Use this frame to open and set the custom FPGA bitfile that the FPGA Main Default VI/FPGA High Throughput VI generates. You can also use this frame to initialize the myRIO, variables, and user interface elements, such as controls and indicators.
2. **Acquire and process data**—Acquires data from the myRIO and processes the data for presentation. The While Loop repeats code until you click **Stop** or an error occurs. You can use the myRIO VIs or the LabVIEW FPGA host interface in this frame. Use the myRIO VIs to access any myRIO default functionality. Use the FPGA host interface for any custom elements that you add to the FPGA Main Default VI/FPGA High Throughput VI.
3. **Close**—Closes and resets the custom FPGA code. You can also use this frame to close user interface elements and variables before the application exits.

## Running This Template

1. In the **Project Explorer** window, navigate to your myRIO FPGA target to find the FPGA Main Default VI/FPGA Main High Throughput VI.
2. Right-click the FPGA Main Default VI/FPGA Main High Throughput VI and select **Create Build Specification** from the shortcut menu. Verify that FPGA Main Default/FPGA Main High Throughput appears under **Build Specifications**.
3. Select **File»Save** to save the specification to the LabVIEW project.
4. Right-click FPGA Main Default/FPGA Main High Throughput under **Build Specifications** and select **Build** from the shortcut menu to display the **Select Compile Server** dialog box.
5. Click **OK** to generate the custom FPGA bitfile.
6. Open and run the RT Main VI.
7. Press the user button on the myRIO and observe the state change of the **Button Value** indicator.
8. Click **Stop** to stop the application.

## Modifying This Template

The following table summarizes the design decisions you must make when modifying this template.

| Design Decision | Design Example | Detailed Information |
| --- | --- | --- |
| How do you want to control the order of execution in your application? | You want data to flow as desired without using the Flat Sequence structure. | Controlling the Order of Execution |
| What data does your application need to initialize? | You want your application to open and set the custom FPGA bitfile. | Adding Initialization Code |
| Which myRIO I/O channels does your application need to access? | You want your application to control the DIO channels on the myRIO. | Modifying the Data Acquisition and Processing Code |
| What executions does your application need to complete before exiting? | You want your application to close the custom FPGA reference and reset the FPGA target. | Adding Closing Code |
| How does your application handle errors? | You want your application to report errors at the end of the execution flow. | Modifying Error Handling |

### Controlling the Order of Execution

This template uses the Flat Sequence structure to control data flow. The data leaves each frame as the frame finishes executing. This template uses the Flat Sequence structure to demonstrate the purpose of the code in each frame.

**Note**  Do not overuse the Flat Sequence structure for controlling the execution order because the Flat Sequence structure prohibits parallel operations.

When you modify this template, you can remove the Flat Sequence structure and establish data dependency between nodes. For example, wiring the **error in** and **error out** clusters through nodes controls the data to flow from one node to another.

### Adding Initialization Code

This template initializes the FPGA bitfile that the FPGA Main Default VI/FPGA Main High Throughput VI generates. If you want to use your custom FPGA personality, you must open and set the custom FPGA bitfile. Otherwise, the myRIO uses the shipping FPGA personality. You also can initialize the data to specific values or to open a file for logging.

### Modifying the Data Acquisition and Processing Code

This template uses the Button Express VI to acquire user button data from the myRIO and displays the button value on a Boolean indicator.

You can add code to acquire data from the myRIO and process the data based on your application needs. Use the myRIO VIs to access myRIO I/O channels and the onboard devices. Add time-critical functions to your FPGA code and use the FPGA host interface to access the custom elements that you add to the FPGA code. Adding time-critical functions to your FPGA code allows the FPGA functions and RT functions to run in parallel at a fast rate.

### Adding Closing Code

This template uses the Close FPGA VI Reference function to close the reference to the FPGA Main Default VI/FPGA Main High Throughput VI. You must close the FPGA reference to reset the FPGA target at the end of the application.

You can add code that executes after the data acquisition and processing completes but before the application exits. Closing code commonly accomplishes the following tasks:

- Frees memory by closing any open references
- Flushes any buffers in use
- Resets the myRIO

### Modifying Error Handling

By default, if an error occurs in the Button Express VI, this template stops. If you do not want the error to stop your application, you can remove the OR function and wire the **Stop** control directly to the conditional terminal of the While Loop. The **error in** and **error out** clusters pass error information from the beginning of the VI to the end and report the error at the end of the execution flow.

## Related Information

Refer to the *LabVIEW Help*, available by selecting **Help»LabVIEW Help** from LabVIEW, for information about LabVIEW concepts or objects used in this sample project. You also can use the **Context Help** window to learn basic information about LabVIEW objects as you move the cursor over each object. To display the **Context Help** window in LabVIEW, select **Help»Show Context Help**.

If you use the myRIO high-throughput FPGA personality, refer to the **Voice Recorder** sample project, available from the **Create Project** dialog box, for an example of adapting this template to an audio sampling application.

---

## Legal Information

**Copyright**

© 2013–2015 National Instruments. All rights reserved.

Under the copyright laws, this publication may not be reproduced or transmitted in any form, electronic or mechanical, including photocopying, recording, storing in an information retrieval system, or translating, in whole or in part, without the prior written consent of National Instruments Corporation.

National Instruments respects the intellectual property of others, and we ask our users to do the same. NI software is protected by copyright and other intellectual property laws. Where NI software may be used to reproduce software or other materials belonging to others, you may use NI software only to reproduce materials that you may reproduce in accordance with the terms of any applicable license or other legal restriction.

**End-User License Agreements and Third-Party Legal Notices**

You can find end-user license agreements (EULAs) and third-party legal notices in the following locations after installation:

- Notices are located in the <National Instruments>\\_Legal Information and <National Instruments> directories.
- EULAs are located in the <National Instruments>\Shared\MDF\Legal\license directory.
- Review <National Instruments>\\_Legal Information.txt for information on including legal information in installers built with NI products.

**U.S. Government Restricted Rights**

If you are an agency, department, or other entity of the United States Government ("Government"), the use, duplication, reproduction, release, modification, disclosure or transfer of the technical data included in this manual is governed by the Restricted Rights provisions under Federal Acquisition Regulation 52.227-14 for civilian agencies and Defense Federal Acquisition Regulation Supplement Section 252.227-7014 and 252.227-7015 for military agencies.

**IVI Foundation Copyright Notice**

Content from the IVI specifications reproduced with permission from the IVI Foundation.

The IVI Foundation and its member companies make no warranty of any kind with regard to this material, including, but not limited to, the implied warranties of merchantability and fitness for a particular purpose. The IVI Foundation and its member companies shall not be liable for errors contained herein or for incidental or consequential damages in connection with the furnishing, performance, or use of this material.

**Trademarks**

Refer to the *NI Trademarks and Logo Guidelines* at ni.com/trademarks for information on National Instruments trademarks. Other product and company names mentioned herein are trademarks or trade names of their respective companies.

**Patents**

For patents covering the National Instruments products/technology, refer to the appropriate location: **Help»Patents** in your software, the patents.txt file on your media, or the *National Instruments Patent Notice* at ni.com/patents.
