# Supplementary material for: Imaging brain activity during complex social behaviors in Drosophila with Flyception2
Source: Nat Commun. 2020 Jan 30;11:623. doi: 10.1038/s41467-020-14487-7 (PMC6992788; doi:10.1038/s41467-020-14487-7)
Supplement: Supplementary file 20 — Supplementary Software 1 [file 41467_2020_14487_MOESM20_ESM.zip › Supplementary Software 1/Flyception2_user_manual.pdf]

Flyception2 user manual

Dhruv Grover, Takeo Katsuki, Jinfang Li, Thomas J. Dawkins, Ralph J. Greenspan

Version 1.0
December 30, 2019

Table of Contents

Setup Assembly 2

Safety precautions 2

System overview 2

Galvo assembly 2

Laser alignment 4

Arena assembly 7

Camera alignment 10

Software installation 11

Prerequisites 11

Hardware configuration on test setup 11

Getting Started 12

Compiling 13

Custom building OpenCV with CMake 13

Compiling custom OpenCV source in Visual Studio 14

Setting up Flyception2 15

Compiling Flyception2 16

Arena-view camera calibration 19

Triggering cameras with LabVIEW and MyRIO 20

Flyception2 tracker 21

Setting Fly-view (Point Grey Gazelle) camera mode 21

Gem Laser Control 23

Dual-color fluorescence video recording with MicroManager 24

Post-acquisition Image analysis on R 25

Setup Assembly

Safety precautions

Wear appropriate protective goggles when lasers are in use.

System overview

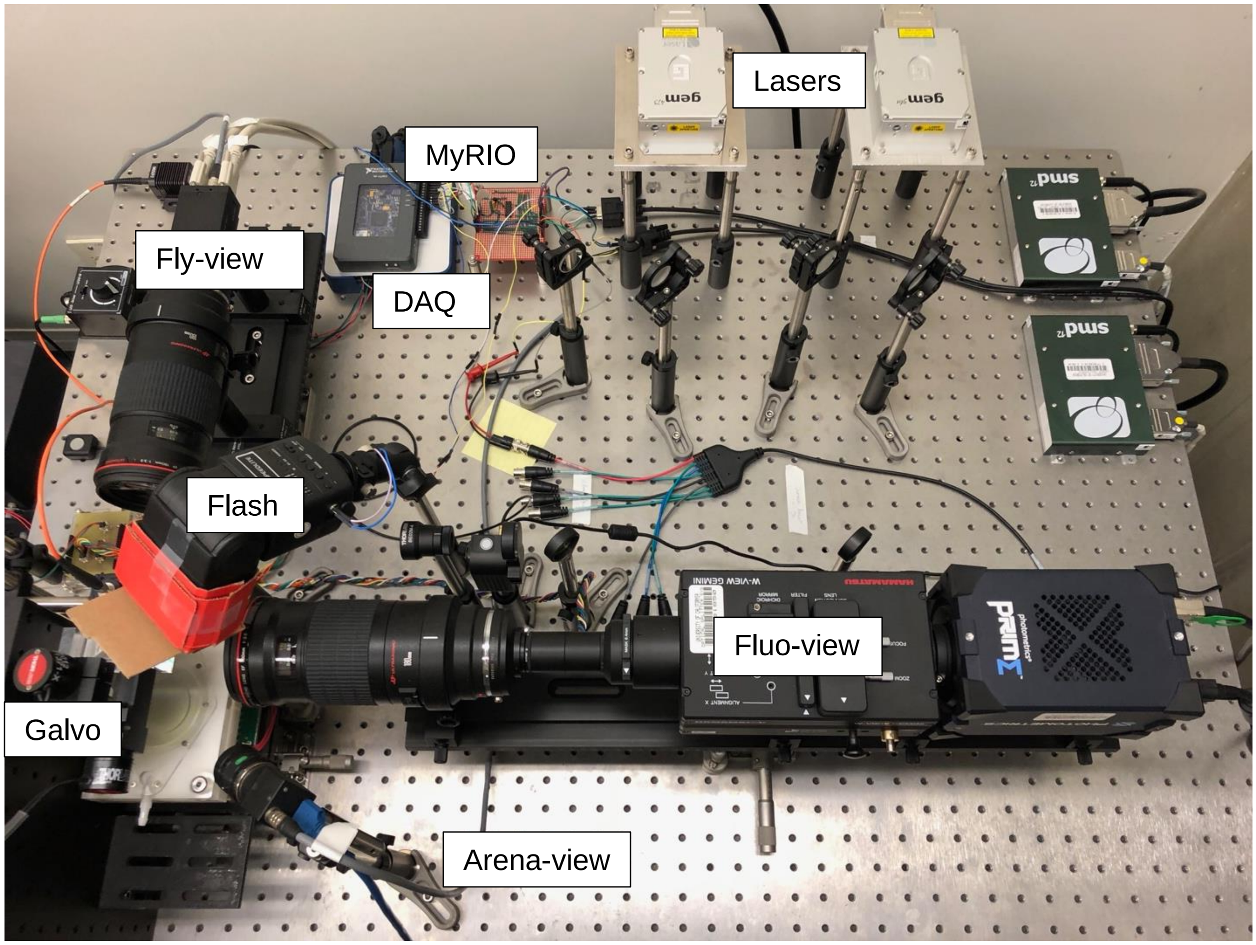

Galvo assembly

1. To determine the exact height of the lasers, start by building the galvo assembly. Attach AP90 brackets to the MB12 breadboard and roughly position the breadboard near the bottom left corner of the isolation table and flush to the bottom edge of the table.

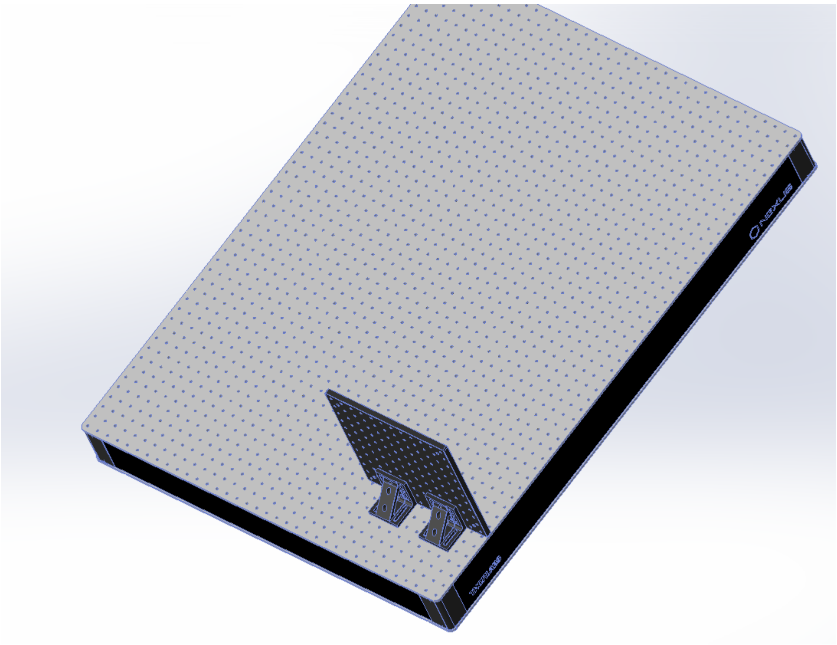

2. Attach the galvo holder to the breadboard as shown below. Measure the height of the holder from the surface of the isolation table at two edges using a height gauge and set it at the same height so that the holder is parallel to the isolation table.

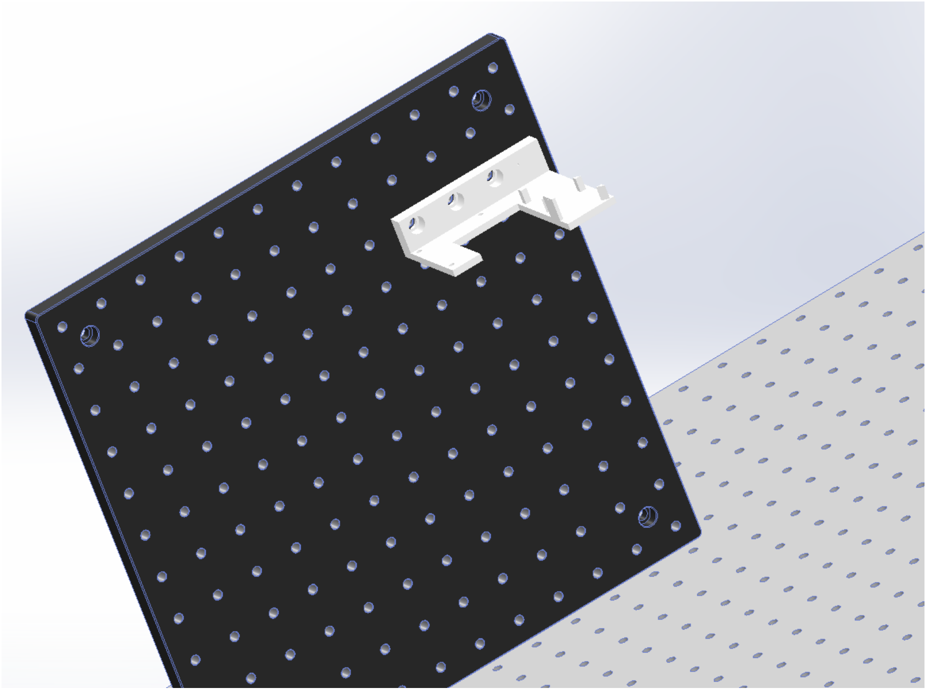

3. Measure the height of the small hole on the galvo holder (below, red arrow). It should be around 242.5 mm from the surface of the isolation table. The hole is at the height equivalent to that of the center of the primary galvo mirror (x-axis mirror).

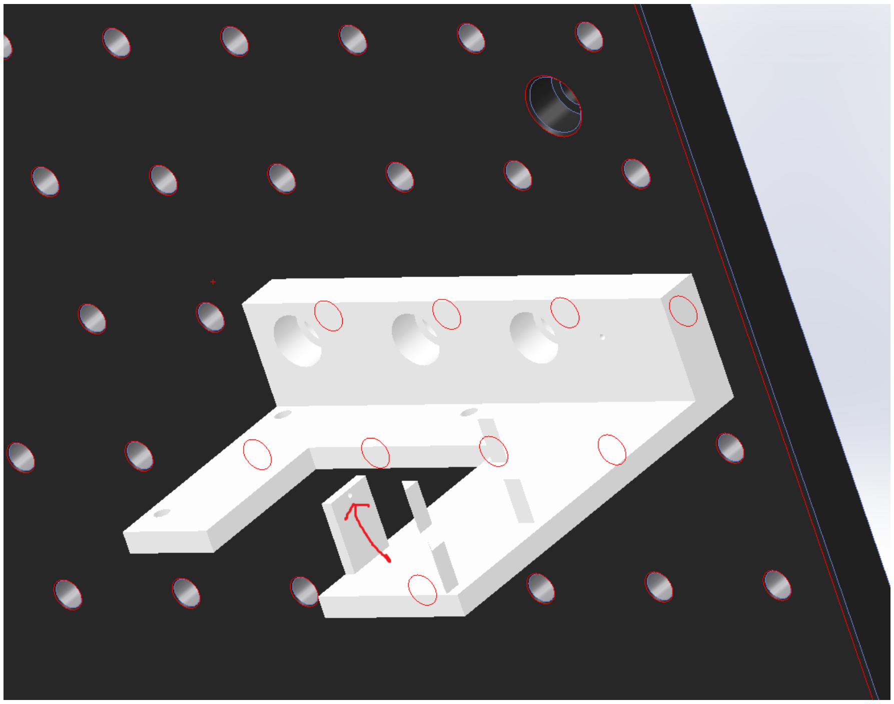

4. Attach the galvo to the galvo holder with screws. Make sure that the back side of the galvo is parallel to the back of the holder by pushing the galvo toward one side of the holder while screwing it.

Laser alignment

5. Place 473 nm and 561 nm Gem lasers on custom laser mounting plates and mount them with 8”+1” posts and 4” post holders.
6. Roughly position the height of the laser aperture at 241 mm from the table surface.
7. Align beams along the holes of the breadboard using two mirrors on kinematics mounts (KM100-E02) and two targets set at the height of the small hole measured at step 3 above (e.g., 241.52 mm). For basic laser alignment techniques, see the following link: https://www.edmundoptics.com/resources/application-notes/lasers/simplifying-laser-alignment/
8. Fold the blue beam 90 degrees to the left along the holes using a 520nm dichroic mirror mounted on an LMR1 mirror holder and a clamp. This ensures that the green beam hits the dichroic at 45 degrees at the next step (note that a transmission curve of dichroic mirrors is angle dependent).
9. Fold the green beam 90 degrees to the left with a mirror mounted on an LMR1 and a clamp. Adjust the position of the mirror such that the green laser through the dichroic mirror completely overlaps with the blue laser. Now the two lasers are combined. Do not place a beam expander yet.

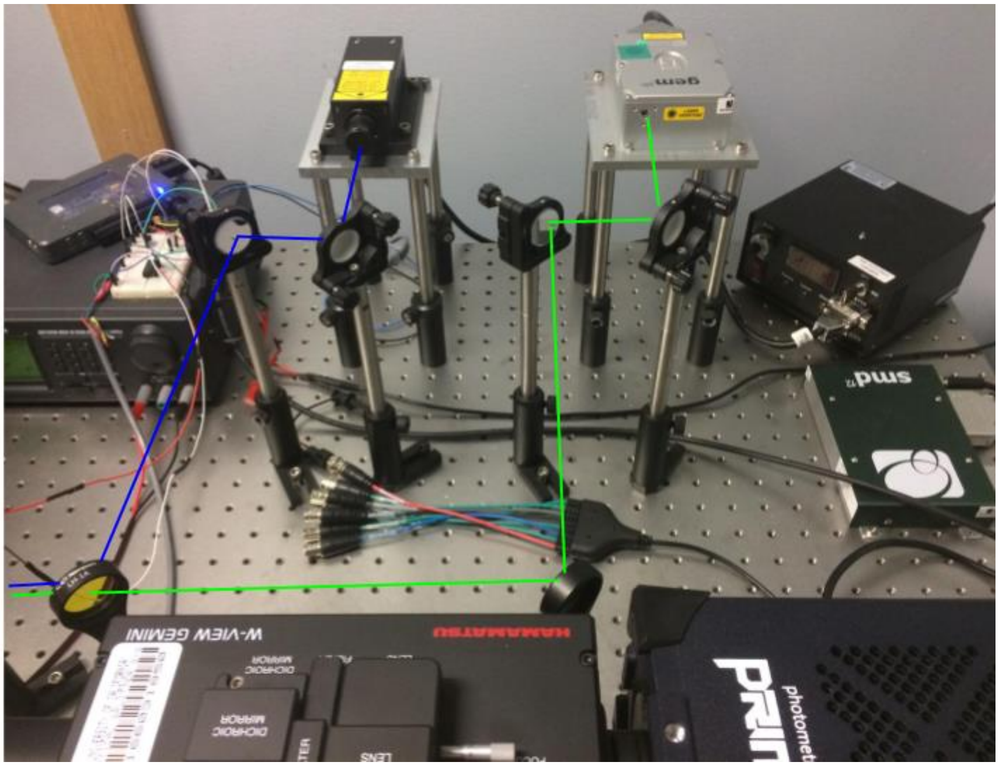

10. Fold the combined beam by 90 degrees toward the bottom with a mirror mounted on an LMR1 and a clamp. Use two targets to align the beam along the holes. Move the galvo assembly if it is in the way of the laser. This will determine the position of the rest of the components including the galvo, fly arena, and cameras.

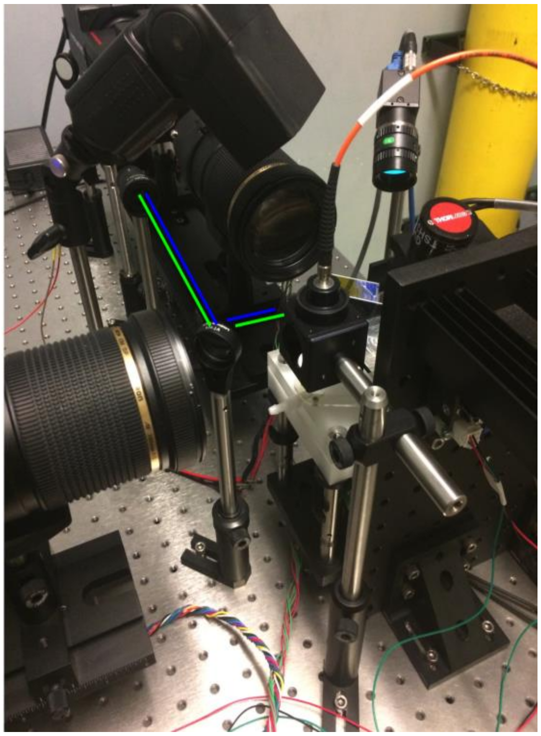

11. Move the galvo assembly back to the path of the lasers and adjust the position of the assembly such that the lasers pass through the alignment hole of the galvo holder. Make sure that the breadboard of the galvo assembly is parallel to the laser using a square. Screw down the brackets.

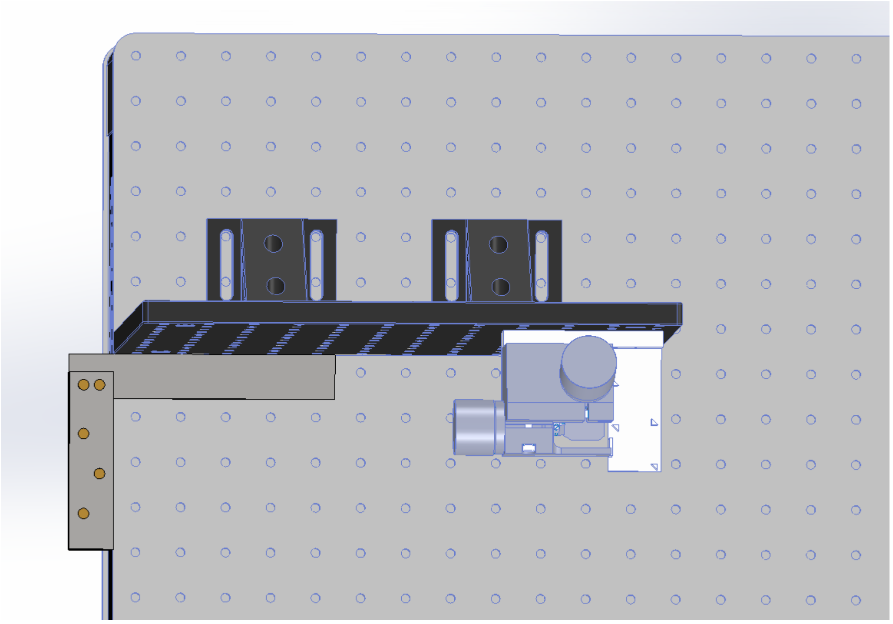

12. Attach a Di01-R488/561-25x36 dichroic mirror to the galvo holder. Secure the mirror to the mirror holder with a small amount of silicone rubber (KE45W, Shin-Etsu silicone) on the edges of the dichroic mirror. This minimizes the strain to the mirror.
13. Check that the laser bent by the dichroic mirror hits the alignment hole on the back of the galvo holder.
14. Attach a Di03-R660-T1-25X36 dichroic mirror to the galvo holder. Minimizing strain is particularly critical for this second dichroic mirror as the reflected light is imaged with the camera for fluorescence imaging.

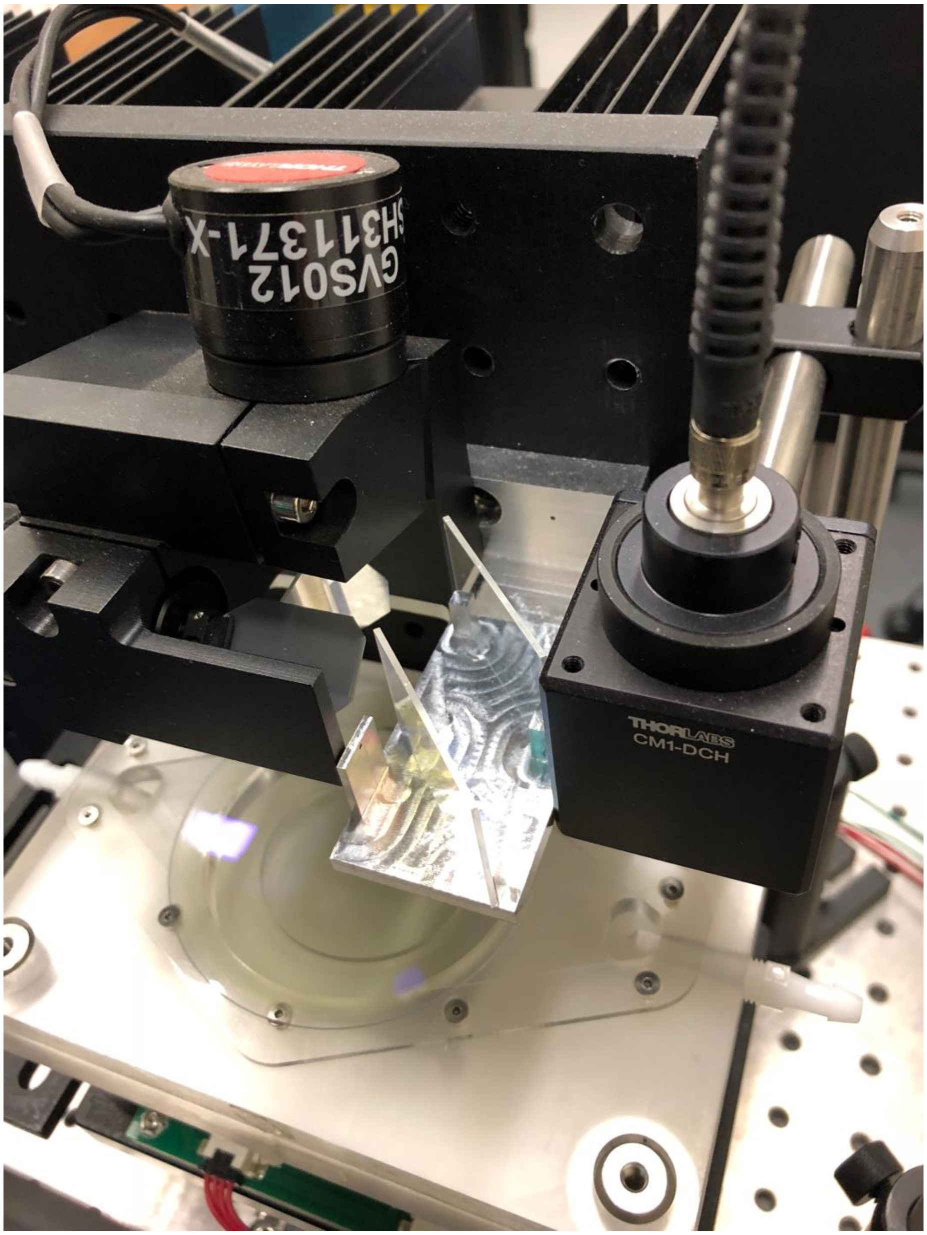

Arena assembly

15. Assemble the fly arena holder with the Newport XYZ stage, Newport 38 baseplate, an MB4 breadboard, and 4x 3” posts. Add 4x 1”+0.75” posts in between the 3” posts along the short edges of the breadboard.
16. Place the fly arena holder under the galvo assembly.
17. Insert the custom Delrin diffuser into the outer posts of the fly arena holder such that it rests on the inner posts.
18. Insert a custom fly arena to the outer posts on top of the diffuser.

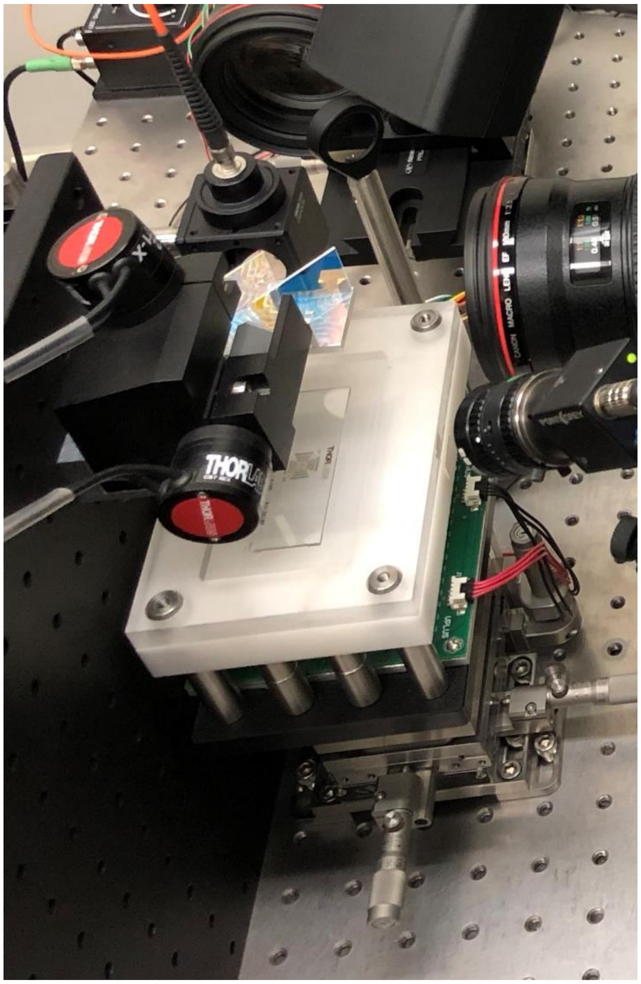

19. Measure the height of the top surface of the arena and adjust it to 184.28 mm by moving the XYZ stage micromanipulator along the z-axis. The height of the top surface of the acrylic chamber should be 241.52 + sin(15deg)*15.174 - 68.167 + 7 = 184.28, where 241.52 is the height of the x mirror center, or the height of the laser, 15.174 is the distance between the x and y mirror centers, 68.167 is the distance between the y mirror center and the arena center, 7 is the distance between the arena plate surface and the center of the arena.
20. Replace the arena with a custom resolution target holder.
21. Move the arena assembly such that the laser hits the center of the target. Eyeballing is sufficient at this step.
22. Assemble the fly-view camera on a PRL-12 rail and a 562-XYZ stage. Level the camera and the lens with a digital angle gauge. Note that a long-pass filter is installed inside a SM1L03 lens tube.

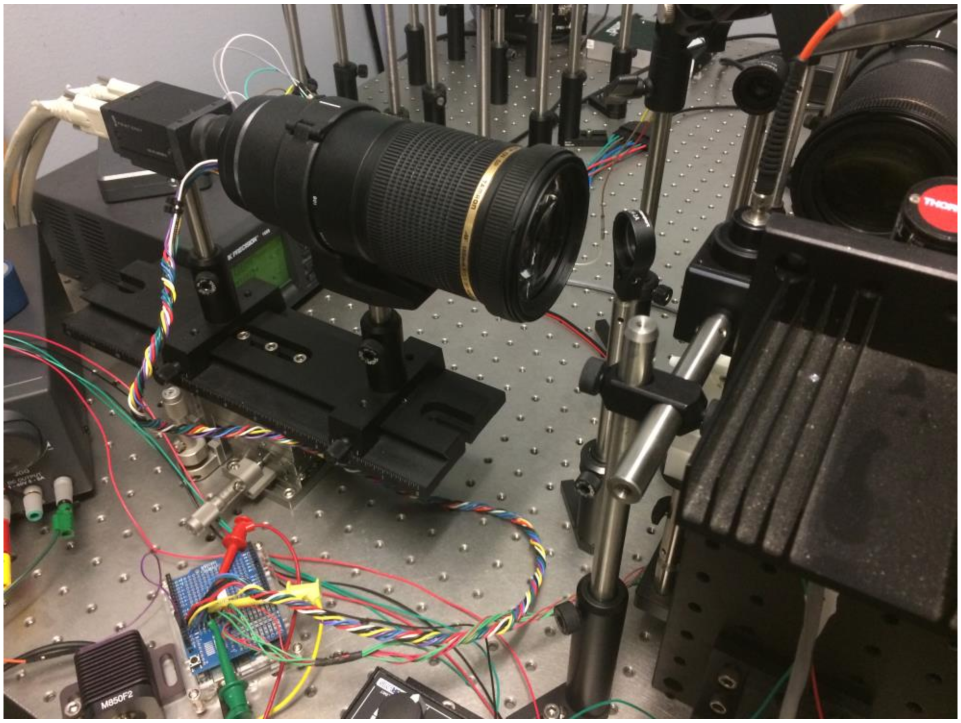

23. Assemble the fluo-view camera on a PRL-24 rail and a 562-XYZ stage. The Gemini is supported with a BA2 and a post from underneath.
24. Install a dichroic mirror (FF580-FDi01-25x36) and filters (FF01-520/35-25 and FF01-607/36-25) in the HAMAMATSU Gemini following the manufacturer’s instruction. Do not overtighten the screws that hold the dichroic mirror.

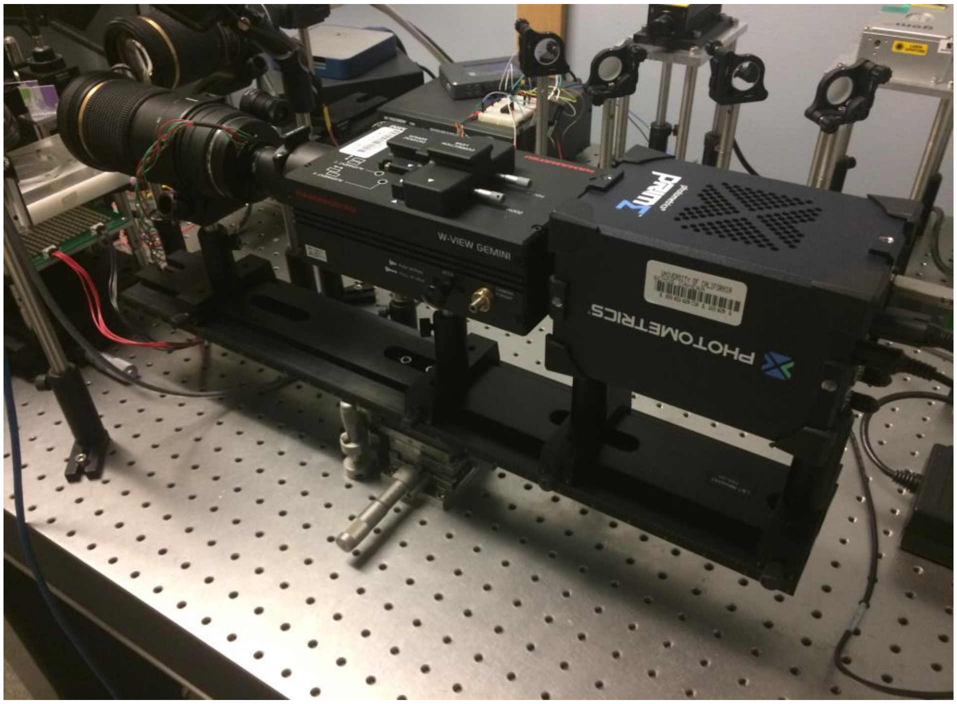

25. Assemble the overhead IR illumination and place it next to the galvo holder, flush to the edge of the galvo holder.

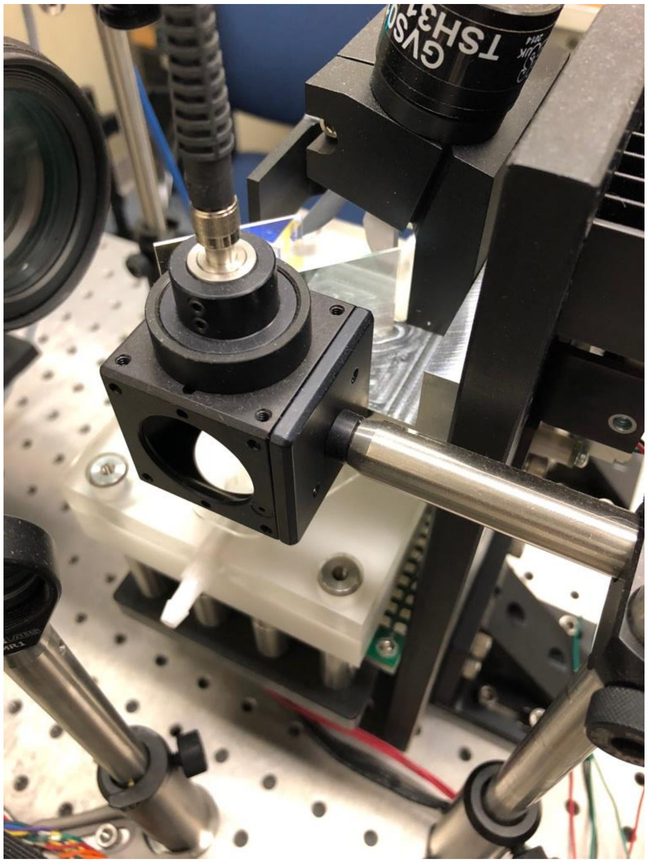

Camera alignment

26. Run the fly-view camera with Teledyne DALSA Sapera SDK. See Point Gray Gazelle camera configuration below for setting up the camera.
27. Place the custom 850nm LED array under the arena.
28. Connect the LED array to a DC power supply and provide 14V, 0.1 mA constant current.
29. Adjust the position of the fly-view camera using the 562-XYZ stage such that the resolution target is at the center of view and in focus.
30. Run the fluo-view camera with MicroManager and with overhead white light (flashlight). Follow the manufacturer's instruction for how to start up the camera. In the case of a Photometrics' Prime camera connected to a PCIe, do the following:
    a. Turn off the PC.
    b. Turn on the camera and wait for about one min until the indicator on the camera's back panel stops blinking.
    c. Turn on the PC.
31. Adjust the position of the fly-view camera using the 562-XYZ stage such that the resolution target is at the center of view and in focus.

32. Adjust Gemini according to the manufacturer's instruction. The image of the target should look like that below. If the image is severely distorted or blurry, check that dichroic mirrors are not strained.
33. Now both fly-view and fluo-view are roughly in the right positions. Fine adjustment will be done in the next steps.
34. Turn off the white LED and turn on the lasers. Set exposure to 100 ms and adjust the position of the arena such that the laser points the center of the target in the fluo-view camera.
35. Once the laser is at the center, add the beam expander to the beam path between the 520nm dichroic mirror and the mirror near the fly-view lens. Looking at the fluo-view image, adjust the height and angle of the expander such that the expanded beam hits the center of the target.
36. Adjust the position of the fly-view camera again using the 562-XYZ stage such that the resolution target is at the center of view and in focus.
37. Now the arena, fly-view camera, and fluo-view cameras are all aligned.
38. Place the arena-view camera on a post looking down the arena at an angle. Adjust the position of the arena-view camera while monitoring the image of the arena on FlyCapture software such that the whole arena fits in a centered image size of 512x512 pixels.

Software installation

Prerequisites

PC running Microsoft Windows 7 64-bit
Microsoft Visual Studio 2013 Professional Edition
CMake
OpenCV library
Point Grey Flycapture SDK 64-bit
Photometrics Prime Camera SDK
MicroManager
Teledyne DALSA Sapera LT SDK
NI-DAQmx
NI-LabVIEW
Laser Quantum Remote App
Arduino

Hardware configuration on test setup

Dell Precision T7810 Workstation (Dual 10-core 2.8GHz)
128GB RAM
2x512GB SSD drives, first for OS and second for video recording
Teledyne DALSA XCelera PX4 full frame-grabber PCIe card

National Instruments PCIe-6351 DAQ
Point Grey 2-port USB3 PCIe card
Photometrics Prime PCIe card
Arduino Uno Rev3
National Instruments MyRIO

Getting Started

Download and install Microsoft Visual Studio 2013. For academic use, free licensed copies of the software are available on dreamspark.com.

Download and install CMake v3.6.2 or above (https://cmake.org/).

Download OpenCV source code (v3.1.0 or above required) from GitHub (https://github.com/Itseez/opencv). OpenCV for windows provided on opencv.org can also be downloaded as it includes the source code along with a default build, however, the flyception software requires a custom build of OpenCV.

Download and install the latest version of Point Grey Flycapture SDK 64-bit (v2.8 or above) from (https://www.ptgrey.com/support/downloads).

Download and install the latest version of Photometrics Prime drivers (PVCAM 3.7.5.7 or above) from (https://www.photometrics.com/support/software/#software). The software is included with the camera as well.

Download and install the latest version of MicroManager (https://www.micro-manager.org/).

Download and install the latest version of National Instruments DAQmx software from (http://sine.ni.com/nips/cds/view/p/lang/en/nid/10181).

Download and install National Instruments LabVIEW development software (2016 or later) from (http://www.ni.com/download/labview-development-system-2016/6055/en/).

Download and install the Sapera LT SDK from Teledyne DALSA (https://www.teledynedalsa.com/imaging/products/software/sapera/lt/). An account creation step might be required for software download. This software is included with the framegrabber card.

Download and install the Laser Quantum Remote App from (https://www.laserquantum.com/products/options-customisations/detail.cfm?id=1).

Download and install the latest Arduino IDE environment from (https://www.arduino.cc/en/Main/Software).

Compiling

Custom building OpenCV with CMake

The following instructions are similar to those provided by OpenCV at (http://docs.opencv.org/3.0-beta/doc/tutorials/introduction/windows_install/windows_install.html)

Unzip the OpenCV source code to C:\SDK\opencv-3.1.0\sources\ folder
Create a folder C:\SDK\opencv-3.1.0\build\ to hold the custom-built binaries
Run the cmake-gui program
Enter the full paths for the OpenCV source code and binaries
Select the grouped and advanced options and press configure
Select Visual Studio 12.0 (2013) Win64 as the compiler of choice
Uncheck BUILD->DOCS
Check WITH->WITH_OPENMP
Press configure again and verify that OpenMP module has been loaded
Press configure again to include OpenMP and then press generate

The source code for the OpenCV library with OpenMP support for Visual Studio 2013 will now be saved to the binaries folder indicated in CMake.

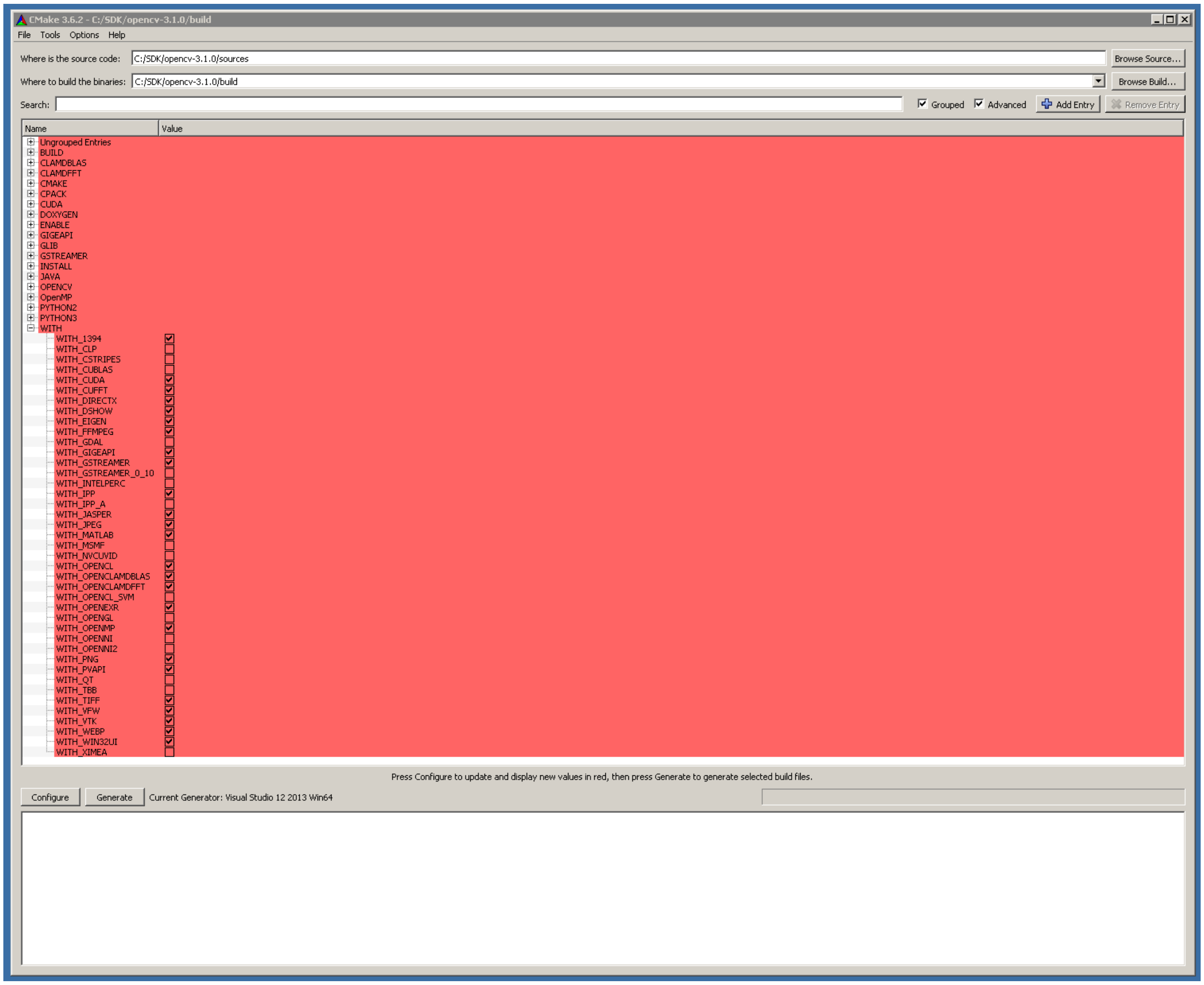

Compiling custom OpenCV source in Visual Studio

Navigate to C:\SDK\opencv-3.1.0\build and run the OpenCV.sln visual studio solution file

Once loaded, select the ALL_BUILD project and use the RELEASE solution configuration, and x64 solution platform. Select the BUILD menu item and then build ALL_BUILD

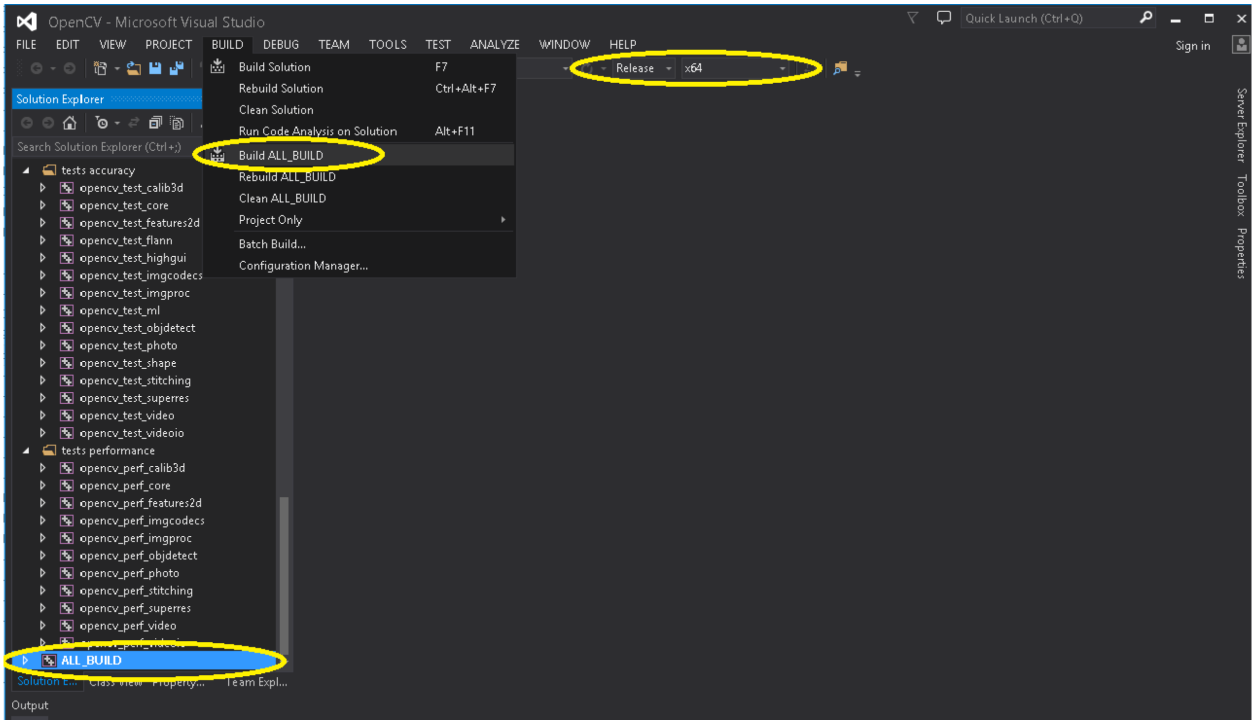

OpenCV should now begin compiling. This process may take a few minutes.

Once compiled, select the INSTALL project and then the BUILD menu item followed by build INSTALL. This installs the OpenCV binaries built to the folder C:\SDK\opencv-3.1.0\build\install\

Custom binaries of OpenCV with OpenMP support are now built and are saved to the following folders. In the next section, we will be including these folder paths when compiling the flyception source code

INCLUDE PATH - C:\SDK\opencv-3.1.0\build\install\include
LIB PATH - C:\SDK\opencv-3.1.0\build\install\x64\vc12\lib
BIN PATH - C:\SDK\opencv-3.1.0\build\install\x64\vc12\bin

Note: Path to the OpenCV bin folder needs to be included in the SYSTEM Path. This can be achieved by navigating to Control Panel -> System -> Advanced System Settings -> Environment Variables -> System Variables -> Path. Add the aforementioned BIN PATH to the path string. A restart of Windows may be required for the system path to be refreshed.

Setting up Flyception2

Download the latest version of the Flyception2 source code from https://github.com/dgrover/flyception2.

The Flyception2 source code has the following folder hierarchy:

- Flyception2 - root folder.

- Flyception2\myRIO – source code to be installed on the NI myRIO FPGA for camera and flash triggering.
- Flyception2\canon_lens_control – source code to be installed on the Arduino Uno for lens control of fly-view and fluo-view cameras.
- Flyception2\flybam - source code for the tracking program.
- Flyception2\calibration - scripts to calibrate the arena-view camera to convert pixel coordinates to real-world coordinates.
- Flyception2\ccf - camera configuration files for Point Grey Gazelle camera (fly-view) and Photometrics Prime (fluo-view).

Compiling Flyception2

Navigate to the Flyception2\flybam folder and run the flybam.sln solution file.
Verify that the x64\RELEASE solution platform is selected.

The provided project file includes the path to all prerequisite libraries, however, if custom folders were used, the library paths need to be changed here.

Navigate to Project -> flybam properties -> configuration properties -> vc++ directories
Verify the following directories are included

Include directories
C:\Program Files\Point Grey Research\FlyCapture2\include
C:\SDK\opencv-3.1.0\build\install\include
C:\Program Files x86\National Instruments\Shared\ExternalCompilerSupport\C\include
C:\Program Files\Teledyne DALSA\Sapera\Examples\Classes\Common
C:\Program Files\Teledyne DALSA\Sapera\Classes\Basic
C:\Program Files\Teledyne DALSA\Sapera\Include

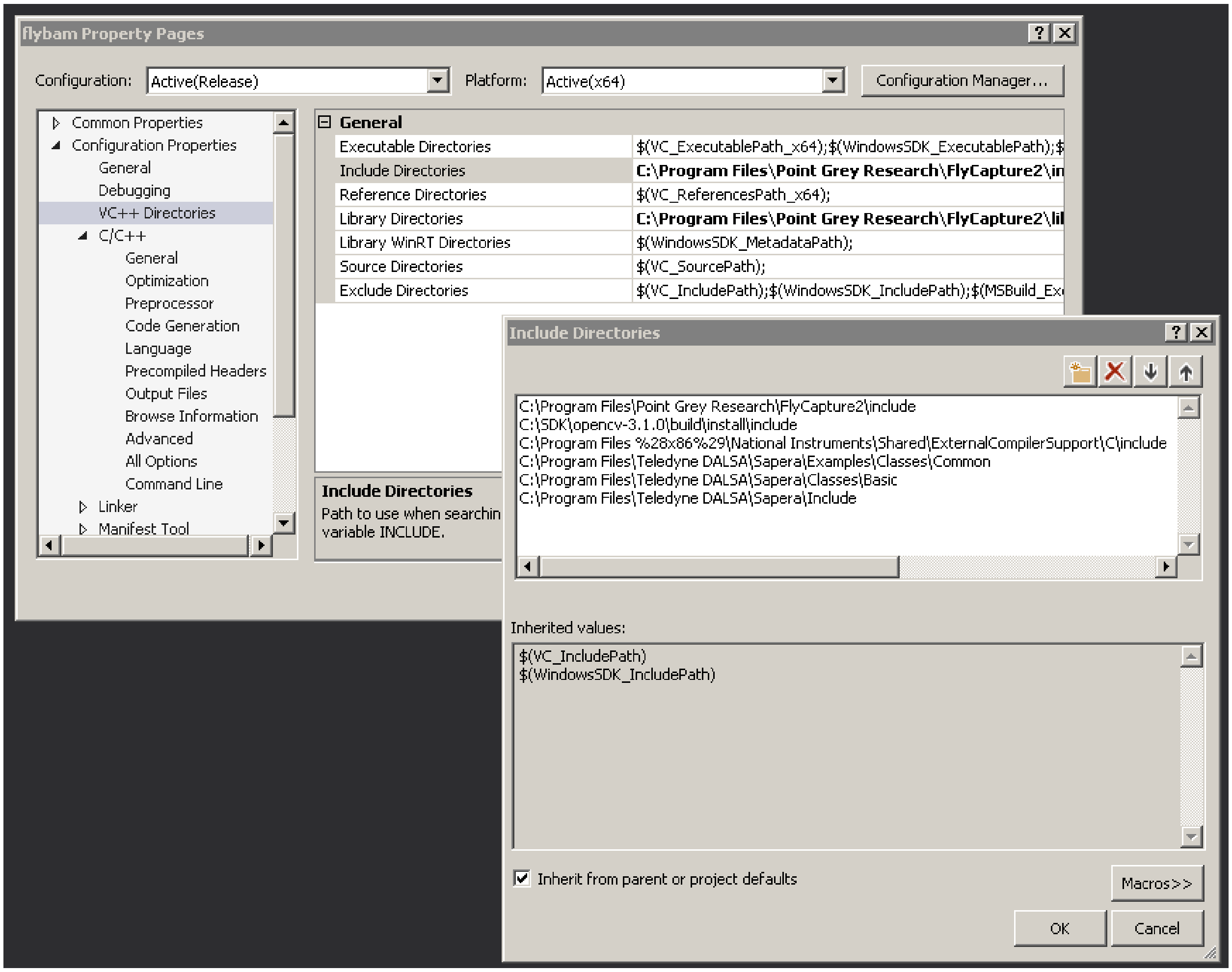

Library directories
C:\Program Files\Point Grey Research\FlyCapture2\lib64
C:\SDK\opencv-3.1.0\build\install\x64\vc12\lib
C:\Program Files %28x86%29\National Instruments\Shared\ExternalCompilerSupport\C\lib64\msvc
C:\Program Files\Teledyne DALSA\Sapera\Lib\Win64

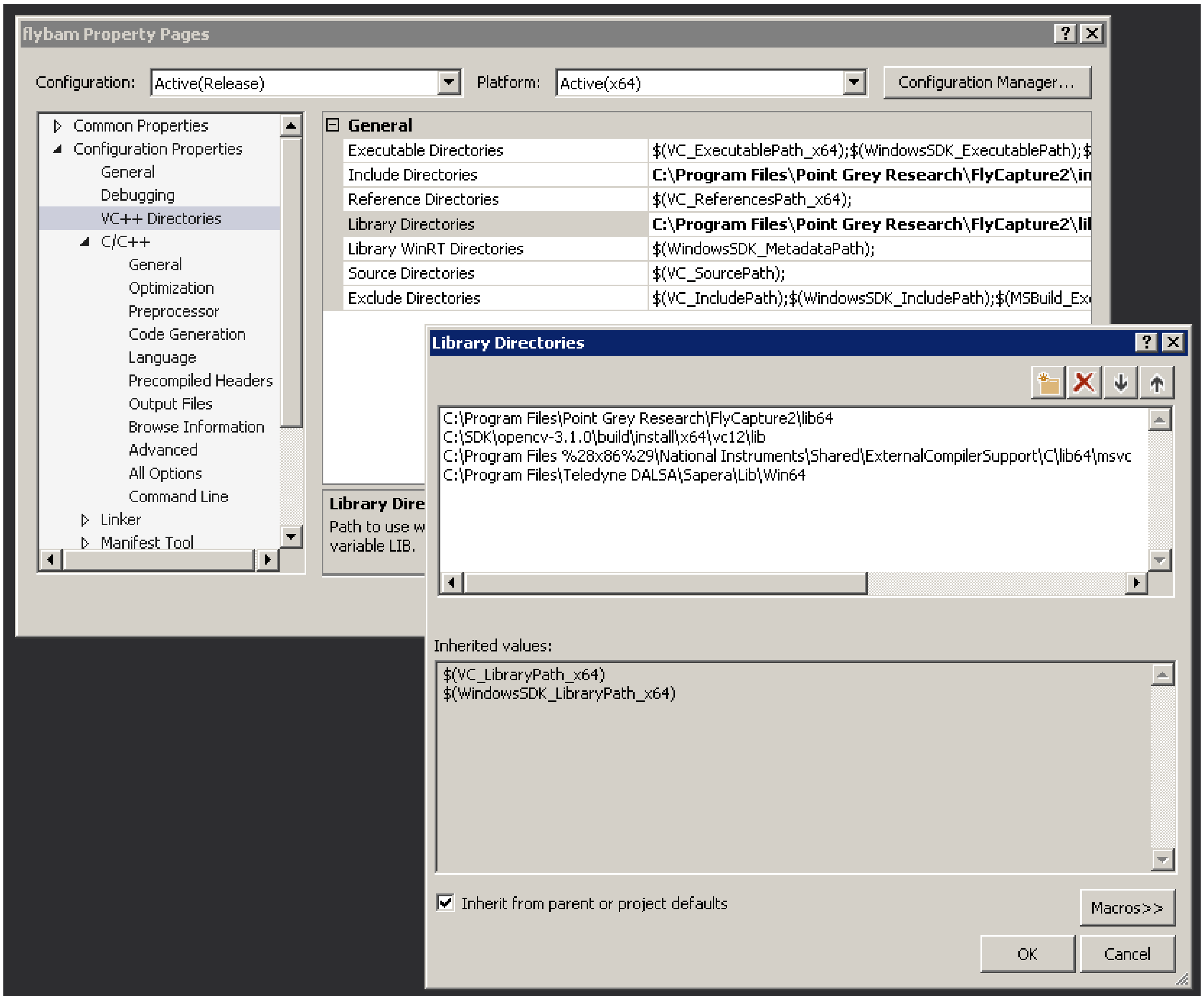

Under Linker -> Input, verify the following .lib files are included

nidaqmx.lib
FlyCapture2.lib
FlyCapture2GUI.lib
opencv_core310.lib
opencv_highgui310.lib
opencv_video310.lib
opencv_imgproc310.lib
opencv_imgcodecs310.lib
opencv_calib3d310.lib
SapClassBasic.lib

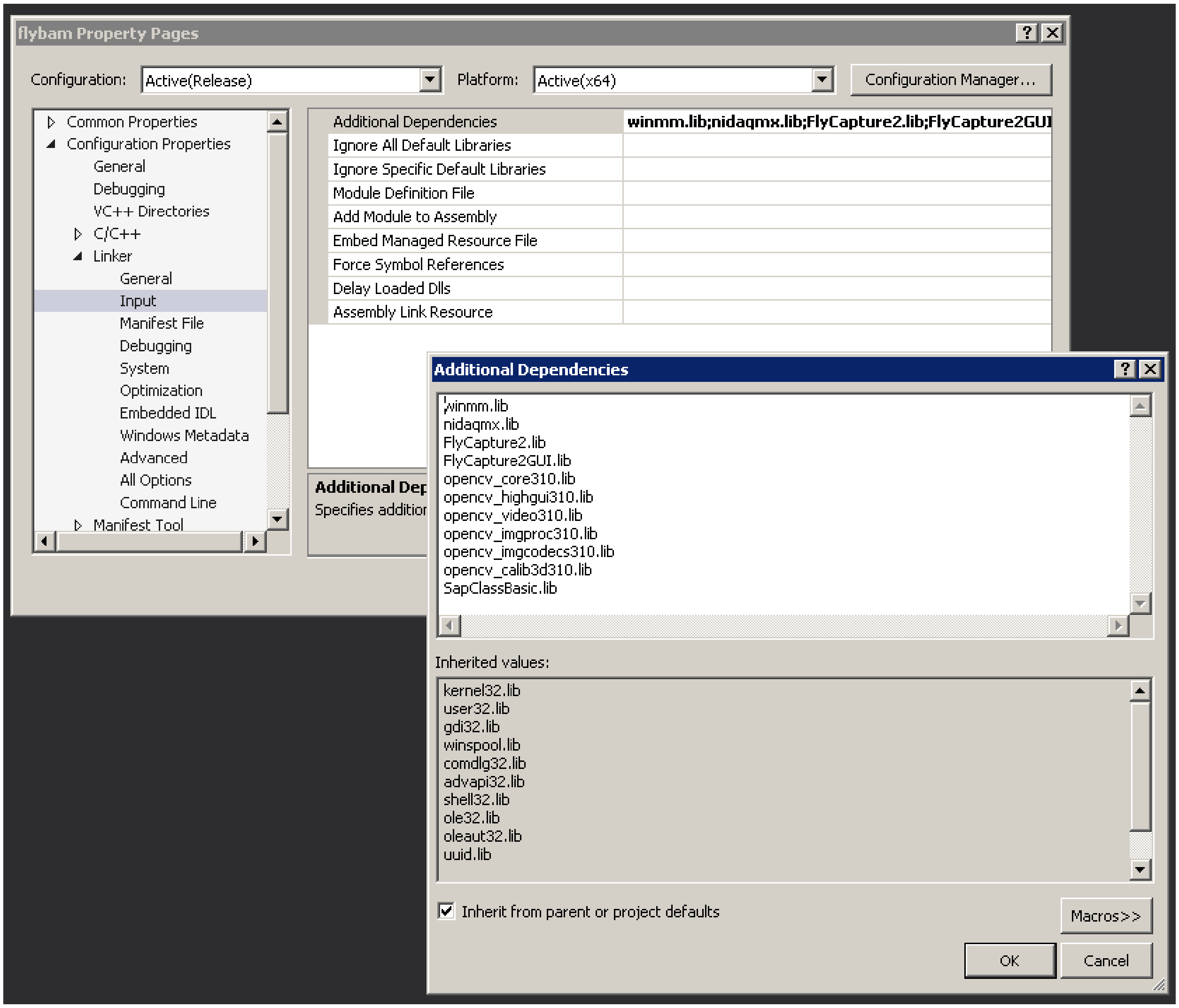

With these folder paths included, the flyception2 software is ready to be compiled and executed.

Arena-view camera calibration

The Flyception2\calibration\raster directory contains a Visual Studio solution that creates a mapping between arena-view image coordinates and real-world coordinates. The principle behind the approach is to raster scan a laser dot on the arena surface for different galvo-mirror angle positions, and simultaneously note the 2D image pixel position of the laser dot center in the arena camera view.

For this purpose, we turn on the 473nm blue laser (with beam expander removed from light path) at low power (~1mW). When the code is executed, a live stream of the arena-view camera is displayed, and the intensity/thresholding of the laser dot can be adjusted to be as tight a spot as possible.

F2 initializes the raster scan from min to max X & Y mirror angles for a defined step size. In our experiments we set the angle range to be [-9.0 9.0] degrees for a step size of 0.1 degrees.

The output is saved in a space delimited text file of the following format per row [*x-angle y-angle x-image-center y-image-center*]. This text file is read into the main tracking program for arena-view tracking.

Triggering cameras with LabVIEW and MyRIO

The Flyception2\myRIO\Flyception2_trigger folder contains the LabVIEW code that needs to be uploaded to the FPGA of the NI MyRIO device for triggering the arena-view, fly-view and fluo-view cameras, as well as the flash.

The Flyception2_trigger.lvproj file opens the project solution in LabVIEW. Navigate to MyRIO device -> Chassis -> FPGA Target and open the Trigger.vi file. Compiling (to FPGA module) and executing this vi file will start sending triggers to the various devices. The code also contains a control panel to set parameters for the various cameras. For instance, fly-view frequency and duty cycle for falling-edge triggering and exposure in bulb shutter mode, arena-view frequency (exposure set in code), and fluo-view frequency (exposure set in micromanager, see below) and delay (as we have determined there to be a time difference between the Photometrics Prime camera receiving the trigger and opening the shutter) to align fluo-view camera exposure with those of arena-view and fly-view cameras.

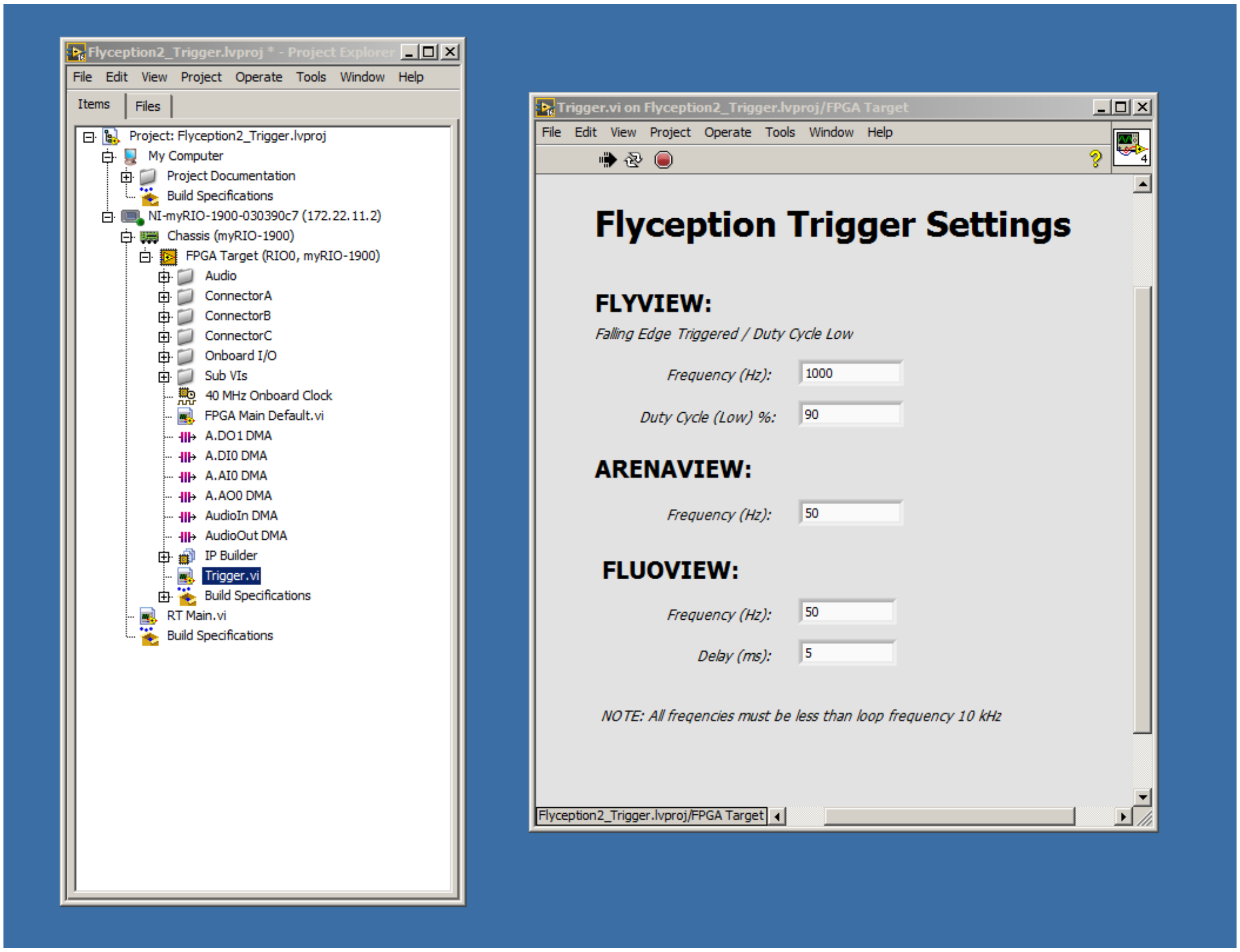

Flyception2 tracker

Setting Fly-view (Point Grey Gazelle) camera mode

Following are the settings required to initialize the fly-view camera with an ROI of 240x240 pixels centered on the sensor, with falling edge external trigger and bulb shutter (simultaneous exposure/readback) mode. The MyRIO labview code will then trigger the camera at the preset frequency (default: 1000Hz) and duty cycle (default: 90% low).

1. (Optional) Unplug and plug the GPIO cable to reboot the camera
2. Open PUTTY and connect to the camera via Serial COM port assigned in Sapera Configuration utility
3. Type dump and return (Device Information), this might need to be done twice if first dump command returns nothing.
4. Type setroi 0 424 240 and return
5. Type w 240 and return
6. Type ox 904 and return

7. Type trsrc GPIO0 and return (trigger source)
8. Type trm bulb and return (bulb mode)
9. Type tra fe and return (falling edge)

When the flyception2 code is executed, the arena-view and fly-cameras are initialized (see screenshot below). The software automatically starts tracking flies in the arena-view video and turns the galvanometer mirrors to follow the first fly tracked. The following keyboard shortcuts used for controlling the software -

F1 - initializes fly-view tracking
F2 - starts/stops recording of arena-view and fly-view images and tracking coordinates. If the fluo-view camera is initialized via MicroManager (see next section), recording of fluo-view videos will also begin.
F3 – recomputes arena-view background image.
F4 – fires flash.
UP, DOWN, LEFT, RIGHT keys - manual control of galvanometer mirror assembly.
HOME – reset galvo-mirror position to center of arena.
TAB – If more than one fly is present in the arena, tab cycles through each fly as the focal one for fly-view tracking.
NUMPAD KEY 1 – Moves both fly-view and fluo-view lenses back by 10 steps. Note, the Arduino code provides the capability to move in varied step increment sizes of 10^x where *[x = 0, 1, 2, 3]* and the key bindings can be changed accordingly.
NUMPAD KEY 2 – Moves both fly-view and fluo-view lenses forward by 10 steps. Note, the Arduino code provides the capability to move in varied step increment sizes of 10^x where *[x = 0, 1, 2, 3]* and the key bindings can be changed accordingly.
NUMPAD KEY 4 – Sweeps both fly-view and fluo-view lenses to their shortest focal distance.
NUMPAD KEY 5 – Sweeps both fly-view and fluo-view lenses to their longest focal distance.
ESC - exits the program.

In addition to the keyboard shortcuts, Flyception2 also allows the user to tweak various tracking parameters via a control panel. For instance, thresholds and morphological operations for arena-view and fly-view tracking, size, shape and position of the edge outline of the arena in arena-view image can be user controlled.

Other parameters such as number of flies, length of videos, arena dimensions, galvo settings, marker size and number can be changed in the stdafx.h file in the flybam directory. If the system is replicated exactly as outlined in this article, most of these settings need not be altered.

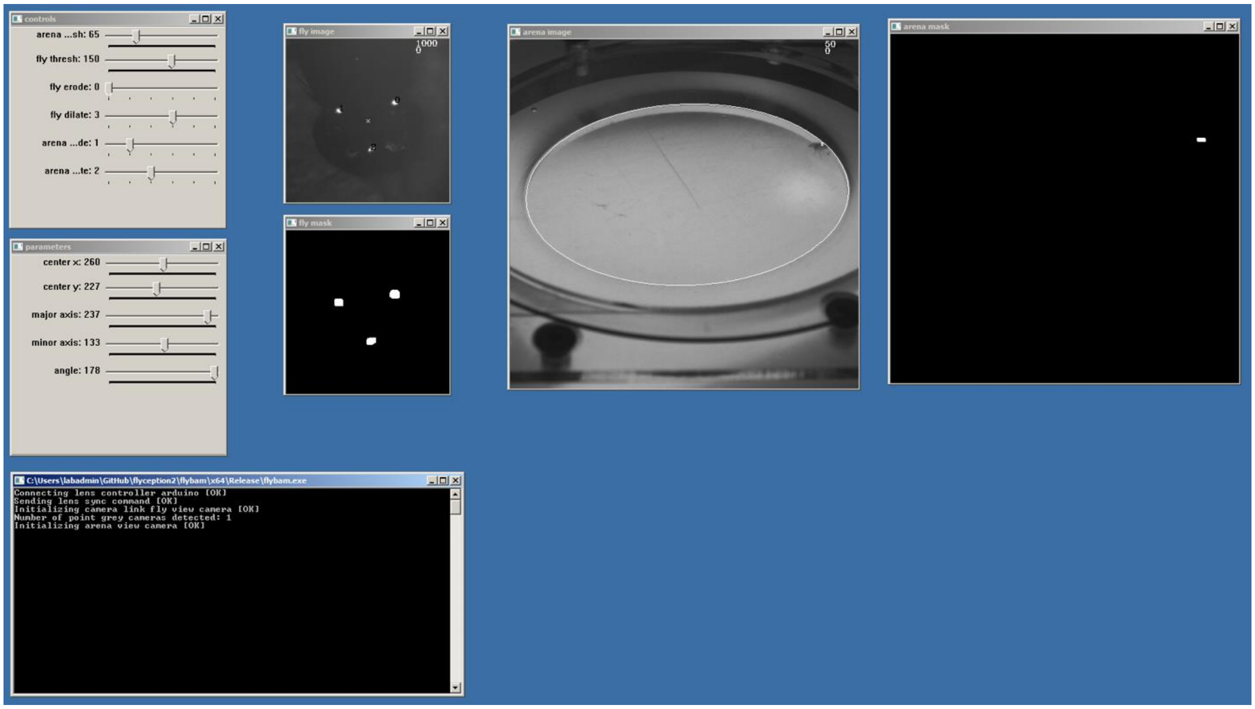

The output of the system after a successful recording session is as follows -

- Arena view video (default length 100s at 50Hz) in fmf format (essentially an uncompressed monochrome avi format)
- Fly-view video (default length 100s at 1000Hz) in fmf format (essentially an uncompressed monochrome avi format)
- Arena view and fly video log files to ensure cameras are functioning at preset frame rates
- Trajectory file, space delimited, with the following parameters per frame at 1000Hz [*Frame Number, Arena x-coordinate, Arena y-coordinate, Fly-view head center x-coordinate, Fly-view head center y-coordinate, Galvo x-angle, Galvo y-angle*]

Gem Laser Control

We run two instances of the Laser Quantum Remote App Laser Control software, one for each of the two Gem lasers (473nm and 561nm) used in our system. Each laser controller (Smd12) is connected to the PC via serial cables and the COM port assignments are set in the Remote App software settings for each instance.

In our experiments, we use the lasers in power mode, and the power levels are set in mW. We use a laser power meter (Thorlabs, PM100D) to determine the appropriate power levels of each laser on the arena surface.

Dual-color fluorescence video recording with MicroManager

Fluorescence images are captured with a Photometrics Prime CMOS camera using MicroManager. We recommend enabling the camera's metadata option, to support accurate timestamp recording for analysis purposes. This can be accomplished by accessing the Device Property Browser under the tools submenu. Set the Prime-MetadataEnabled flag to YES. If desired, this option can be added as a Preset to Configuration settings for easy access. This flag will need to be set each time MicroManager is started.

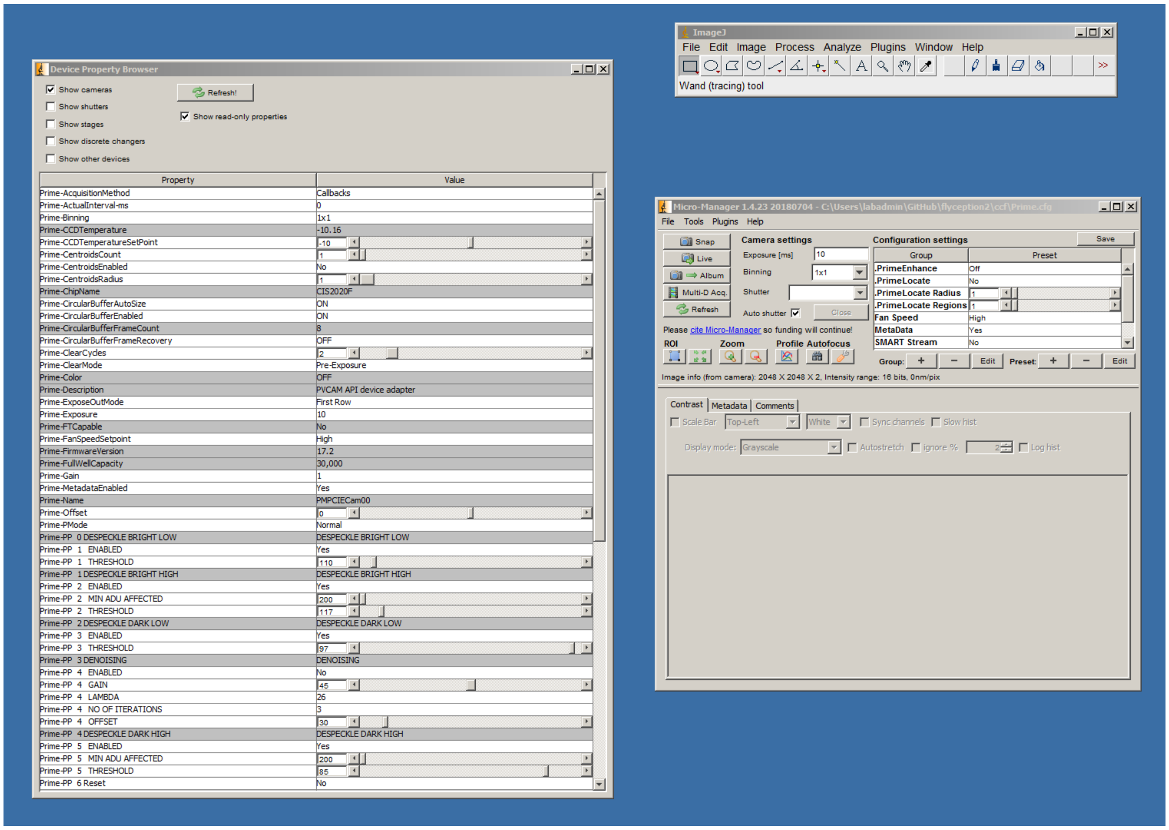

Additional camera settings are controlled in the Configuration settings shown in the screen capture below with the following parameters -

- Trigger Type: Edge trigger (default: 50Hz)
- Fan speed: Low (The camera can be liquid cooled, in which case, the fan can be turned off)
- Exposure: 18ms
- Binning: 1x1
- Image size: 2048x256x2

F2 key needs to be bound to multi-D acquisition function so that the image acquisition starts roughly at the same time as fly-view and arena-view. Number of images to capture is set to 5,000 in Multi-D Acquisition setting.

Start the camera by hitting Live button in the main console, and then start recording with F2 shortcut key. Images will be saved in TIFF format and metadata will be saved in TXT format, with a user-specified filename prefix.

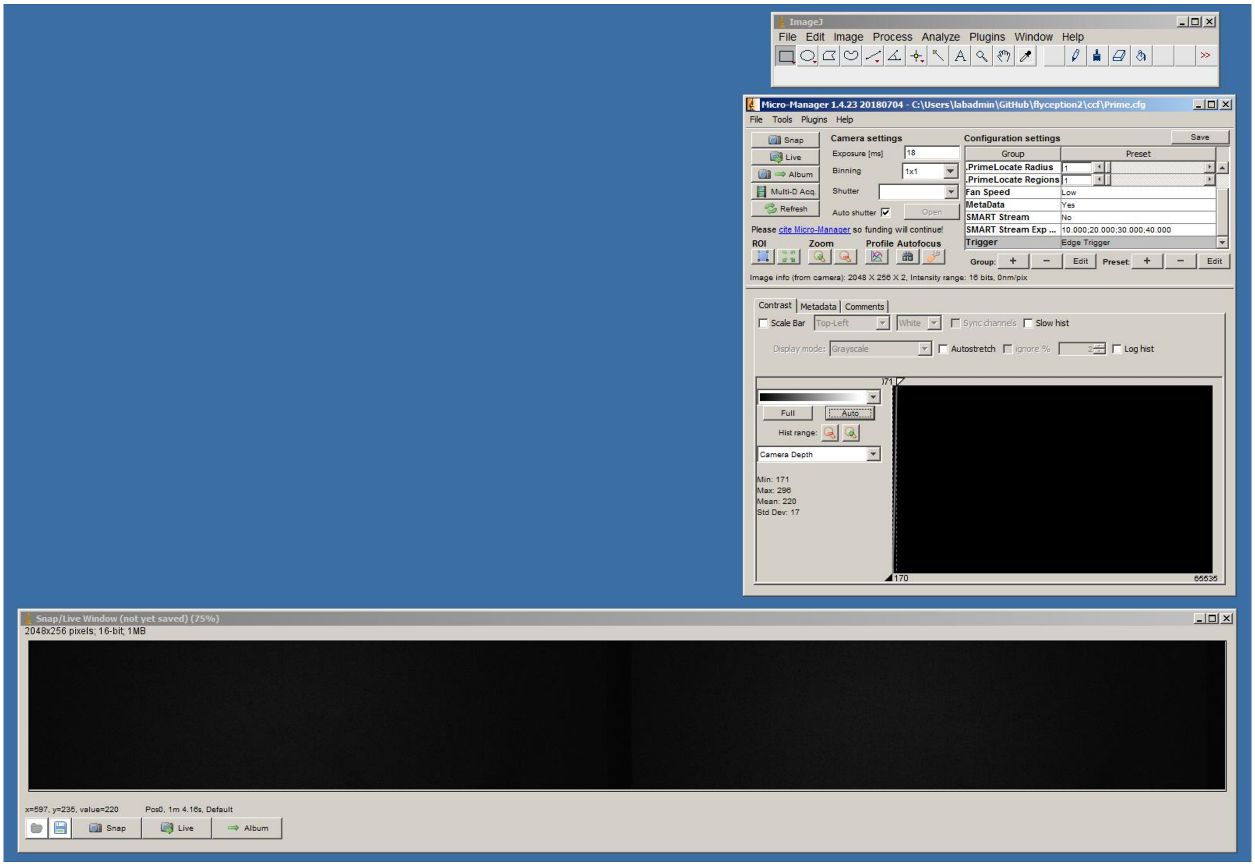

Post-acquisition Image analysis on R

Image registration and quantification of fluorescence changes is done using custom scripts written in R. The scripts and detailed instructions are available from https://github.com/tkatsuki/Flyception2R
