# Supplementary figures and images for: Imaging brain activity during complex social behaviors in Drosophila with Flyception2
Source: Nat Commun. 2020 Jan 30;11:623. doi: 10.1038/s41467-020-14487-7 (PMC6992788; doi:10.1038/s41467-020-14487-7)

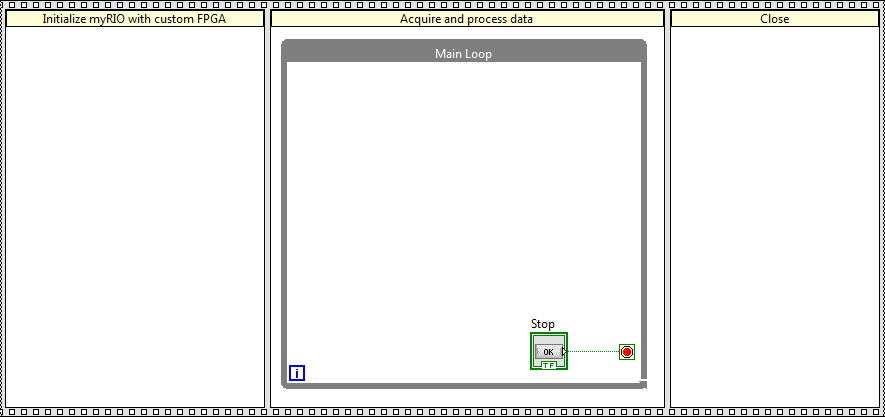

Supplement: Supplementary file 20 — Supplementary Software 1 [file 41467_2020_14487_MOESM20_ESM.zip › Supplementary Software 1/flyception2_tracking/myRIO/Flyception2_Trigger/documentation/custom_fpga_block_diagram.gif]

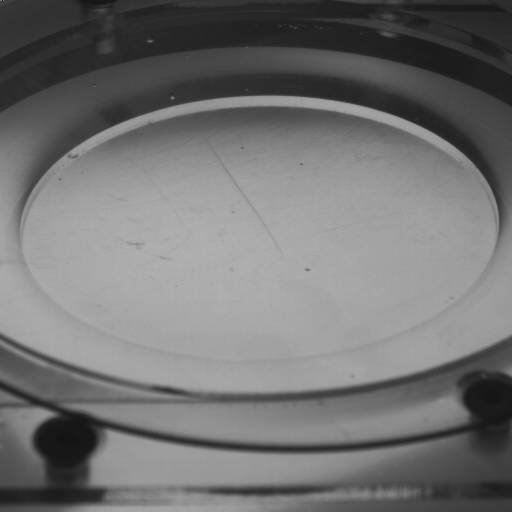

Supplement: Supplementary file 20 — Supplementary Software 1 [file 41467_2020_14487_MOESM20_ESM.zip › Supplementary Software 1/flyception2_tracking/offline/sample_backgrounds/arena_bg_2018.bmp]

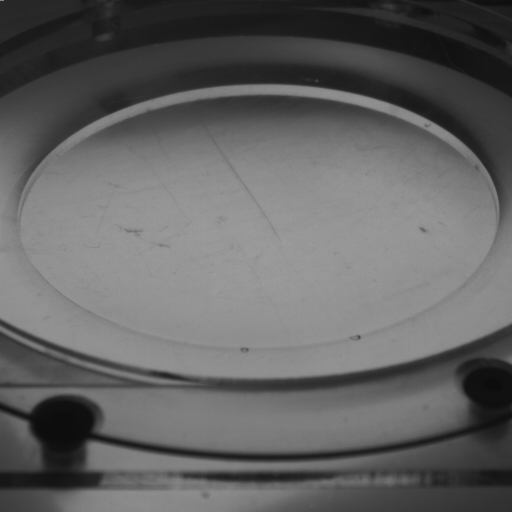

Supplement: Supplementary file 20 — Supplementary Software 1 [file 41467_2020_14487_MOESM20_ESM.zip › Supplementary Software 1/flyception2_tracking/offline/sample_backgrounds/arena_bg_2019.bmp]

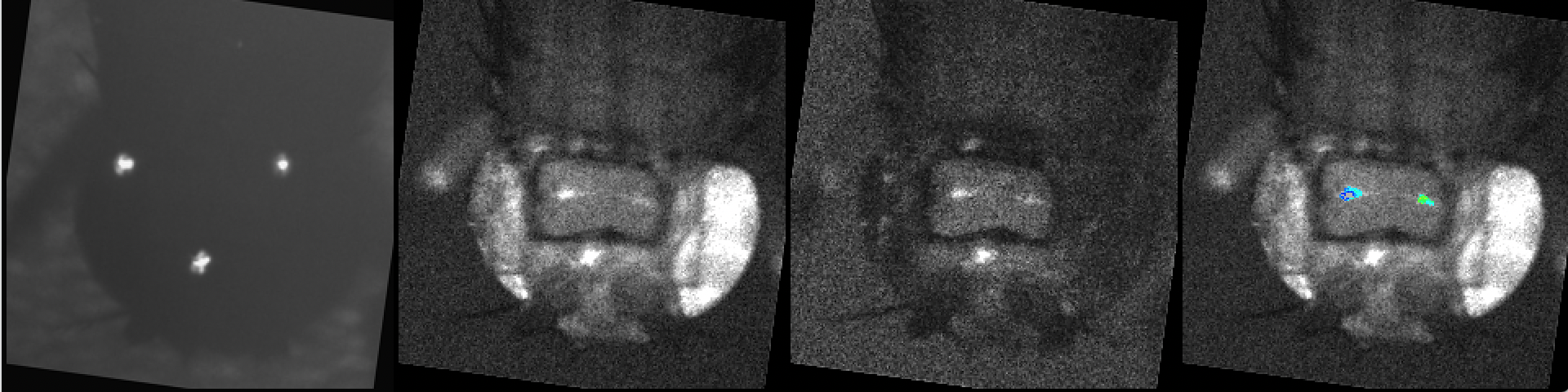

Supplement: Supplementary file 20 — Supplementary Software 1 [file 41467_2020_14487_MOESM20_ESM.zip › Supplementary Software 1/flyception2R_analysis/frgcombined.png]

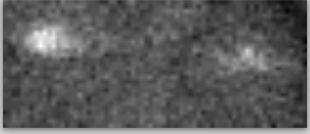

Supplement: Supplementary file 20 — Supplementary Software 1 [file 41467_2020_14487_MOESM20_ESM.zip › Supplementary Software 1/flyception2R_analysis/redwindow.png]
