# Supplementary material for: Imaging brain activity during complex social behaviors in Drosophila with Flyception2
Source: Nat Commun. 2020 Jan 30;11:623. doi: 10.1038/s41467-020-14487-7 (PMC6992788; doi:10.1038/s41467-020-14487-7)
Supplement: Supplementary file 1 — Supplementary Information [file 41467_2020_14487_MOESM1_ESM.pdf]

Imaging brain activity during complex social behaviors in *Drosophila* with Flyception2.
Grover et al.

Supplementary Figure 1

a

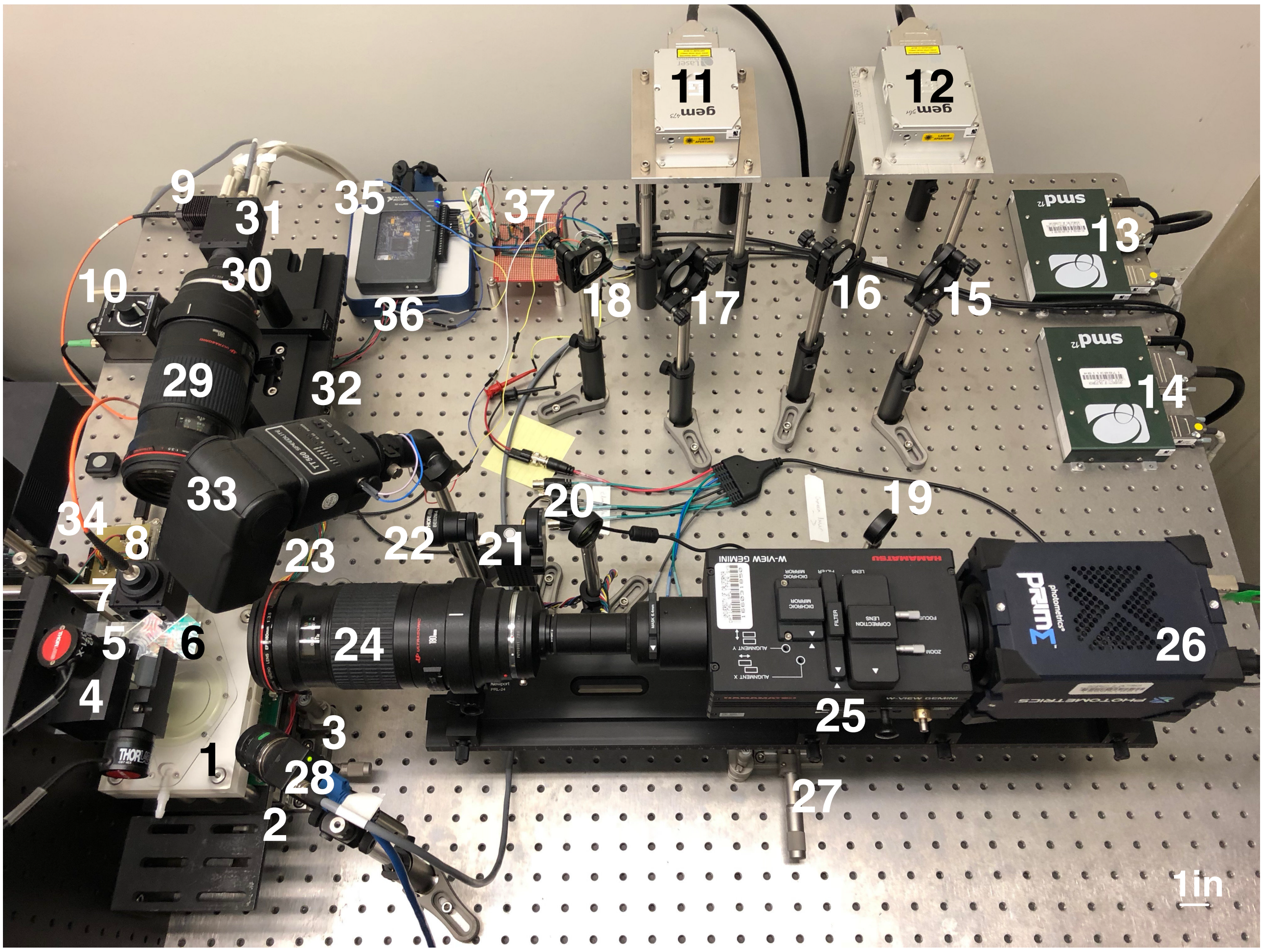

b

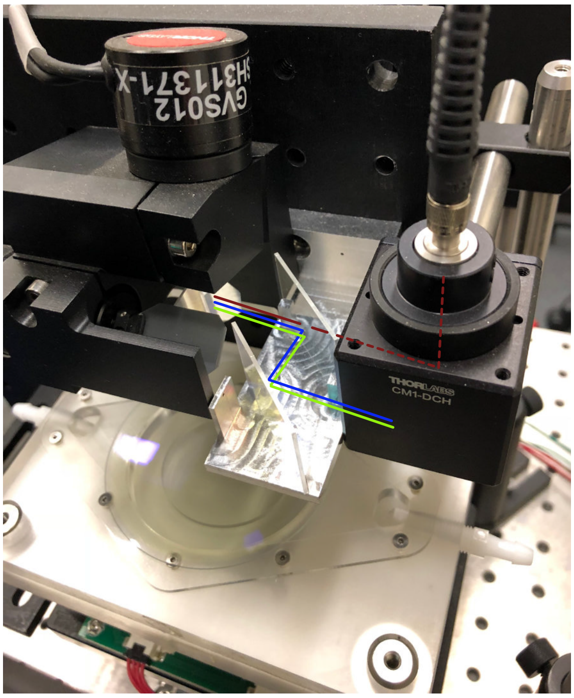

c

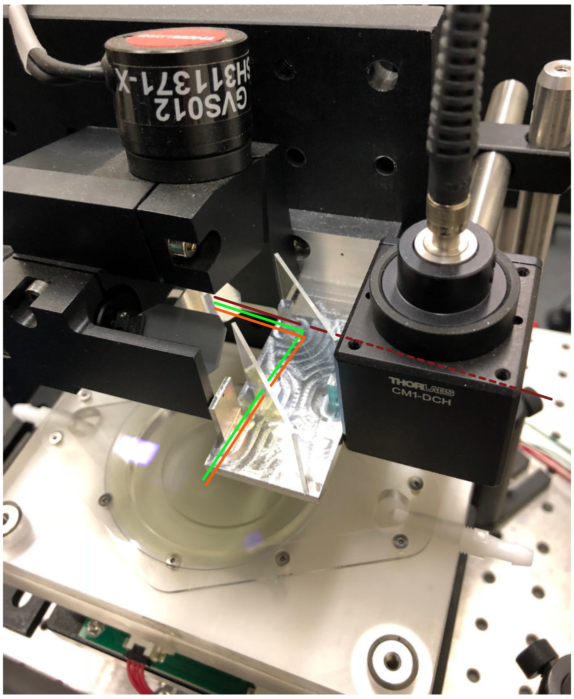

Supplementary Figure 1. Flyception2 system overview. (a) Flyception2 system components, 1) Fly arena with Delrin diffuser backplate, 2) Infra-red backlight, 3) XYZ translational stage, 4) Two-axis galvanometer mirror assembly, 5) Far-red longpass dichroic mirror, 6) Multi-band pass dichroic mirror, 7) 10:90 R:T infra-red beamsplitter mirror, 8) Multi-mode fiber patch cable with collimating lens, 9) 850nm Fiber-coupled LED, 10) LED power controller, 11) 473nm DPSS laser, 12) 561nm DPSS laser, 13 and 14) Laser power control units, 15-18) Kinematic mirrors to reflect and align laser beams, 19) Mirror to reflect laser beam, 20) Dichroic mirror to reflect and merge laser beams, 21) Optical beam shutter, 22) 2x laser beam expander, 23) Optical mirror (occluded in image) to reflect laser beam and align with dichroic and galvo-mirror assembly, 24) Fluo-view camera lens, 25) Dual-view fluorescence emission light splitter with inbuilt dichroic mirror and band-pass filters, 26) Fluo-view camera, 27) Fluo-view camera XYZ translational stage, 28) Arena-view camera with far red-longpass filter, 29) Fly-view camera lens, 30) Far-red longpass filter, 31) Fly-view camera, 32) Fly-view camera XYZ translational stage, 33) Flash unit, 34) Arduino with custom shield for synchronized fly-view and fluo-view camera lens movement control, 35) NI MyRIO for FPGA-based camera and flash triggers, 36) NI PCIe DAQ for analog control of galvo mirrors, 37) Flash trigger circuit. (b) Laser excitation and infra-red retroreflection source light paths being combined through beamsplitter and dichroic mirrors to the rotating two-axis galvanometer mirror assembly and down to the arena and fly head. Blue and green lines represent laser beam paths with beams reflecting off the multi-bandpass dichroic mirror followed by far-red longpass dichroic mirror onto the primary and secondary galvanometer mirrors. In red is the IR fiber LED beam path passing through the far-red longpass dichroic mirror to the galvanometer mirrors. The dotted red line represents the IR fiber LED beam path internal to the filter cube, emanating from the source fiber patch cable, passing through a collimating lens, and reflecting off a 10:90 Reflectance:Transmission beamsplitter mirror. (c) Fluorescence emission and retroreflection light paths from the fly arena through the two-axis galvanometer mirror assembly being split via dichroic and beamsplitter mirrors to fly-view and fluo-view imaging cameras. In green and orange are the emission wavelength light paths for GCaMP and tdTomato channels reflecting off the far-red longpass dichroic mirror and transmitting through the multi-bandpass dichroic mirror to the fluo-view camera. In red is the IR retroreflected light path passing through the far-red longpass dichroic mirror and internally through the filter cube that houses the beamsplitter mirror (dotted line segment) to the fly-view camera.

Supplementary Figure 2

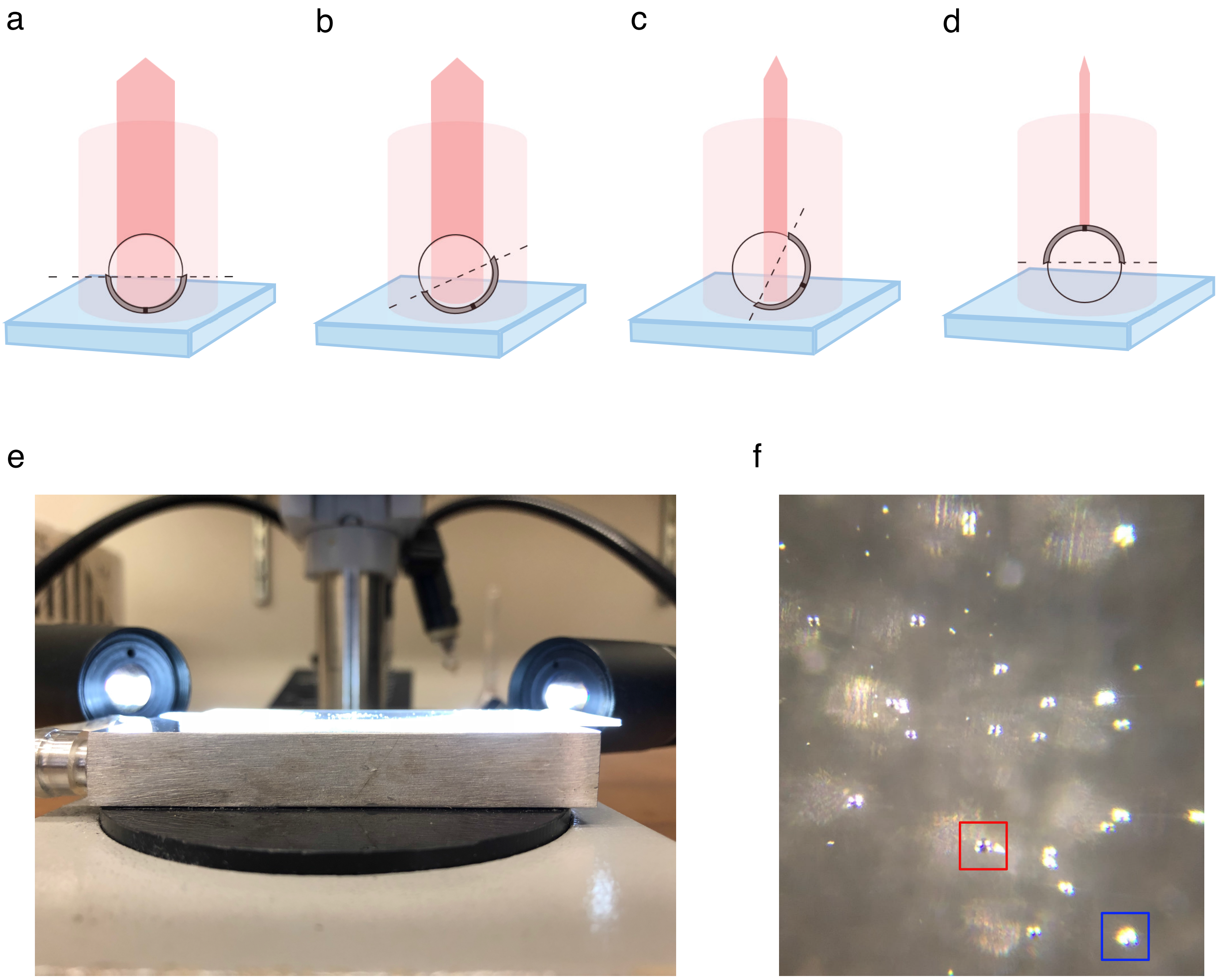

Supplementary Figure 2. Preparing retroreflective bead marker coverslips. Top row illustrates the relationship between orientation of the bead placed on the coverslip and amount of retroreflected light detected by the fly-view tracking camera. In light red is the incoming light beam on the bead from the IR collimated fiber LED light source. In dark red are the light rays reflected off the aluminum half-shell coated bead back up to the fly-view tracking camera. (a) Bead placed in the ideal orientation with glass side up and aluminum half-shell coated side towards the coverslip. This configuration allows maximal retroreflection and yields robust tracking. (b) Tilting of the bead on the coverslip reduces retroreflection and can influence detection and tracking. Minor tilting of the bead presents minimal effect on retroreflection. However, accurate orientation of the bead compensates for pitch axis tilting of the coverslip due to tilting of the fly head. Therefore, an inaccurately oriented bead would reduce that capability of maintaining track of the fly head when tilted. (c) Extreme tilting of the bead leads to a small retroreflective surface area and minimal retroreflection, not ideal for robust tracking. (d) Bead placed with aluminum half-shell coated side up and glass side towards the coverslip. This configuration will have the worst retroreflection properties of all since minimum surface area is orthogonal to the tracking camera and capable of reflecting light back up to it. (e) Beads sprinkled on a microscope slide under a student stereomicroscope with side-lit fiber LED illumination. (f) When side-lit, beads in the correct orientation appear dark when viewed through the eyepiece (bead in red bounding box). Tilted beads appear brighter (bead in blue bounding box).

Supplementary Figure 3

a

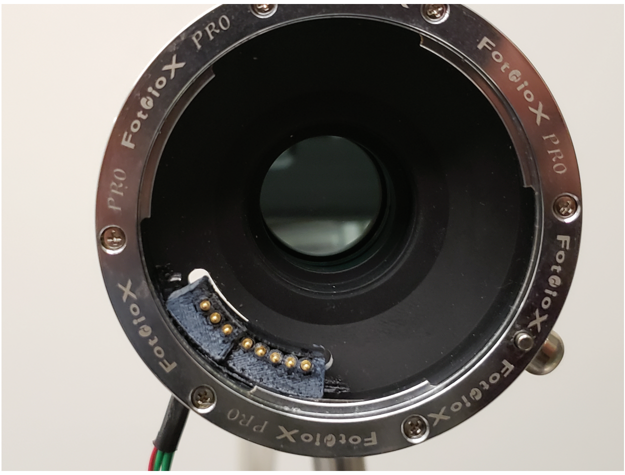

b

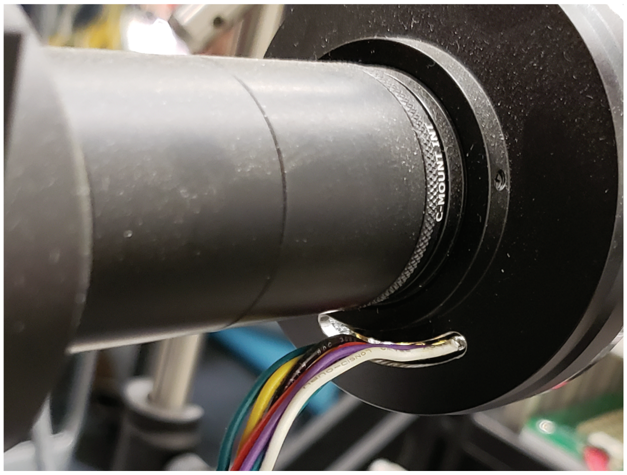

c

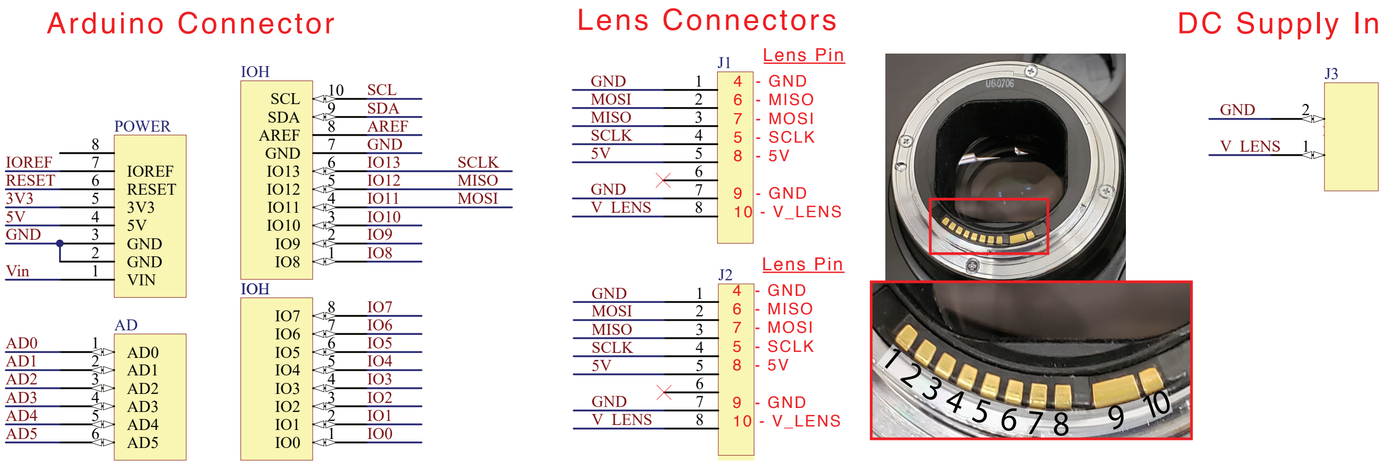

Supplementary Figure 3. Schematic of electronic lens control mechanism. (a) Front side of EF to C-mount lens adapter (to which fly- and fluo-view camera lenses are attached) with custom interface connector for driving lens via Arduino. (b) Backside of lens adapter shown in (a). (c) Left, labeled Arduino Uno shield pin connections. Middle, identical lens connector ports for fly-view and fluo-view camera lens control. Top image shows the backside of lens and pin interface used for electronic lens control. Bottom inset view shows labeled pin interface corresponding to the lens pin labels shown in the lens connector schematic. Right, input port for DC power supply.

Supplementary Figure 4

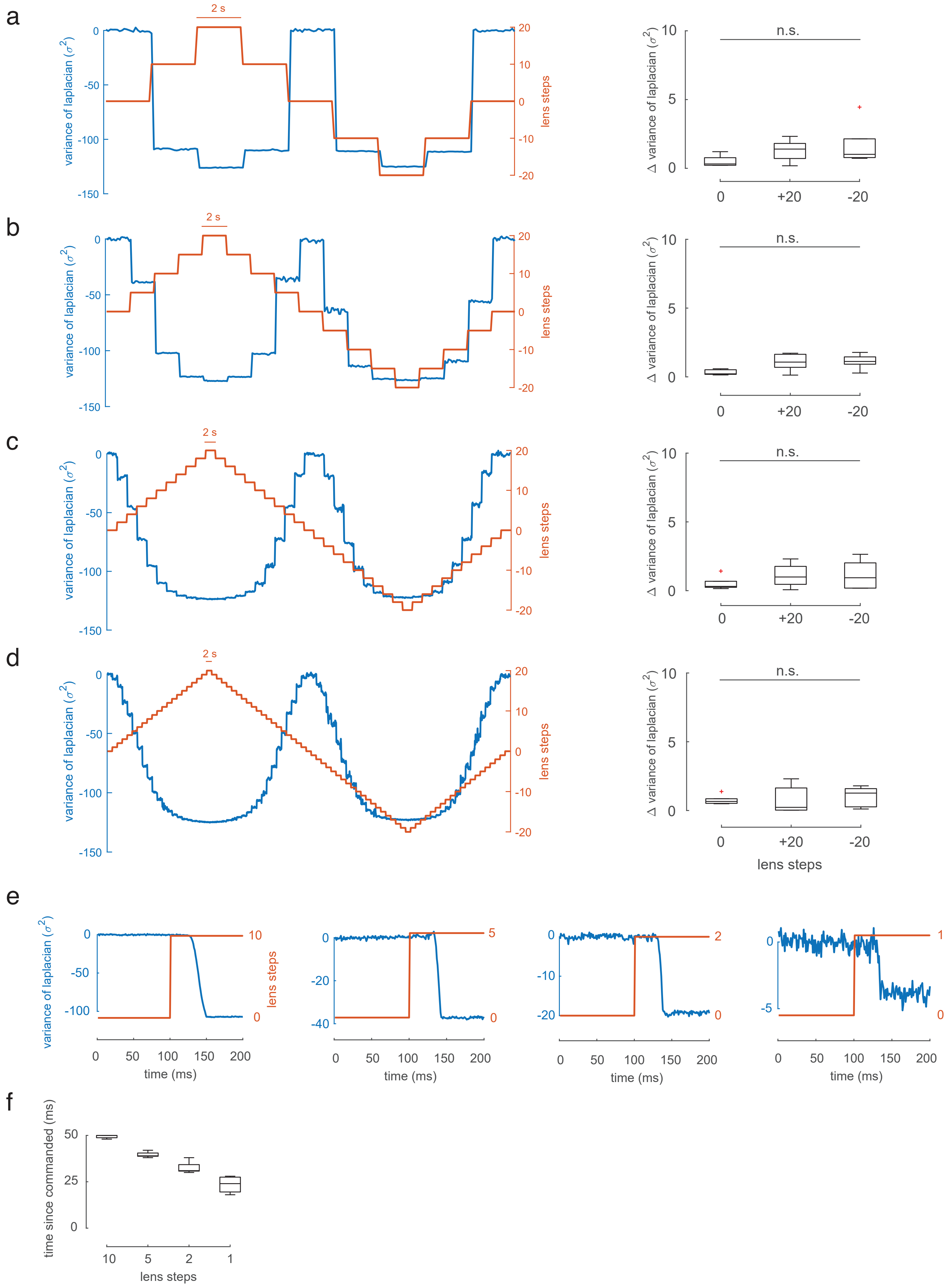

Supplementary Figure 4. Characterizing electronic lens focus repeatability and latency. Measuring focal plane repeatability by determining the variance of Laplacian of the image of an NBS 1952 Resolution Test Target captured with the fly-view camera at 1,000 Hz after moving the lens a total of 20 steps in the positive and negative directions. The test target was manually brought in focus at the start of each trial. Moves were performed in increments of (a) 10 steps, (b) 5 steps, (c) 2 steps, and (d) 1 step. Left, (a-d), in blue are the change in variance of Laplacian traces relative to the mean of the first 2 s at step intervals of 10, 5, 2, and 1 each. In red, are the lens step positions. Time between successive moves was 2 s. Right, (a-d), are boxplots indicating the change in variance of Laplacian for no moves (0 step), after 20 step moves in the positive direction, and 20 step moves in the negative direction, at step intervals of 10, 5, 2, and 1 each ($n = 6$). No significant difference was observed using a Mann Whitney-U test for change in variance of Laplacian values before and after each total 20-step move compared to the no move scenario. Change in variance of Laplacian was determined by the absolute difference of the first and second halves of the first 0-step interval (no move or 0-step), the second halves of the first 0-step interval and second 0-step interval (20 step move in positive direction), and the second halves of the second 0-step interval and third 0-step interval (20 step move in negative direction). (e, left-right) Change in variance of Laplacian (blue) for commanded moves of 10, 5, 2, and 1 steps (red) to indicate latency of completed move relative to commanded. (f) Latency of completed move relative to commanded time measured as the first time point when variance of Laplacian falls within 2σ of the mean of the second half of the time interval after the move ($n = 6$). Boxplot whiskers are 1.5x interquartile range.

Supplementary Figure 5

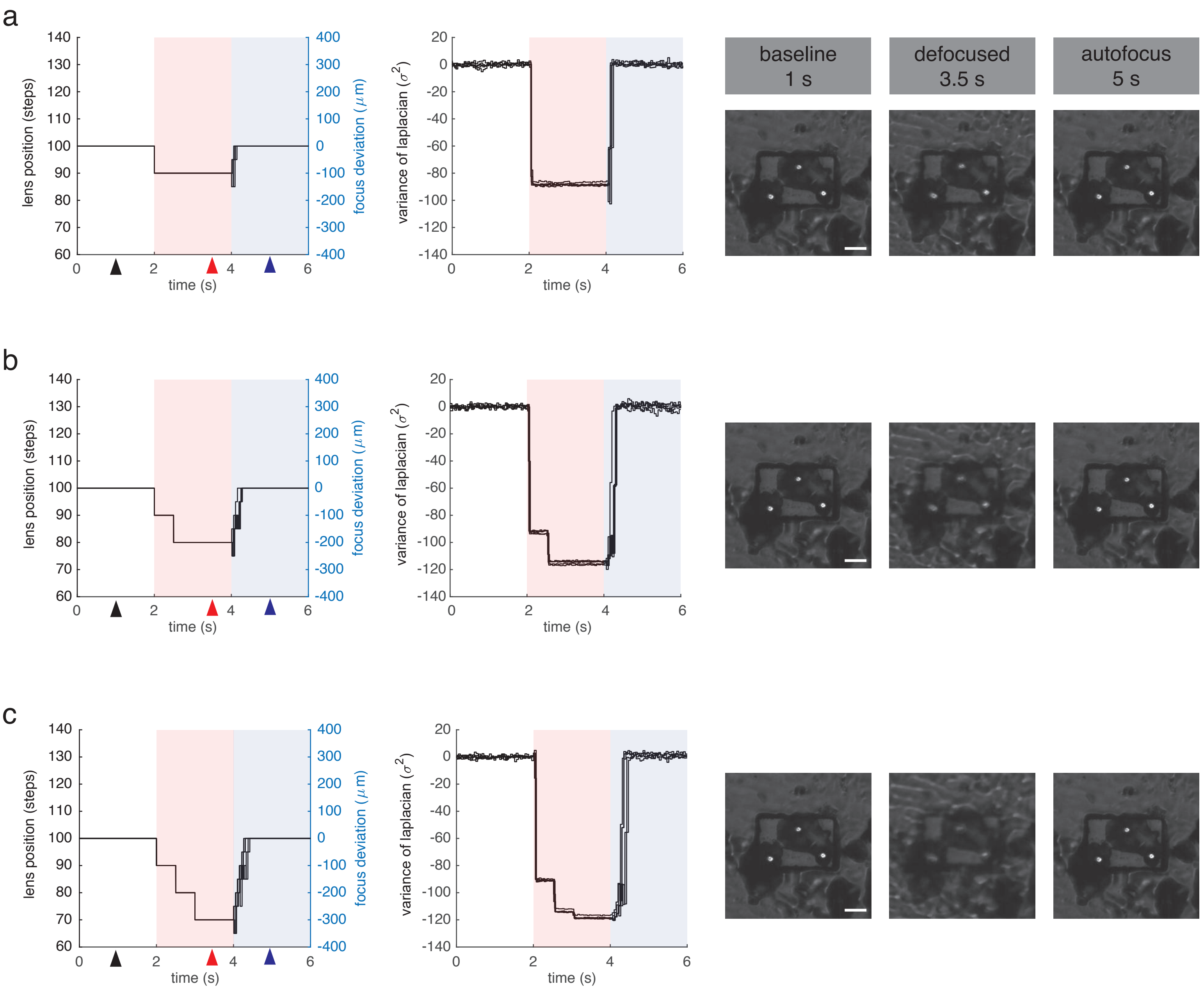

Supplementary Figure 5. Characterizing autofocus mechanism under negative z-plane displacement. Measuring the capability of the lens to autofocus after manual displacement of the lens by up to 30 steps (300 μm) in the negative z-plane direction. Lens movement and direction is determined to optimize and achieve a minimum difference of variance of Laplacian when compared to an *a priori* set baseline measure from a focused coverslip. Left, manual movement of the lens by (a) 10 steps, (b) 20 steps, and (c) 30 steps (red region), followed by initiation of autofocus (blue region) to move lens back to focal plane (n = 5). Center, time series of change of variance of Laplacian compared to baseline (first 2 s) corresponding to the lens positions in the left plot. Right, sample images of a focused coverslip (1 s, black arrowhead in left plot), manually defocused coverslip (3.5 s, red arrowhead in left plot), and after initiation of autofocus (5 s, blue arrowhead in left plot). Scale bars, 200 μm for fly-view camera images.

Supplementary Figure 6

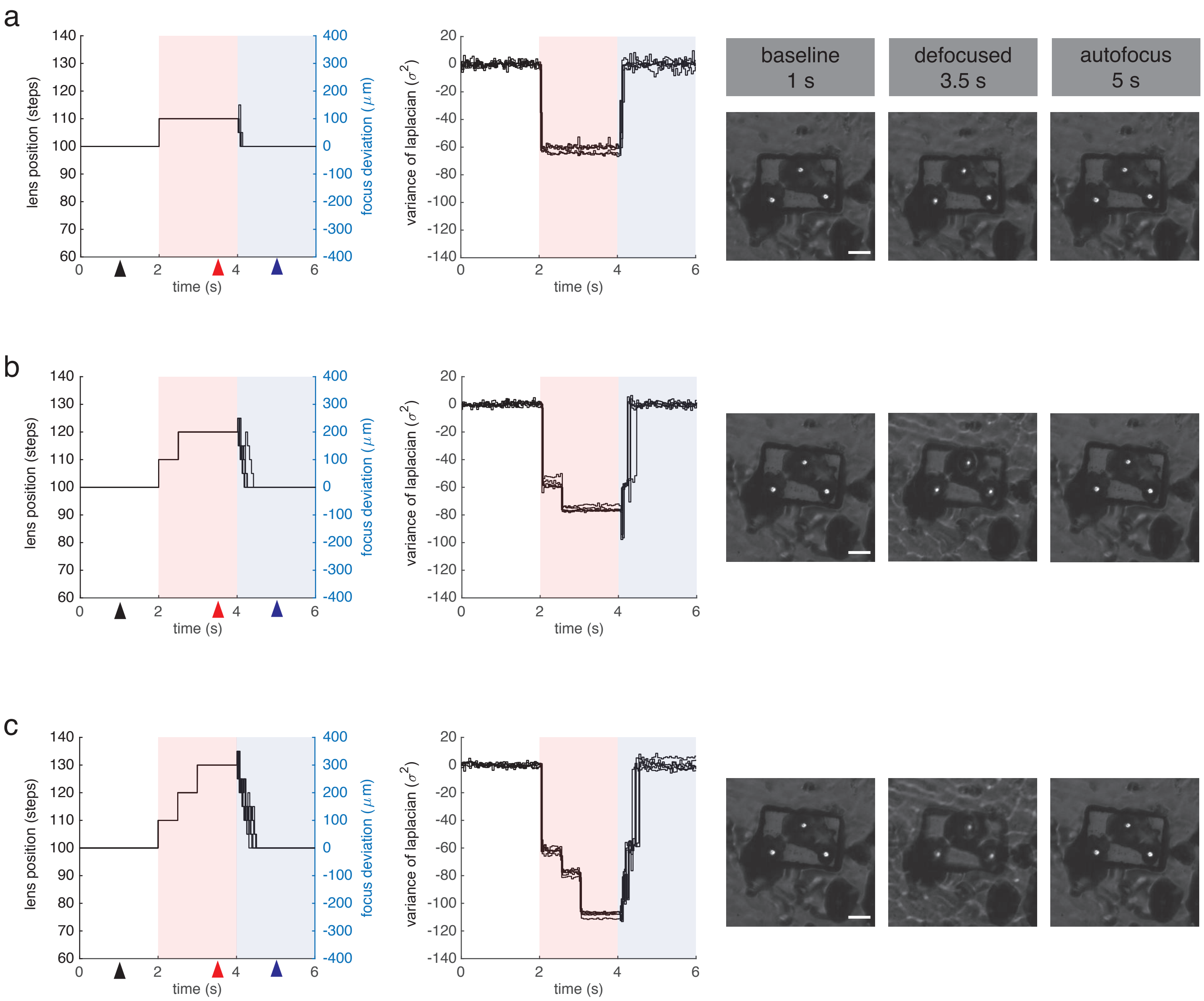

Supplementary Figure 6. Characterizing autofocus mechanism under positive z-plane displacement. Measuring the capability of the lens to autofocus after manual displacement of the lens by up to 30 steps (300 µm) in the positive z-plane direction. Lens movement and direction is determined to optimize and achieve a minimum difference of variance of Laplacian when compared to an *a priori* set baseline measure from a focused coverslip. Left, manual movement of the lens by (a) 10 steps, (b) 20 steps, and (c) 30 steps (red region), followed by initiation of autofocus (blue region) to move lens back to focal plane (n = 5). Center, time series of change of variance of Laplacian compared to baseline (first 2 s) corresponding to the lens positions in the left plot. Right, sample images of a focused coverslip (1 s, black arrowhead in left plot), manually defocused coverslip (3.5 s, red arrowhead in left plot), and after initiation of autofocus (5 s, blue arrowhead in left plot). Scale bars, 200 µm for fly-view camera images.

Supplementary Figure 7

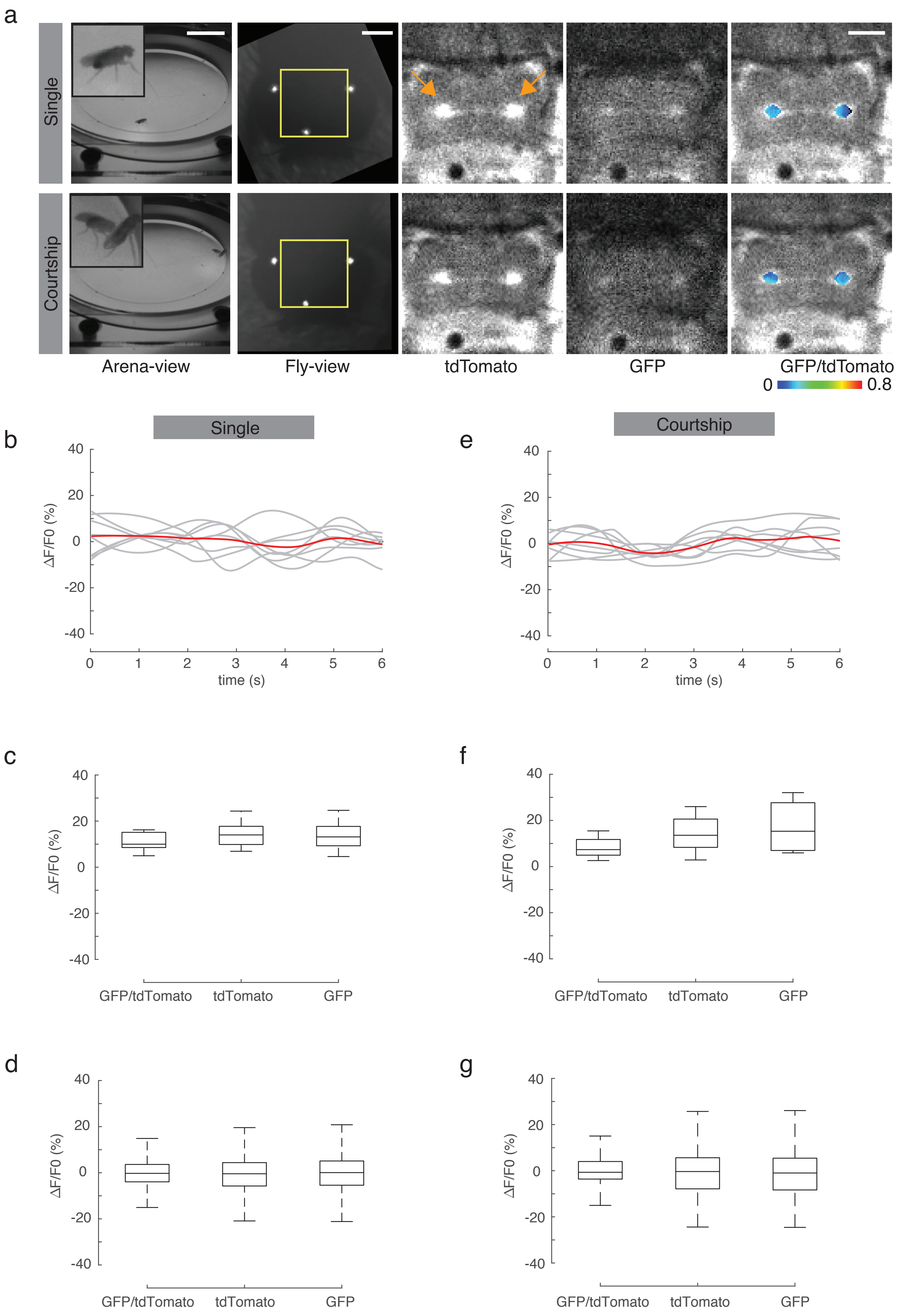

Supplementary Figure 7. Activity of calcium-independent ratio-metric imaging of fru-expressing P1 neurons during naturally evoked courtship. (a) Top row, a free walking virgin male fly with *P1a-Gal4*, *UAS-CD8-GFP*, and *UAS-myr-tdTomato* transgenes. Bottom row, the same male fly naturally courting a female fly. From left to right; arena-view with an inset showing a zoomed-in view of the flies, fly-view, fluo-view tdTomato channel, fluo-view GFP channel, and fluo-view tdTomato channel with a pseudo-color $F_{ratio} = F_{GFP}/F_{tdTomato}$ representation of the GFP signal in P1 neurons. Yellow boxes in the fly-view images indicate the areas shown in the fluo-view. Orange arrows indicate the locations of P1 neurons. (b) Time series of dF_{ratio}/F_{ratio} values showing LOESS (local polynomial regression) curve fits (gray) from free walking naïve males (n = 6), with mean activity curve (red). (c) Comparison of mean GFP/tdTomato ratio-metric values with tdTomato and GFP values independently, measured as a percent difference of maximum F_{ratio} values from overall mean. (d) Comparison of mean GFP/tdTomato ratio-metric values with tdTomato and GFP values independently, measured as a percent difference of individual F_{ratio} values from overall mean. (e) Time series of dF_{ratio}/F_{ratio} values showing LOESS curve fits (gray) from free walking male flies during naturally-evoked courtship of a female (n = 6), with mean activity curve (red). (f) Comparison of mean GFP/tdTomato ratio-metric values with tdTomato and GFP values independently, during natural courtship sequences measured as a percent difference of maximum F_{ratio} values from overall mean. (g) Comparison of mean GFP/tdTomato ratiometric values with tdTomato and GFP values during courtship sequences measured as a percent difference of individual F_{ratio} values from overall mean. Scale bars, 10 mm for arena-view, 200 μm for fly-view, and 100 μm for fluo-view. Boxplot whiskers are 1.5x interquartile range.

Supplementary Figure 8

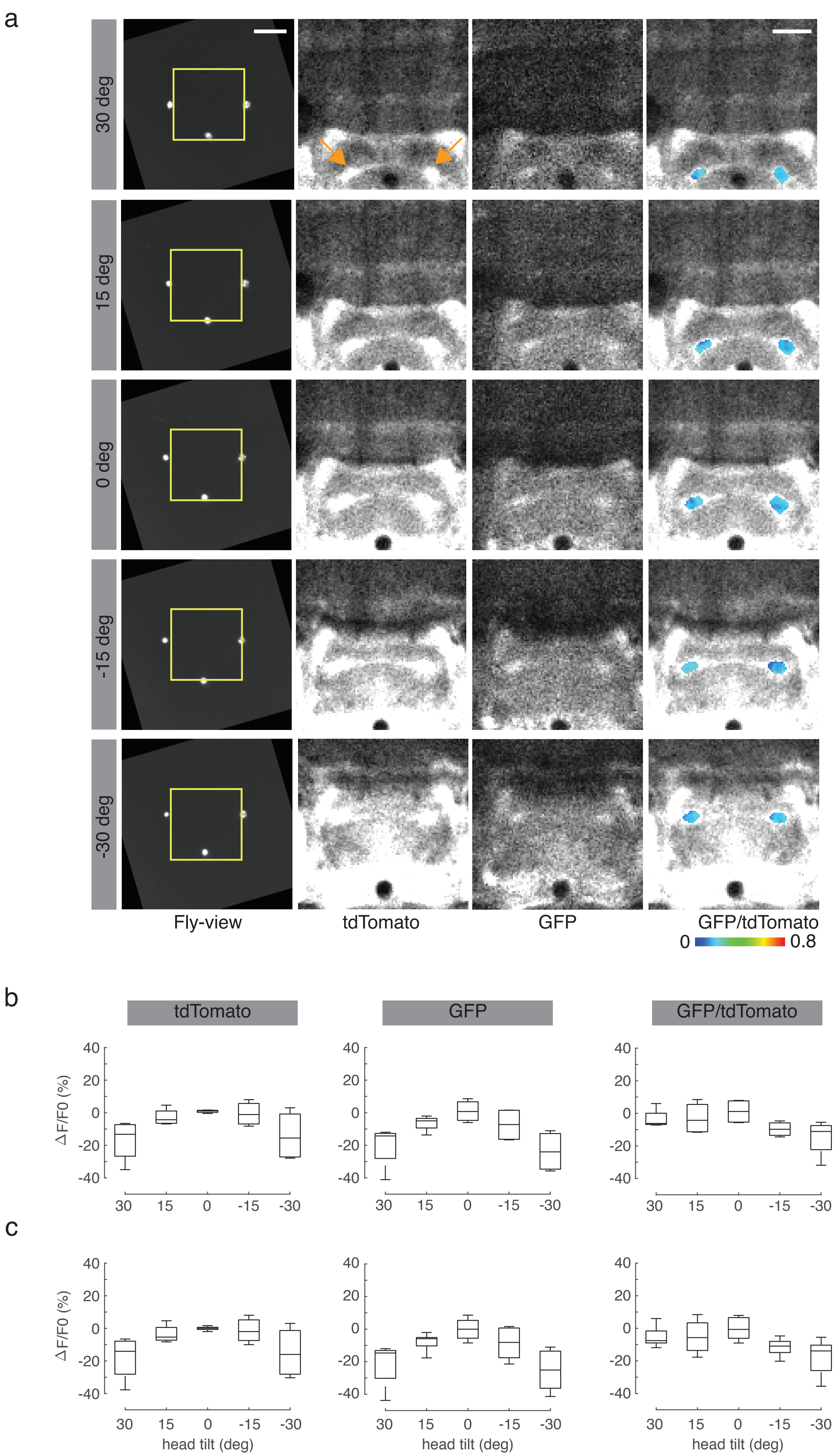

Supplementary Figure 8. Profiling signal stability of calcium-independent ratio-metric imaging across different head tilt pitch angles in tethered flies. (a) A tethered virgin male fly with *P1a-Gal4*, *UAS-CD8-GFP*, and *UAS-myr-tdTomato* transgenes positioned in the center of the fly- and fluo-view camera fields-of-view with galvanometer mirrors centered, and at varied head tilt pitch angles with height compensation for change in pitch. From top-bottom: head tilt pitch angles of 30, 15, 0 (flat), -15, and -30 degrees respectively. From left to right; fly-view, fluo-view tdTomato channel, fluo-view GFP channel, and fluo-view tdTomato channel with a pseudo-color $F_{ratio} = F_{GFP}/F_{tdTomato}$ representation of the GFP signal in P1 neurons. Yellow boxes in the fly-view images indicate the areas shown in the fluo-view. Orange arrows indicate the locations of P1 neurons. (b) Comparison of mean independent tdTomato (left), GFP (middle), and GFP/tdTomato ratio-metric (right) values measured as a percent difference of maximum F_{ratio} values from baseline mean of 0-degree tilt condition. (c) Comparison of mean independent tdTomato (left), GFP (middle), and GFP/tdTomato ratio-metric (right) values measured as a percent difference of individual F_{ratio} values from mean of 0-degree tilt condition. The experiment length for each head tilt pitch angle was 15 sec (n = 4). Scale bars, 200 μm for fly-view, and 100 μm for fluo-view. Boxplot whiskers are 1.5x interquartile range.

Supplementary Figure 9

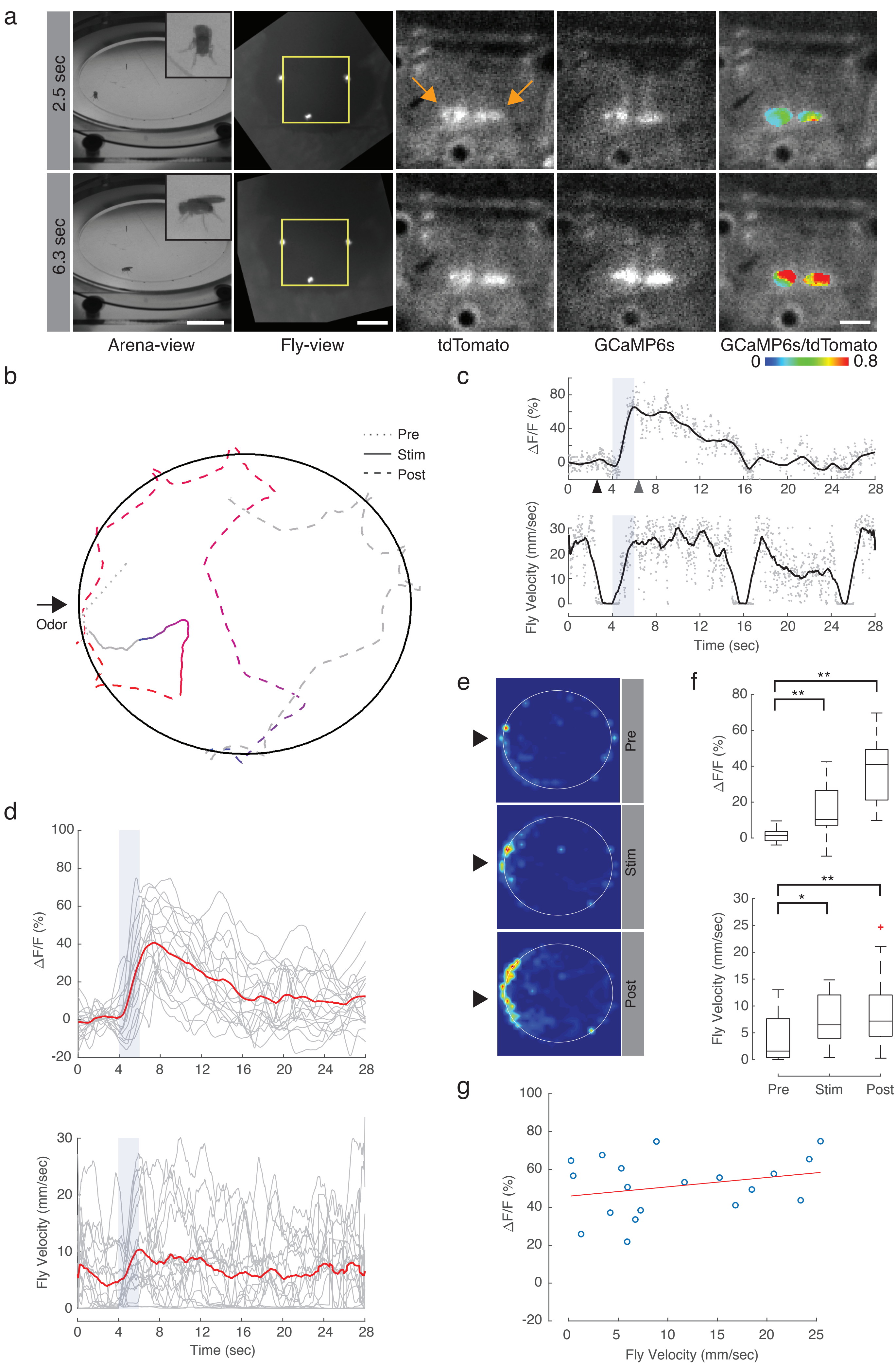

Supplementary Figure 9. Activity of olfactory projection neurons to odor pulses in free walking flies. (a) Top row, a free walking male fly with *GH146-Gal4*, *UAS-GCaMP6s,* and *UAS-myr-tdTomato* transgenes. Bottom row, the same male fly shortly after a 2 sec pulse of ethanol odorant in the arena. From left to right; arena-view with an inset showing a zoomed-in view of the fly, fly-view, fluo-view tdTomato channel, fluo-view GCaMP6s channel, and fluo-view tdTomato channel with a pseudo-color $F_{ratio} = F_{GCaMP6s}/F_{tdTomato}$ representation of the GCaMP6s signal in *GH146* olfactory projection neurons. Yellow boxes in the fly-view images indicate the areas shown in the fluo-view. Orange arrows indicate the locations of olfactory projection neurons. (b) 2D trajectory in real-world coordinates of the fly in (a) walking in the behavior arena (major axis - 44 mm, minor axis - 40 mm, black circle outline). Trajectory length ranges from 2 sec before odor stimulus onset until 10 sec after stimulus termination. Dotted line segment indicates pre-stimulus trajectory, solid line is during stimulus presentation, and dashed line is trajectory segment post-stimulus. Trajectory color code indicates 40% to max dF_{ratio}/F_{ratio} values of the trial in (a) from blue to red. The black arrowhead indicates the location of the odor port relative to fly arena. (c) Top row, time series of dF_{ratio}/F_{ratio} raw values (gray) and LOESS (local polynomial regression) curve fit (black) from the male in (a). Bottom row, time series of fly velocity raw values (gray) and LOESS curve fit (black) from the male in top row and (a). Gray bars indicate 2 s time duration of odor presentation. The black and gray arrowheads indicate the time points at which images in (a), top and bottom panels respectively, were captured. (d) Top row, time series of dF_{ratio}/F_{ratio} values showing LOESS curve fits (gray), with mean curve (red) from multiple odor presentation trials (n = 19). Bottom row, time series of fly velocity values showing LOESS curve fits (gray), with mean curve (red) from trials in top row of (d). Gray bars indicate time 2 s time duration of odor presentation. (e) 2D histogram (2 mm bins in x- and y-axis) position color heatmaps of aggregate fly position in the arena across trials shown in (d) (n = 19), during 2 s interval prior to odor stimulus onset (Pre, top row), during 2s odor stimulus presentation (Stim, middle row), and during 2 s post-stimulus termination (Post, bottom row). Black arrowheads indicate location of odor port relative to fly arena. White circle outline represents boundary of fly arena. (f) Top row, boxplot of mean dF_{ratio}/F_{ratio} values per trial corresponding to (d) (n = 19), for 2 s pre-, stim- and post-odor time intervals corresponding to (e). Bottom row, boxplot of mean fly velocities per trial corresponding to the top row and (d), for 2 s pre-, stim- and post-odor time intervals. (g) Peak dF_{ratio}/F_{ratio} values as a function of different fly velocities for data presented in (d), no significant correlation with coefficient 0.19, $p >> 0.01$. Red line indicates linear polynomial curve fit of data points. Boxplot whiskers are 1.5x interquartile range.

Supplementary Figure 10

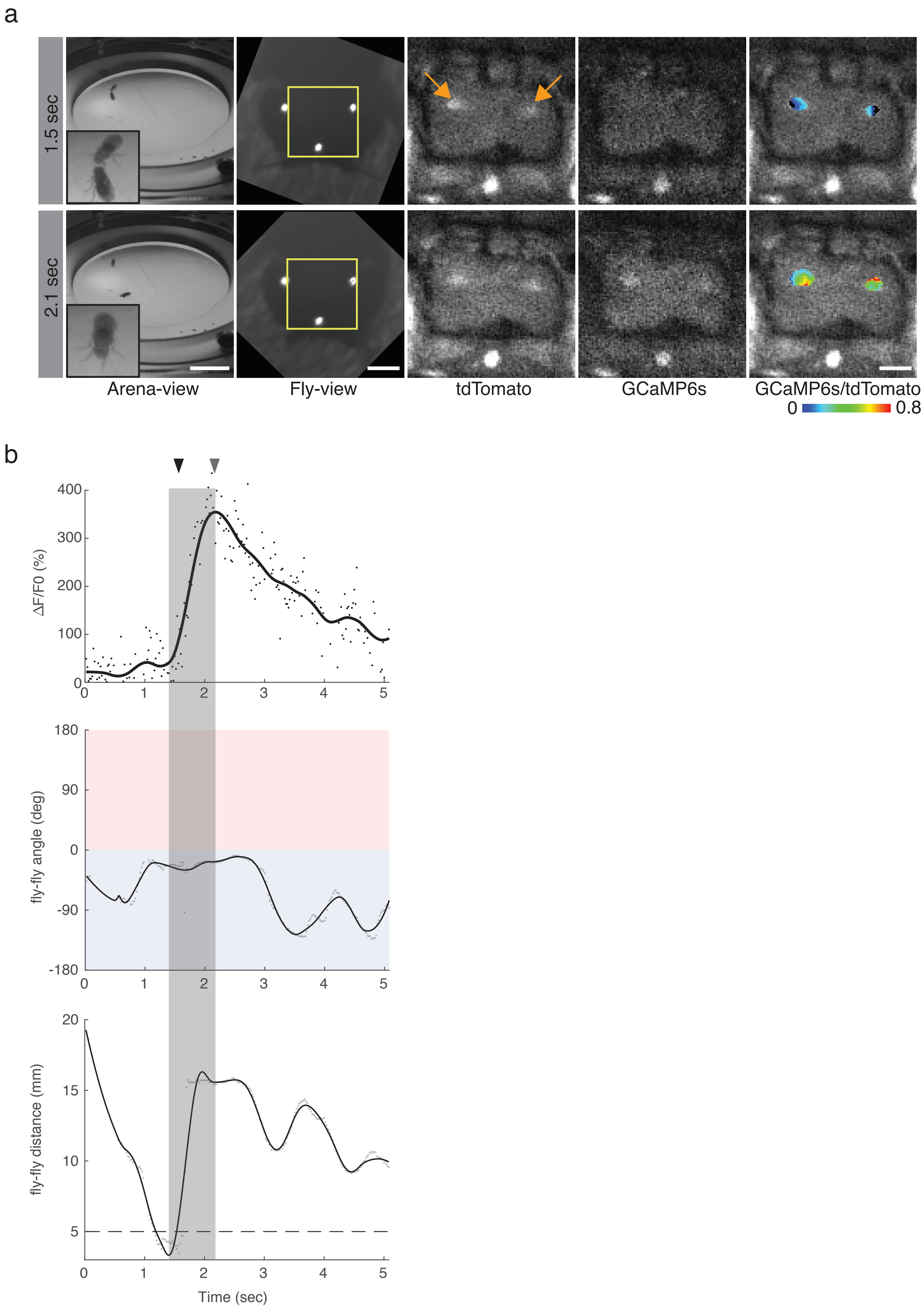

Supplementary Figure 10. Activity of fru-expressing P1 neurons during an interaction after copulation. (a) The same male fly as in Figure 2 interacting with the female fly after mating. Top and bottom row show frames without and with activity in P1 neurons, respectively. From left to right; arena-view with an inset showing a zoomed-in view of the flies, fly-view, fluo-view tdTomato channel, fluo-view GCaMP6s channel, and fluo-view tdTomato channel with a pseudo-color $F_{ratio} = F_{GCaMP6s}/F_{tdTomato}$ representation of the GCaMP6s signal in P1 neurons. Yellow boxes in the fly-view images indicate the areas shown in the fluo-view. Orange arrows indicate the locations of P1 neurons. (b) Top row, time series of dF_{ratio}/F_{ratio} raw values (black scatter), with corresponding LOESS (local polynomial regression) curve fit (black) from the data of the interacting male in (a). The black and gray arrowheads indicate the time points at which the images in (a) top and bottom row were captured, respectively. Middle row, time series of raw angular difference of male fly's heading relative to female fly (gray) with LOESS curve fit (black). A positive angular difference (red region) indicates the female fly is on the right-hand side of the male, and a negative angular difference (blue region) indicates the female fly is on the left-hand side of the male. Bottom row, time series of raw euclidean distance between tracked centroid positions of the male and female flies (gray) with LOESS curve fit (black). Dashed line at 5 mm indicates upper distance threshold for interaction and contact events. Gray bar passing through top and bottom rows indicates time interval between the instance when the two flies are at their closest distance from each other (below 5mm, bottom row), and peak P1 dF_{ratio}/F_{ratio} activity (top row), determined to be 0.78 s. Scale bars, 10 mm for arena-view, 200 μm for fly-view, and 100 μm for fluo-view.

Supplementary Figure 11

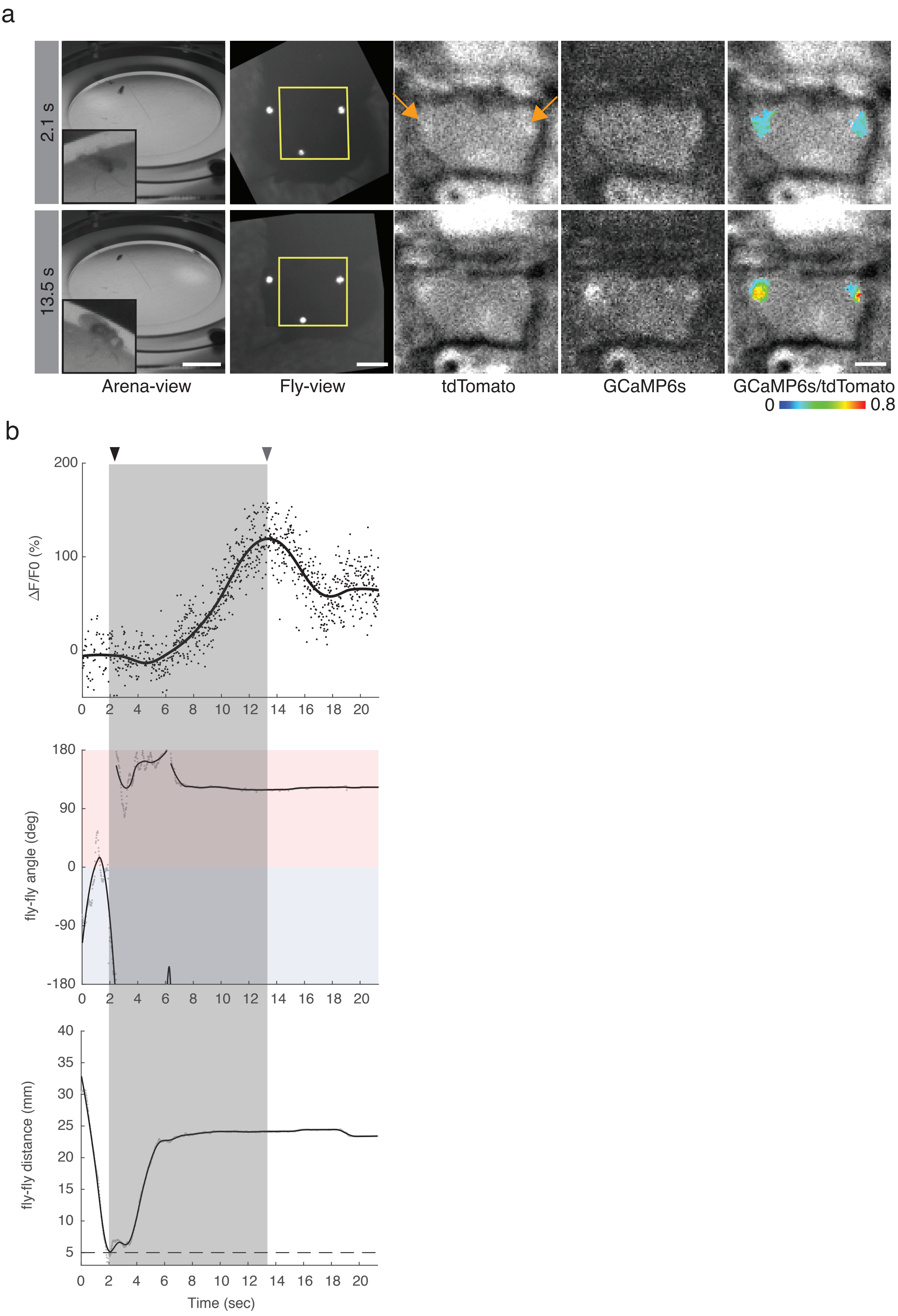

Supplementary Figure 11. Activity of GABAergic mAL neurons during an interaction after copulation. (a) The same male fly as in Figure 3 interacting with the female fly after mating. Top and bottom row show frames without and with activity in mAL neurons, respectively. From left to right; arena-view with an inset showing a zoomed-in view of the flies, fly-view, fluo-view tdTomato channel, fluo-view GCaMP6s channel, and fluo-view tdTomato channel with a pseudo-color $F_{ratio} = F_{GCaMP6s}/F_{tdTomato}$ representation of the GCaMP6s signal in mAL neurons. Yellow boxes in the fly-view images indicate the areas shown in the fluo-view. Orange arrows indicate the locations of mAL neurons. (b) Top row, time series of dF_{ratio}/F_{ratio} raw values (black scatter), with corresponding LOESS (local polynomial regression) curve fit (black) from the data of the interacting male in (a). The black and gray arrowheads indicate the time points at which the images in (a) top and bottom row were captured, respectively. Middle row, time series of raw angular difference of male fly's heading relative to female fly (gray) with LOESS curve fit (black). A positive angular difference (red region) indicates the female fly is on the right-hand side of the male, and a negative angular difference (blue region) indicates the female fly is on the left-hand side of the male. Bottom row, time series of raw euclidean distance between tracked centroid positions of the male and female flies (gray) with LOESS curve fit (black). Dashed line at 5 mm indicates upper distance threshold for interaction and contact events. Gray bar passing through top and bottom rows indicates time interval between the instance when the two flies are at their closest distance from each other (below 5mm, bottom row), and peak P1 dF_{ratio}/F_{ratio} activity (top row), determined to be 11.3 s. Scale bars, 10 mm for arena-view, 200 μm for fly-view, and 100 μm for fluo-view.

Supplementary Figure 12

a

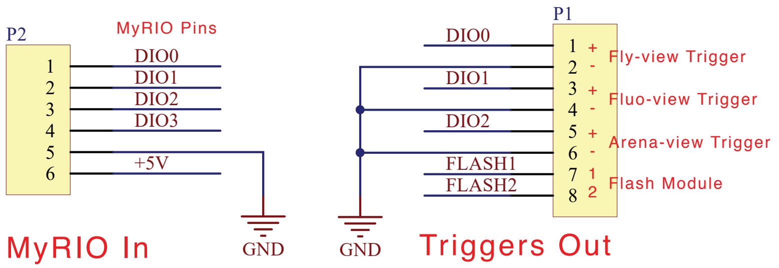

b

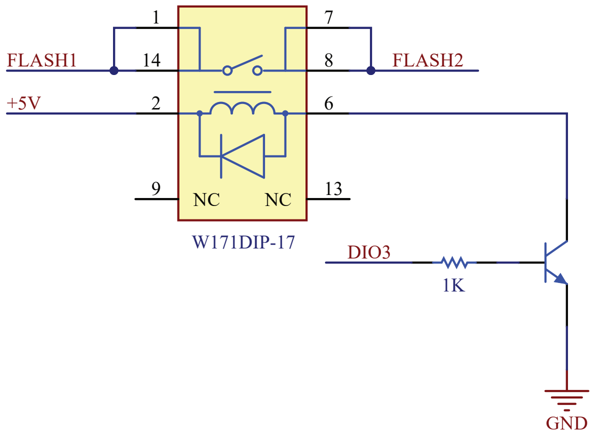

Supplementary Figure 12. Schematic of camera triggering and flash circuitry. (a) Left, input connection port from National Instruments myRIO embedded device labeled corresponding to its output lines. Right, output port to camera GPIO trigger pins and flash module with corresponding labels. (b) Schematic of flash module that takes as input a digital signal from the myRIO and switches a read relay to fire the flash speedlite.

Supplementary Figure 13

a

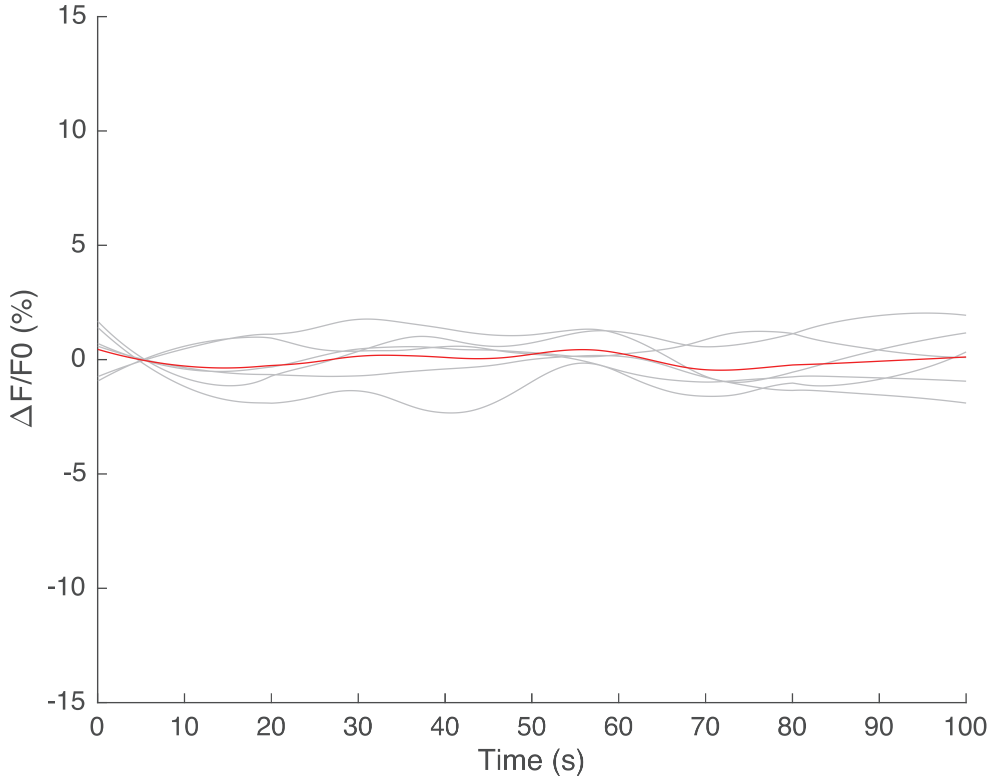

b

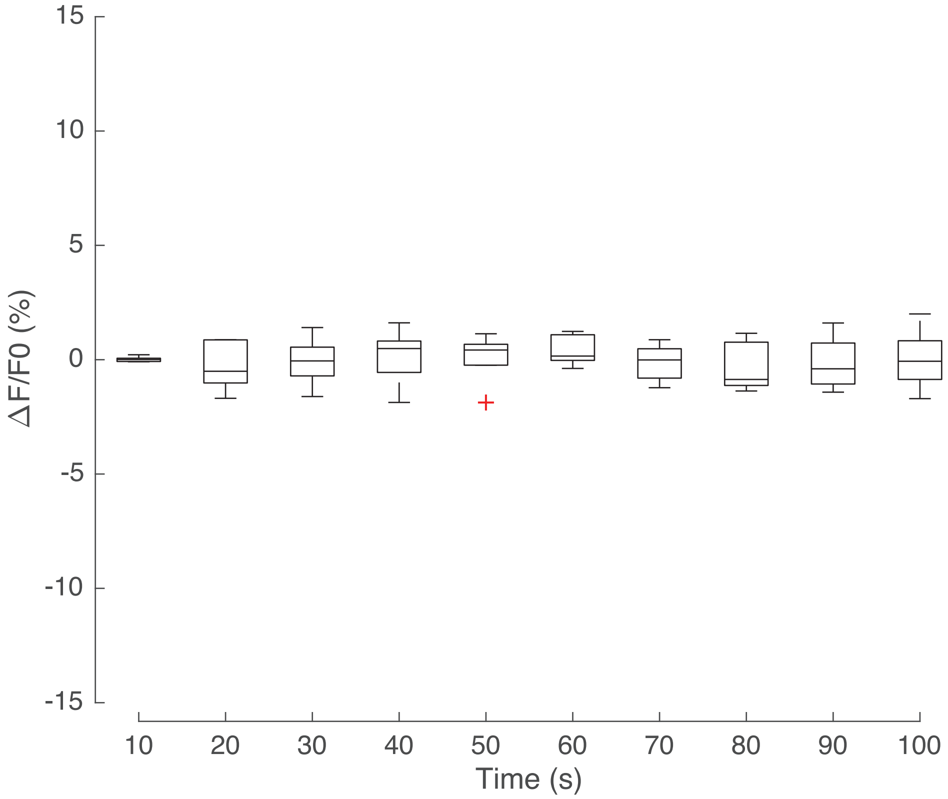

Supplementary Figure 13. Bleaching profile of tdTomato fluorescence signal in fru-expressing P1 neurons from a tethered male fly. (a) Time series of dF_{ratio}/F_{ratio} values showing individual trial LOESS (local polynomial regression) curve fits (gray) with mean curve (red). F_{ratio} is determined by normalizing each point in the time series to baseline mean of the first 10 s. (n = 6). (b) dF_{ratio}/F_{ratio} values binned in intervals of 10 s and normalized to the first 10 s bin (n = 6). Boxplot whiskers are 1.5x interquartile range.

Supplementary Figure 14

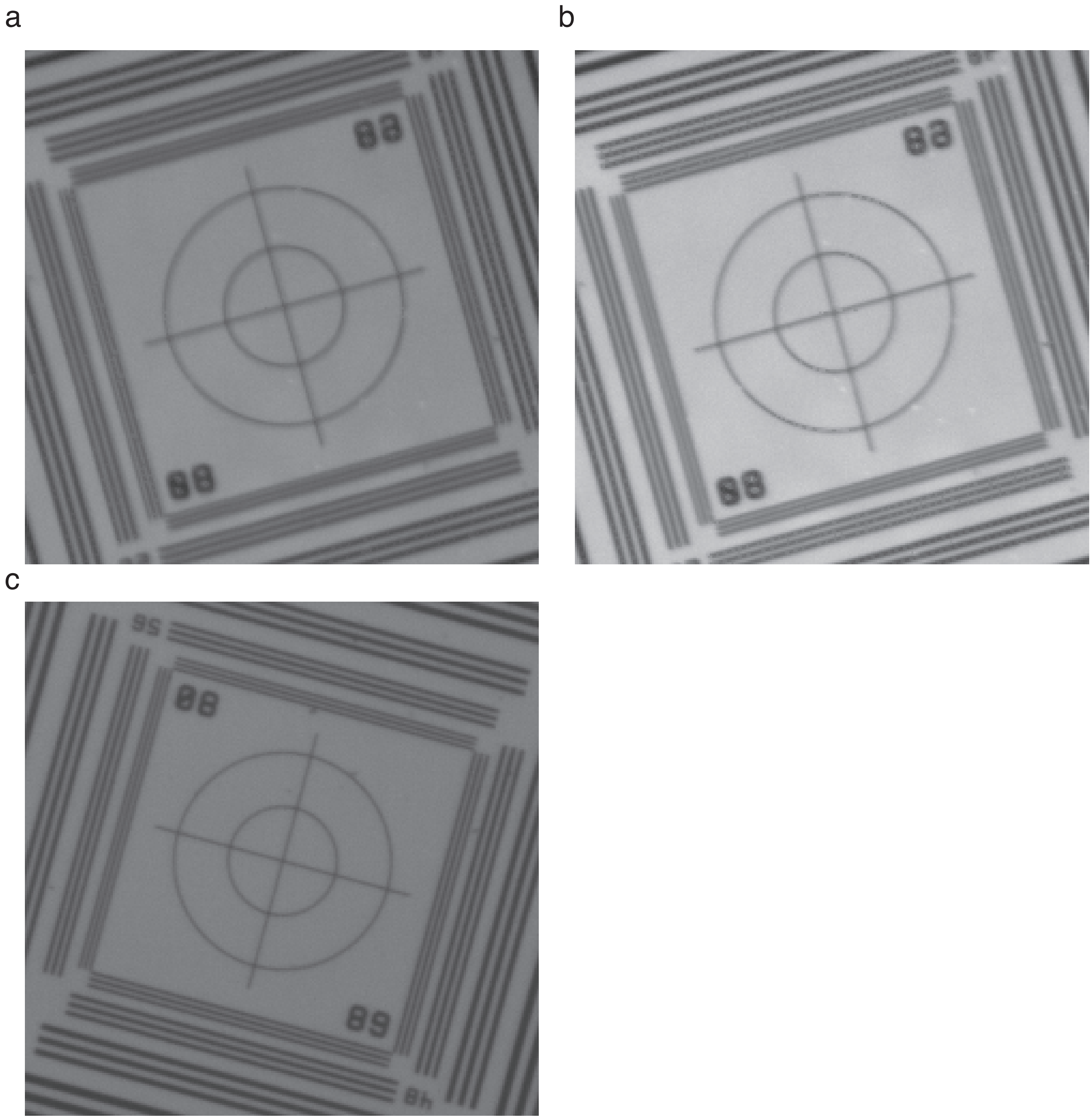

Supplementary Figure 14. Resolution test. Image of NBS 1952 Resolution Test Target captured with fluo-view camera green (a) and red (b) channels, and fly-view camera (c). Numbers in the image indicate line pairs per millimeter ($lpmm^{-1}$). A Crosshair is 610 μm in length and width and two concentric circles are 250 μm and 500 μm in diameter.

Supplementary Figure 15

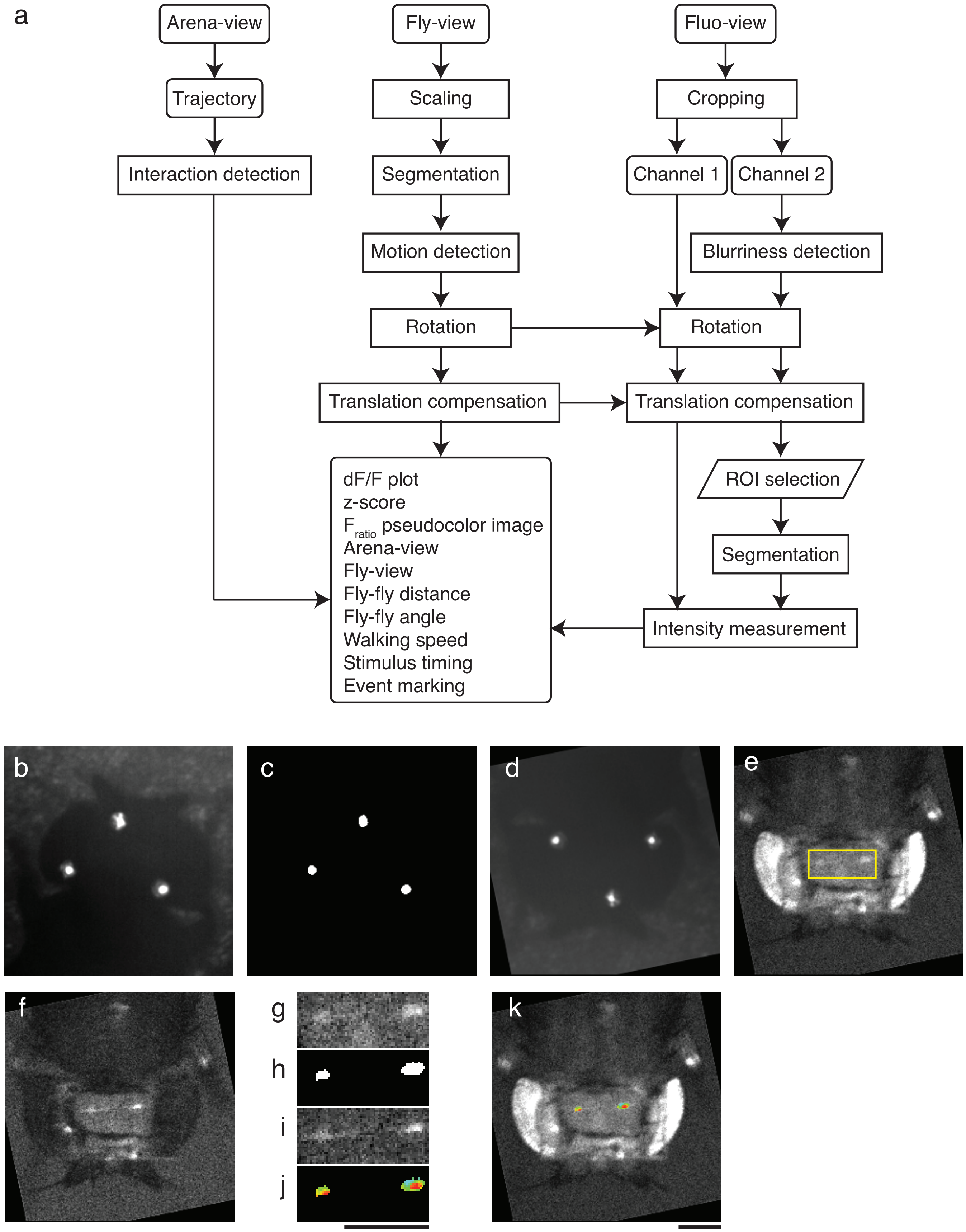

Supplementary Figure 15. Post-acquisition image processing pipeline. (a) Components and flow of Flyception2 image analysis pipeline. (b) Raw fly-view image. (c) A binary image of (b) showing the segmented retroreflective beads after adaptive thresholding. (d) Result of fast normalized cross correlation used for template matching. (e) Fly-view image after applying rotation and translation compensation. (f and g) Fluo-view red and green channels, respectively, after applying the same rotation and translation compensation as for fly-view images. (h) Interactively-selected ROI for segmenting P1 neurons in the tdTomato (red) channel. (i) P1 neurons after segmentation. (j) GCaMP signals in the same ROI as in (h). (k) GCaMP6s/tdTomato ratio-metric pseudo-color representation. (l) An overlaid image of fluo-view tdTomato channel and GCaMP6s/tdTomato ratio-metric pseudo-color representation. Scale bars, 200 μm.

Females	*Canton S*
P1a>>GCaMP6s-tdTomato Males	*w*; *P1a-Gal4 AD/UAS-20XGCaMP6s, UAS-myr-tdTomato; P1a-Gal4 DBD/Sb*
mAL>>GCaMP6s-tdTomato Males	*w*; *UAS-20XGCaMP6s, UAS-myr-tdTomato/+*; *R25E04-Gal4^{attP2}/Sb*
GH-146>>GCaMP6s-tdTomato Males	*w; GH146-Gal4/UAS-20XGCaMP6s, UAS-myr-tdTomato; Sb/+*
P1a>>GFP-tdTomato Males	*w; P1a-Gal4 AD/UAS-CD8-GFP; P1a-Gal4 DBD/UAS-tdTomato*

Supplementary Table 1. Detailed fly genotypes.

Isolation table

1	Thorlabs	PTA512	Air Compressor -110/115 V - 60 Hz, US Power Plug
1	Thorlabs	PFA52507	800 W/FRAME 1200x900 ACTIVE
1	Thorlabs	B3648G	36" x 48" x 4.3" Imperial Breadboard

Galvanometer assembly

1	Thorlabs	GVS012	2D Large Beam (10 mm) Diameter Galvo System, Silver-Coated Mirrors
1	Thorlabs	GPS011	1D or 2D Galvo System Linear Power Supply
3	Thorlabs	AP90	Right-Angle Mounting Plate, 1/4"-20 Compatible
1	Thorlabs	MB12	Aluminum Breadboard 12" x 12" x 1/2", 1/4"-20 Taps

Fly arena

4	Thorlabs	TR3	Ø1/2" Optical Post, SS, 8-32 Setscrew, 1/4"-20 Tap, L = 3"
4	Thorlabs	TR1	Ø1/2" Optical Post, SS, 8-32 Setscrew, 1/4"-20 Tap, L = 1"
4	Thorlabs	TR075	Ø1/2" Optical Post, SS, 8-32 Setscrew, 1/4"-20 Tap, L = 0.75"
1	Thorlabs	MB4	Aluminum Breadboard 4" x 6" x 1/2", 1/4"-20 Taps
1	Newport	38	Mounting Platform, 3 in. x 5 in. x 0.5 in., 1/4-20 Thread
1	Newport	562F-XYZ-LH	ULTRAlign Fiber Alignment Stage, Left-Handed, 13 mm XYZ Travel, 8-32 and 1/4-20
3	Newport	HR-13	High Resolution Micrometer, 0.5 µm Sensitivity, 13 mm Travel
1	BK Precision	1698	Programmable DC switching power supply, 0-60V, 0-3.3A

Arena view camera

1	Thorlabs	TR8	Ø1/2" Optical Post, SS, 8-32 Setscrew, 1/4"-20 Tap, L = 8"
1	Thorlabs	TR3	Ø1/2" Optical Post, SS, 8-32 Setscrew, 1/4"-20 Tap, L = 3"
1	Thorlabs	SWC	Rotating Clamp for Ø1/2" Posts, 360° Continuously Adjustable, 3/16" Hex
1	Thorlabs	PH3	Ø1/2" Post Holder, Spring-Loaded Hex-Locking Thumbscrew, L = 3"
1	Point Grey	FL3-U3-13Y3M-C	Flea3 USB3.0 camera
1	Computar	M0814-MP2	8mm 2/3" fixed focal lens
1	Edmund Optics	84746	Filter longpass OD4 - 625nm 25mm

1	Edmund Optics	65801	M30.5 mount for 25mm filters
1	Thorlabs	CF125	Clamping Fork, 1.24" Counterbored Slot, Universal
1	Thorlabs	PH4E	Ø1/2" Pedestal Post Holder, Spring-Loaded Hex-Locking Thumbscrew, L=6.19"

Fly view camera

2	Thorlabs	PH2	Ø1/2" Post Holder, Spring-Loaded Hex-Locking Thumbscrew, L = 2"
1	Thorlabs	TR2	Ø1/2" Optical Post, SS, 8-32 Setscrew, 1/4"-20 Tap, L = 2"
1	Thorlabs	TR4	Ø1/2" Optical Post, SS, 8-32 Setscrew, 1/4"-20 Tap, L = 4"
1	Thorlabs	SM1A9	Adapter with External C-Mount Threads and Internal SM1 Threads
1	Thorlabs	SM1A10	Adapter with External SM1 Threads and Internal C-Mount Threads
1	Thorlabs	SM1L03	SM1 Lens Tube, 0.3" Thread Depth
1	Fotodiox	EOS-C Pro	lens mount adapter, Canon EOS EF/EF-s mount to C-mount
1	Edmund Optics	84746	Filter longpass OD4 - 625nm 25mm
1	Point Grey	GZL-CL-22C5M-C	Gazelle camera link camera
1	Point Grey	ACC-01-9009	Camera Link power supply
2	Point Grey	ACC-01-2200	Camera Link cables
1	Point Grey	ACC-01-3000	1.0 meter, Circular 8-pin pre-wired GPIO Hirose Connector
2	Newport	PRC-1	Rail Carrier, 1.0 in. Length, 1/4-20 Thread, PRL Series
1	Newport	PRL-12	Precision Optical Rail, 12.46 in. Length, 3.93 in. Width, 12 in. Scale
3	Newport	HR-13	High Resolution Micrometer, 0.5 µm Sensitivity, 13 mm Travel
1	Newport	562-XYZ	ULTRAlign Precision XYZ Linear Stage, 13 mm Travel, Req. 3 Actuators
1	Canon	2539A007AA	EF 180mm f/3.5L Macro USM

Fluo view camera

3	Thorlabs	PH2	Ø1/2" Post Holder, Spring-Loaded Hex-Locking Thumbscrew, L = 2"
1	Thorlabs	PH1	Ø1/2" Post Holder, Spring-Loaded Hex-Locking Thumbscrew, L = 1"

2	Thorlabs	TR2	Ø1/2" Optical Post, SS, 8-32 Setscrew, 1/4"-20 Tap, L = 2"
1	Thorlabs	TR1.5	Ø1/2" Optical Post, SS, 8-32 Setscrew, 1/4"-20 Tap, L = 1.5"
1	Thorlabs	TR1	Ø1/2" Optical Post, SS, 8-32 Setscrew, 1/4"-20 Tap, L = 1"
2	Thorlabs	TR075	Ø1/2" Optical Post, SS, 8-32 Setscrew, 1/4"-20 Tap, L = 0.75"
1	Thorlabs	BA2	Mounting Base, 2" x 3" x 3/8"
1	Thorlabs	SM1A9	Adapter with External C-Mount Threads and Internal SM1 Threads
1	Thorlabs	SM1A10	Adapter with External SM1 Threads and Internal C-Mount Threads
1	Thorlabs	SM1L20	SM1 Lens Tube, 2.0" Thread Depth
1	Fotodiox	EOS-C Pro	lens mount adapter, Canon EOS EF/EF-s mount to C-mount
1	Semrock	FF01-520/35-25	520/35 nm BrightLine single-band bandpass filter
1	Semrock	FF01-607/36-25	607/36 nm BrightLine single-band bandpass filter
1	Semrock	FF580-FDi01-25x36	580 nm edge BrightLine® single-edge imaging-flat dichroic beamsplitter
4	Newport	PRC-1	Rail Carrier, 1.0 in. Length, 1/4-20 Thread, PRL Series
1	Newport	PRL-24	Precision Optical Rail, 24.28 in. Length, 3.93 in. Width, 24 in. Scale
2	Newport	HR-13	High Resolution Micrometer, 0.5 µm Sensitivity, 13 mm Travel
1	Newport	SM-25	Vernier Micrometer, 25 mm Travel, 23 lb Load Capacity, 50.8 TPI
1	Newport	562-XYZ	ULTRAlign Precision XYZ Linear Stage, 13 mm Travel, Req. 3 Actuators
1	HAMAMATSU	A12801-01	W-VIEW GEMINI OPTICAL SPLITTER, INCL HOLDER
1	Photometrics	01-PRIME-R-M-16-C	PRIME MONO
1	Canon	2539A007AA	EF 180mm f/3.5L Macro USM

Excitation Laser

1	Laser quantum	Gem 473nm 50mW	Diode pumped solid state laser, 473nm, 50mW
1	Laser quantum	Gem 561nm 50mW	Diode pumped solid state laser, 561nm, 50mW
8	Thorlabs	PH4	Ø1/2" Post Holder, Spring-Loaded Hex-Locking Thumbscrew, L = 4"

8	Thorlabs	TR8	Ø1/2" Optical Post, SS, 8-32 Setscrew, 1/4"-20 Tap, L = 8"
8	Thorlabs	TR1	Ø1/2" Optical Post, SS, 8-32 Setscrew, 1/4"-20 Tap, L = 1"
**Laser beam steering optics**			
9	Thorlabs	CF125	Clamping Fork, 1.24" Counterbored Slot, Universal
9	Thorlabs	PH4E	Ø1/2" Pedestal Post Holder, Spring-Loaded Hex-Locking Thumbscrew, L=4.19"
3	Thorlabs	LMR1	Imperial Lens Mount For 1" Optics, 8-32 Tap
8	Thorlabs	TR8	Ø1/2" Optical Post, SS, 8-32 Setscrew, 1/4"-20 Tap, L = 8"
1	Thorlabs	GBE02-A	2X Optical Beam Expander, AR Coated: 400 - 650 nm
1	Semrock	Di03-R660-T1-25X36	660 nm laser BrightLine single-edge superresolution laser dichroic beamsplitter
1	Semrock	LM01-503-25	503 nm edge LaserMUX™ single-edge laser-flat dichroic beamsplitter
1	Semrock	Di01-R488/561-25x36	488/561 nm lasers BrightLine® dual-edge laser-flat dichroic beamsplitter
4	Thorlabs	KM100-E02	Kinematic Mirror Mount for Ø1" Optics with Visible Laser Quality Mirror
1	Thorlabs	MFF101	Motorized Filter Flip Mount with Ø1" Optic Holder, 8-32 Tap
1	Thorlabs	SM1CP2	Externally SM1-Threaded End Cap
**Overhead IR illumination**			
1	Thorlabs	KPS101	15 V, 2.4 A Power Supply Unit for One K-Cube or T-Cube
1	Thorlabs	LEDD1B	T-Cube LED Driver, 1200 mA Max Drive Current
1	Thorlabs	M850F2	850 nm, 10.5 mW (Min) Fiber-Coupled LED, 1000 mA, SMA
1	Thorlabs	M28L01	Ø400 µm, 0.39 NA, SMA-SMA Fiber Patch Cable, Low OH, 1 Meter
1	Thorlabs	F240SMA-850	850 nm, f = mm, NA = SMA905 Fiber Collimation Pkg.
1	Thorlabs	BSN11R	25 x 36 mm 10:90 (R:T) UVFS Plate Beamsplitter, Coating: 700 - 1100 nm, t = 1 mm
1	Thorlabs	CF125	Clamping Fork, 1.24" Counterbored Slot, Universal
1	Thorlabs	PH4E	Ø1/2" Pedestal Post Holder, Spring-Loaded Hex-Locking Thumbscrew, L=6.19"
1	Thorlabs	CM1-DCH	30 mm Cage Cube with Dichroic Filter Mount
1	Thorlabs	AD12F	SM1-Threaded Adapter for Ø12 mm Cylindrical Components

1	Thorlabs	SM1L03	SM1 Lens Tube, 0.30" Thread Depth, One Retaining Ring Included
1	Thorlabs	TR8	Ø1/2" Optical Post, SS, 8-32 Setscrew, 1/4"-20 Tap, L = 8"
1	Thorlabs	TR4	Ø1/2" Optical Post, SS, 8-32 Setscrew, 1/4"-20 Tap, L = 4"
1	Thorlabs	RA90	Right-Angle Clamp for Ø1/2" Posts, 3/16" Hex

Other

1	National Instruments	782692-01	myRIO
1	National Instruments	PCIE-6351	X series pci express data acquisition
1	Teledyne Dalsa	Xcelera PX4, OR-X4C0-XPF00	Camera link frame grabber card
1	Neewer	TT560	Flash Speedlite
1	Thorlabs	TR8	Ø1/2" Optical Post, SS, 8-32 Setscrew, 1/4"-20 Tap, L = 8"
1	Thorlabs	CF125	Clamping Fork, 1.24" Counterbored Slot, Universal
1	Thorlabs	PH4E	Ø1/2" Pedestal Post Holder, Spring-Loaded Hex-Locking Thumbscrew, L=6.19"
1	Neewer	10076177	Flash rotation mount
1	Dell	T7810	Workstation (2x E5-2663 V3, 128GB, 2x 512GB SSD, NVS 295, Win 7 Pro)
2	Dell	U2412M	24" Ultrasharp Monitor
1	Arduino	A000066	Arduino Uno Rev3
1	BK Precision	1697	Programmable DC switching power supply, 0-40V, 0-5A
1	Thorlabs	TPS4	8" x 6" Straight Laser Safety Screen
2	Thorlabs	PH3	Ø1/2" Post Holder, Spring-Loaded Hex-Locking Thumbscrew, L = 3"
2	Thorlabs	TR4	Ø1/2" Optical Post, SS, 8-32 Setscrew, 1/4"-20 Tap, L = 4"

1		FlyceptionDiffuser.step	Custom delrin diffuser block
1		FlyceptionArena.step	Custom fly chamber
1	Vishay	VSMG3700	Custom 4"x4" 850nm LED array backlight
1		FlyceptionGlavoHolder.step	Custom galvo and dichroic mirror holder

1		FlyceptionArenaCalibration.step	Custom arena-view calibration delrin chamber
1		FlyceptionTargetHolder.step	Custom resolution target holder
2			Custom laser mounting plate
1			Custom flash control circuit
1			Custom Canon Lens control circuit arduino shield
1	Thorlabs	R1L3S10P	NBS 1952 Resolution Test Target, 3" x 1"
1	Thorlabs	MCWHL5	6500 K, 800 mW (Min) Mounted LED, 1000 mA
2	Thorlabs	LMR1AP	Alignment Plate for Ø1" Fixed Optic Mounts
1	Thorlabs	BSW11R	25 x 36 mm 50:50 UVFS Plate Beamsplitter, Coating: 700 - 1100 nm, t = 1 mm
1	Mitutoyo	570-312	HDS Digimatic Height Gage, 12" X .0005"/0.01mm, With Output

Supplementary Table 2. Parts list for Flyception2 system.
