# Supplementary material for: Imaging brain activity during complex social behaviors in Drosophila with Flyception2
Source: Nat Commun. 2020 Jan 30;11:623. doi: 10.1038/s41467-020-14487-7 (PMC6992788; doi:10.1038/s41467-020-14487-7)
Supplement: Supplementary file 20 — Supplementary Software 1 [file 41467_2020_14487_MOESM20_ESM.zip › Supplementary Software 1/Altium/ArduinoLensControlAltium.zip!/Altium/20171121 Arduino Shield Lens Control/ArduinoUnoShield.PcbDoc.htm]

Reporting Options

# File in Previous Format

|  |  |  |
| --- | --- | --- |
| Date | : | 11/21/2017 |
| Time | : | 10:35:33 AM |
| Filename | : | C:\Users\fcardone\Desktop\Temp Google Drive Folder\20171121 Arduino Shield for Lens Control\Altium\20171121 Arduino Shield Lens Control\ArduinoUnoShield.PcbDoc |

  

| Version | Warning |
| --- | --- |
| Release 13 | **CAUTION** - Silkscreen Over Component Pads Rules are converted to Silk To Solder Mask Clearance Rules. Suggest examining rule scopes for accuracy. |

  

---

This file was generated by **an earlier** version of the software
