# Supplementary material for: Imaging brain activity during complex social behaviors in Drosophila with Flyception2
Source: Nat Commun. 2020 Jan 30;11:623. doi: 10.1038/s41467-020-14487-7 (PMC6992788; doi:10.1038/s41467-020-14487-7)
Supplement: Supplementary file 20 — Supplementary Software 1 [file 41467_2020_14487_MOESM20_ESM.zip › Supplementary Software 1/Altium/ArduinoLensControlAltium.zip!/Altium/20171121 Arduino Shield Lens Control/DF3EA-8P-2H(50)/DF3_catalog.pdf]

2mm Pitch Connector for Discrete Wire Connection(Product approved by UL CSA Standard)

DF3 Series

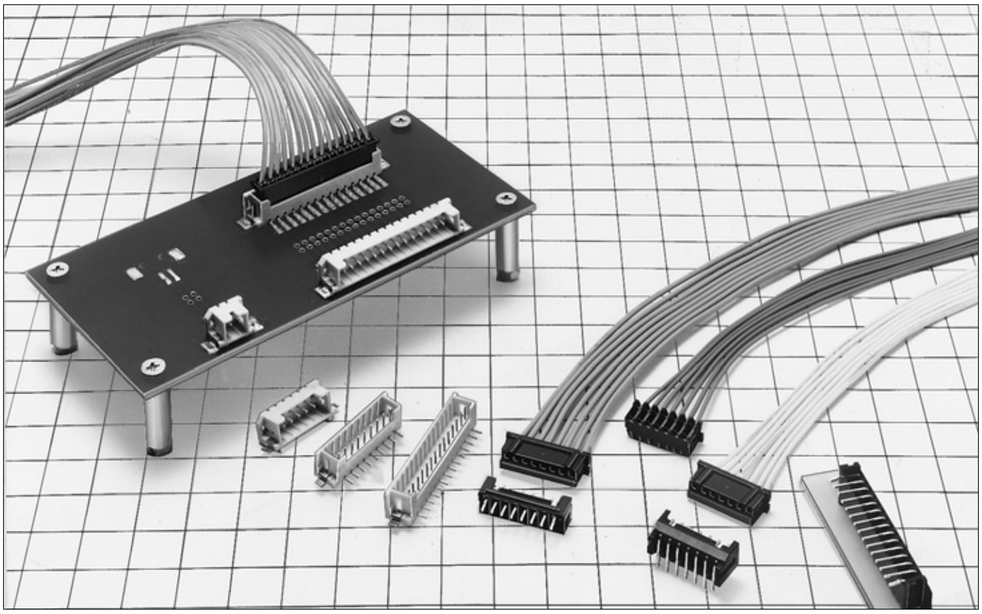

Nov.1.2017 Copyright 2017 HIROSE ELECTRIC CO., LTD. All Rights Reserved.

■Features

1. Correspond to Board Design in Wide Variation

●ID Socket
(1) Low profile design with the mounting height of 6.8mm
(2) Achieves the multi-harness by the full automatic ID machine in combination with the DF11 series ID type.
(DF11 series represents the 2mm pitch single-row product.)

●Crimp Socket
(1) Low profile design with mounting height 7.5mm.
(2) Provide the contact crimping area with the sufficient space, and secures the stable crimping operation.

●Receptacle
(1) The board can be connected horizontally or vertically in combination with the straight header with the right angle header.
(2) For horizontal connection, the board to board size is the low profile of 6.6mm.
(3) Compatibility with the in-line plug allows the receptacle to set the board side to the receptacle and the cable side to the plug in the board to cable connection.

●Pin Header
(1) The board pre-fixed kink is treated with the post pin.
(2) The style employs a scoop-proof and sturborn box type.
(3) The type corresponding to SMT is avilable, using the heat-proof resin.
This SMT header is equipped with the metal tab to prevent peeling in the solder joint area.

●In-line Plug
(1) The crimping in-line plug consits of two types, one type, which has pursued after space merits, and the other employs the panel lock type, which can be fixed to the panel.
(2) The in-line adapter is available to insert and in-line the ID socket from both sides.

2. UL, CSA Certified

3. Glow Wire Compliance (Compliant with IEC 60695-2-11)

■Applications

Business equipment such as copy machine and printer

■Product Specifications

Rating	Current rating Note 1	Crimping	Wire size		22 to 24 AWG : 3A<br>26 AWG : 2A<br>28 AWG : 1A	Operating Temperature Range	-55℃ to +105℃ (Note 2)
						Operating Humidity Range	40% to 80% (Note 3)
		IDC	Wire size		26, 28AWG :1A	Storage Temperature Range	-10℃ to +60℃ (Note 4)
	Voltage rating	Crimping	250V AC	IDC	100V AC	Storage Humidity Range	40% to 70% (Note 4)

UL-CSA Safety Standard and Approved specification			
Rating	Rated Current	Wire size	24 AWG : 3A<br>26 AWG : 2A (Note 5)<br>28 AWG : 1A
	Rated Voltage	30V AC	

■UL CSA Safety Standard File No.
UL : E52653
CSA : LR95109

Item	Specification	Condition		
1. Insulation Resistance	1000MΩ min.	500V DC		
2. Withstanding voltage	No flashover or insulation breakdown.	650V AC, 1 minute		
3. Contact Resistance	30mΩ max	100mA		
4. Single Insertor/Extraction Force	0.3N(30gf)min, 4.4N(450gf)max.	Measure by the square steel pin with 0.5±0.002mm		
5. Vibration	No electrical discontinuity of 1μs or more	Frequency : 10 to 55 Hz, single amplitude of 0.75 mm, 2 hours in each of the 3 directions.		
6. Humidity (Steady state)	Contact resistance : 30mΩ max. Insulation resistance : 500MΩ min.	96 hours at temperature of 40℃ and humidity of 90% to 95%		
7. Temperature Cycle	Contact resistance : 30mΩ max. Insulation resistance :1000MΩ min.	(-55℃ : 30 minutes 5 to 35℃: 10 minutes 85℃: 30 minutes 5 to 35℃: 10minutes) 5 cycles		
8. Durability (Mating/un-mating)	Contact resistance : 30mΩ max.	Tin plating : 30 cycles Gold plating: 50 cycles		
9. Resistance to Soldering Heat	No deformation of components affecting performance.	THT type	Flow : 260℃ for 10 seconds	Manual soldering : 300℃ for 2 seconds
		SMT type	Reflow : At the recommended temperature profile	Manual soldering : 300℃ for 3 seconds

Note 1 : Current rating of header is 3A.
Note 2 : Includes temperature rise caused by current flow.
Apply to crimping contact type. Maximum operating temperature for IDC and receptacle type is 85℃.
Note 3 : Please use under conditions with no condensation occurred.
Note 4 : The term "storage" refers to products stored for long period of time prior to mounting and use. Operating Temperature Range and Humidity range covers non conducting condition of installed connectors in storage, shipment or during transportation.
Note 5 : Apply to crimping contact type. The IDC type's rated current is 1A.

■Materials / Finish

Product	Part	Material	Finish	Remarks
Crimping Socket	Insulator	Polyamide	Black	UL94V-0
IDC Contact for Socket	Contact	Phosphor bronze	Tin plated or gold plated	———
ID Socket	Insulator	Polyamide	Black	UL94V-0
	Contact	Phosphor bronze	Tin plated or gold plated	———
Receptacle	Insulator	Polyamide	Black	UL94V-0
	Contact	Phosphor bronze	Tin plated or gold plated	———
Pin Header	Insulator	Polyamide	Black	UL94V-0
	Contact	Brass	Tin plated or gold plated	———
SMT Pin Header	Insulator	Polyamide	Beige	UL94V-0
	Contact	Brass	Tin plated or gold plated	———
	Metal tab	Brass	Tin plated	———
In-line Plug	Insulator	Polyamide	Black	UL94V-0
In-line IDC Contact	Contact	Phosphor bronze	Tin plated or gold plated	———
In-line Adapter	Insulator	Polyamide	Black	UL94V-0
	Contact	Brass	Tin plated	———

Nov.1.2017 Copyright 2017 HIROSE ELECTRIC CO., LTD. All Rights Reserved.

■Product Number Structure

Refer to the chart below when determining the product specifications from the product number.
Please select from the product numbers listed in this catalog when placing orders.

●Connector

DF3 - ＊ S - 2C

❶ ❷ ❸ ❹❺

❶ Series Name : DF3, 3A, 3AA, 3EA	❺ Connection Form/Contact Style
❷ Number of Contacts : 2~15	C : Crimp socket
❸ Connector Type	R26 : ID socket for 26AWG
S : Socket	R28 : ID socket for 28AWG
P : Pin Header	DSA : Straight through hole
EP : In-line Plug	DS : Right angle through hole
❹ Contact Pitch : 2mm	V : Straight SMT
	H : Right angle SMT
	A : Adapter

●Contact

DF3 - EP2428PCFA

❶ ❷ ❸ ❹

❶ Contact Type	❸ Contact Type/Packaging Type
Blank : For socket	SCF : Socket contact, Reel
EP : For in-line plug	SC : Socket contact, Pack
❷ Applicable Wire Size	PCF : Plug contact, Reel
22 : 22 AWG	PC : Plug contact, Pack
2428 : 24 to 28 AWG	❹ Plating Type
	Blank : Tin plated
	A : Gold plated (Plug contact)
	C : Gold plated (Socket contact)

■Application pattern

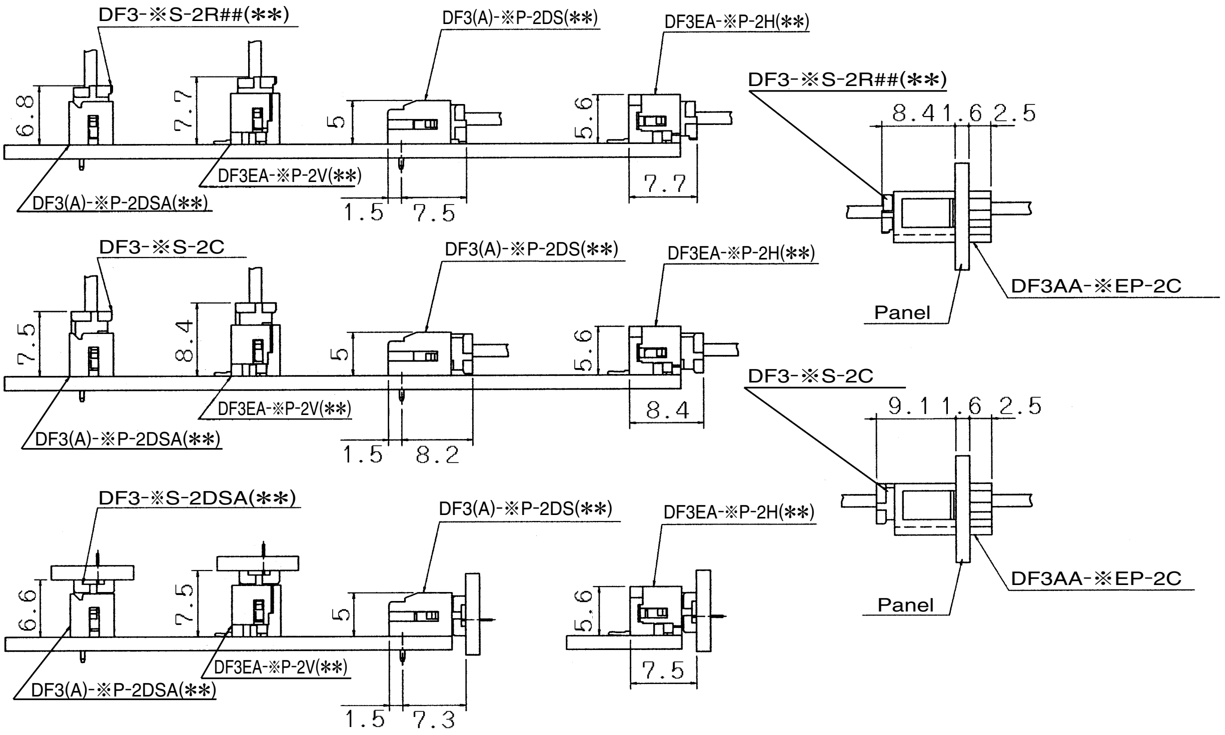

Nov.1.2017 Copyright 2017 HIROSE ELECTRIC CO., LTD. All Rights Reserved.

Nov.1.2017 Copyright 2017 HIROSE ELECTRIC CO., LTD. All Rights Reserved.

■ID Socket

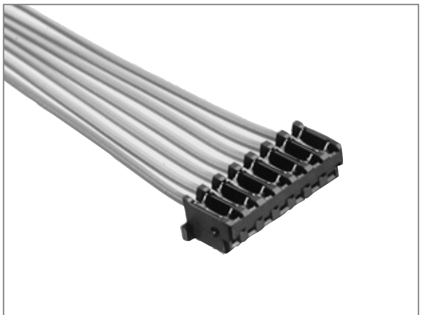

This picture shows the cable assembled status.

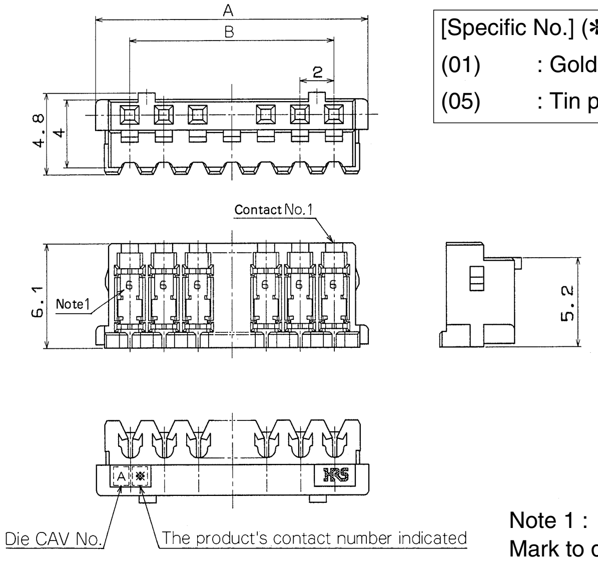

[Specific No.] (✱✱), ✱✱
(01) : Gold plated, 100pcs/pack
(05) : Tin plated, 100pcs/pack

Note 1 :
Mark to determine the socket name
Mark6 : R26, Mark8 : R28

●26 AWG

Unit : mm

Part No.	HRS No.	No. of Contacts	A	B	RoHS
DF3- 2S-2R26(✱✱)	543-0078-7 ✱✱	2	6	2	YES
DF3- 3S-2R26(✱✱)	543-0079-0 ✱✱	3	8	4	
DF3- 4S-2R26(✱✱)	543-0080-9 ✱✱	4	10	6	
DF3- 5S-2R26(✱✱)	543-0081-1 ✱✱	5	12	8	
DF3- 6S-2R26(✱✱)	543-0082-4 ✱✱	6	14	10	
DF3- 7S-2R26(✱✱)	543-0083-7 ✱✱	7	16	12	
DF3- 8S-2R26(✱✱)	543-0084-0 ✱✱	8	18	14	
DF3- 9S-2R26(✱✱)	543-0085-2 ✱✱	9	20	16	
DF3-10S-2R26(✱✱)	543-0086-5 ✱✱	10	22	18	
DF3-11S-2R26(✱✱)	543-0087-8 ✱✱	11	24	20	
DF3-12S-2R26(✱✱)	543-0088-0 ✱✱	12	26	22	
DF3-13S-2R26(✱✱)	543-0089-3 ✱✱	13	28	24	
DF3-14S-2R26(✱✱)	543-0090-2 ✱✱	14	30	26	
DF3-15S-2R26(✱✱)	543-0091-5 ✱✱	15	32	28	

●28 AWG

Unit : mm

Part No.	HRS No.	No. of Contacts	A	B	RoHS
DF3- 2S-2R28(✱✱)	543-0092-8 ✱✱	2	6	2	YES
DF3- 3S-2R28(✱✱)	543-0093-0 ✱✱	3	8	4	
DF3- 4S-2R28(✱✱)	543-0094-3 ✱✱	4	10	6	
DF3- 5S-2R28(✱✱)	543-0095-6 ✱✱	5	12	8	
DF3- 6S-2R28(✱✱)	543-0096-9 ✱✱	6	14	10	
DF3- 7S-2R28(✱✱)	543-0097-1 ✱✱	7	16	12	
DF3- 8S-2R28(✱✱)	543-0098-4 ✱✱	8	18	14	
DF3- 9S-2R28(✱✱)	543-0099-7 ✱✱	9	20	16	
DF3-10S-2R28(✱✱)	543-0100-4 ✱✱	10	22	18	
DF3-11S-2R28(✱✱)	543-0101-7 ✱✱	11	24	20	
DF3-12S-2R28(✱✱)	543-0102-0 ✱✱	12	26	22	
DF3-13S-2R28(✱✱)	543-0103-2 ✱✱	13	28	24	
DF3-14S-2R28(✱✱)	543-0104-5 ✱✱	14	30	26	
DF3-15S-2R28(✱✱)	543-0105-8 ✱✱	15	32	28	

Note 2 : The quantity for delivery of the specific No. (01) and (05) is determined per bag (100pcs.). If needed, please order the productsper bag.

■Applicable Cable

Part No.	Applicable Cable	Jacket Diameter
DF3-✱S-2R26(✱✱)	UL1061 26 AWG (7/0.16)	ϕ0.9 to 1mm
DF3-✱S-2R28(✱✱)	UL1061 28 AWG (7/0.127)	ϕ0.9 to 1mm

■Applicable ID Connection Tool

Type	Part No.	Connection Type	Remarks
DF3 DF4 Desktop ID Machine	SA701/DF3	DF3 No restriction	Desktop machine for small lot

Nov.1.2017 Copyright 2017 HIROSE ELECTRIC CO., LTD. All Rights Reserved.

■Crimping Socket

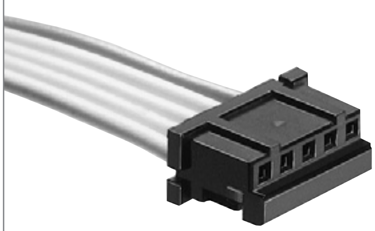

This picture shows the Contact crimped status.

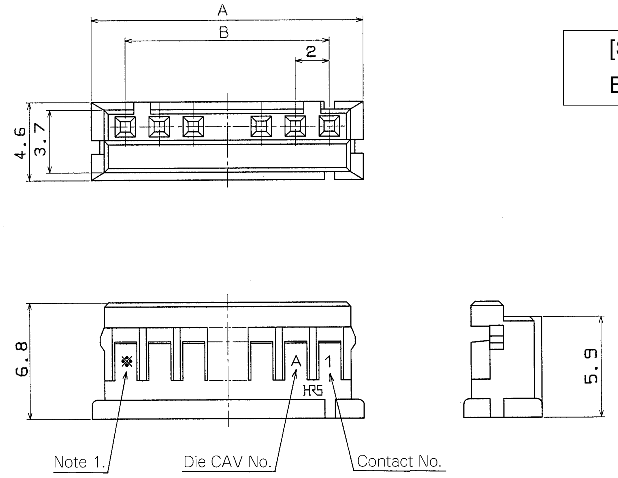

[Specific No.] (✱✱), ✱✱
Blank : 100pcs/pack

Unit : mm

Part No.	HRS No.	No. of Contacts	A	B	RoHS	Part No.	HRS No.	No. of Contacts	A	B	RoHS
DF3-2S-2C(✱✱)	543-0003-8 ✱✱	2	6	2	YES	DF3- 9S-2C(✱✱)	543-0010-3 ✱✱	9	20	16	YES
DF3-3S-2C(✱✱)	543-0004-0 ✱✱	3	8	4		DF3-10S-2C(✱✱)	543-0011-6 ✱✱	10	22	18	
DF3-4S-2C(✱✱)	543-0005-3 ✱✱	4	10	6		DF3-11S-2C(✱✱)	543-0012-9 ✱✱	11	24	20	
DF3-5S-2C(✱✱)	543-0006-6 ✱✱	5	12	8		DF3-12S-2C(✱✱)	543-0013-1 ✱✱	12	26	22	
DF3-6S-2C(✱✱)	543-0007-9 ✱✱	6	14	10		DF3-13S-2C(✱✱)	543-0014-4 ✱✱	13	28	24	
DF3-7S-2C(✱✱)	543-0008-1 ✱✱	7	16	12		DF3-14S-2C(✱✱)	543-0015-7 ✱✱	14	30	26	
DF3-8S-2C(✱✱)	543-0009-4 ✱✱	8	18	14		DF3-15S-2C(✱✱)	543-0016-0 ✱✱	15	32	28	

Note1 : The product's contact number indicated.
Note2 : The quantity for delivery of the specification No. "None" is determined per bag (100 pcs.).
If needed, please order the products per bag.

■Receptacle

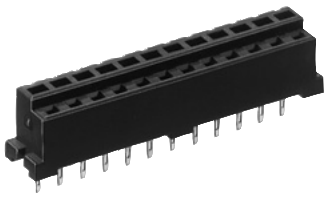

●Board Through-hole Diameter : $\phi 0.8^{+0.1}_{0}$

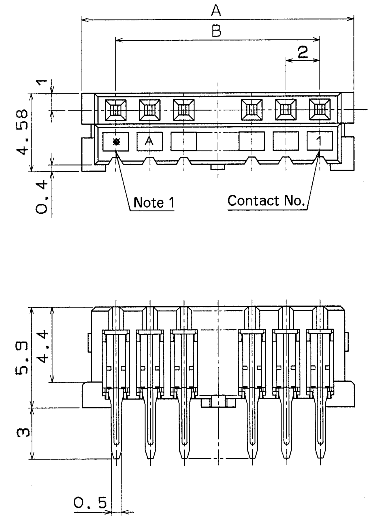

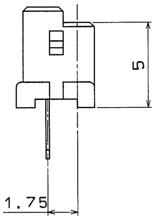

[Specific No.] (✱✱), ✱✱
(25) : Tin plated, Tube packaging
(55) : Gold plated, Tube packaging

Note1 : The product's contact number indicated

Unit: mm

Part No.	HRS No.	No. of Contacts	A	B	Tube Package Quantity	RoHS	Part No.	HRS No.	No. of Contacts	A	B	Tube Package Quantity	RoHS
DF3-2S-2DSA(✱✱)	543-0048-6 ✱✱	2	6	2	80 pieces	YES	DF3- 9S-2DSA(✱✱)	543-0055-1 ✱✱	9	20	16	24 pieces	YES
DF3-3S-2DSA(✱✱)	543-0049-9 ✱✱	3	8	4	60		DF3-10S-2DSA(✱✱)	543-0056-4 ✱✱	10	22	18	21	
DF3-4S-2DSA(✱✱)	543-0050-8 ✱✱	4	10	6	48		DF3-11S-2DSA(✱✱)	543-0057-7 ✱✱	11	24	20	20	
DF3-5S-2DSA(✱✱)	543-0051-0 ✱✱	5	12	8	40		DF3-12S-2DSA(✱✱)	543-0058-0 ✱✱	12	26	22	18	
DF3-6S-2DSA(✱✱)	543-0052-3 ✱✱	6	14	10	34		DF3-13S-2DSA(✱✱)	543-0059-2 ✱✱	13	28	24	17	
DF3-7S-2DSA(✱✱)	543-0053-6 ✱✱	7	16	12	30		DF3-14S-2DSA(✱✱)	543-0060-1 ✱✱	14	30	26	16	
DF3-8S-2DSA(✱✱)	543-0054-9 ✱✱	8	18	14	27		DF3-15S-2DSA(✱✱)	543-0061-4 ✱✱	15	32	28	15	

Note2 : Please order the tube packaging at the multiplied tube packaging quantity (pieces).

■Straight Pin Header

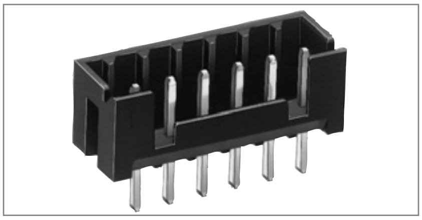

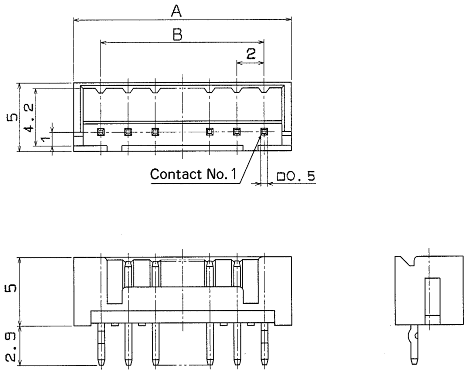

●Board Through-hole Diameter: $\phi0.8^{+0.1}_{0}$
●Kink Pre-fixed Effective Diameter: $\phi0.8^{+0.05}_{0}$

Unit : mm

Tin Plating		Gold Plating		No. of Contacts	A	B	RoHS
Part No.	HRS No.	Part No.	HRS No.				
DF3A- 2P-2DSA	543-0165-0	DF3- 2P-2DSA(01)	543-0017-2 01	2	6	2	YES
DF3A- 3P-2DSA	543-0166-2	DF3- 3P-2DSA(01)	543-0018-5 01	3	8	4	
DF3A- 4P-2DSA	543-0167-5	DF3- 4P-2DSA(01)	543-0019-8 01	4	10	6	
DF3A- 5P-2DSA	543-0168-8	DF3- 5P-2DSA(01)	543-0020-7 01	5	12	8	
DF3A- 6P-2DSA	543-0169-0	DF3- 6P-2DSA(01)	543-0021-0 01	6	14	10	
DF3A- 7P-2DSA	543-0170-0	DF3- 7P-2DSA(01)	543-0022-2 01	7	16	12	
DF3A- 8P-2DSA	543-0171-2	DF3- 8P-2DSA(01)	543-0023-5 01	8	18	14	
DF3A- 9P-2DSA	543-0172-5	DF3- 9P-2DSA(01)	543-0024-8 01	9	20	16	
DF3A-10P-2DSA	543-0173-8	DF3-10P-2DSA(01)	543-0025-0 01	10	22	18	
DF3A-11P-2DSA	543-0174-0	DF3-11P-2DSA(01)	543-0026-3 01	11	24	20	
DF3A-12P-2DSA	543-0175-3	DF3-12P-2DSA(01)	543-0027-6 01	12	26	22	
DF3A-13P-2DSA	543-0176-6	DF3-13P-2DSA(01)	543-0028-9 01	13	28	24	
DF3A-14P-2DSA	543-0177-9	DF3-14P-2DSA(01)	543-0029-1 01	14	30	26	
DF3A-15P-2DSA	543-0178-1	DF3-15P-2DSA(01)	543-0030-0 01	15	32	28	

Note : The quantity for delivery is determined per bag (100 pcs.). If needed, please order the products per bag.

■Right Angle Pin Header

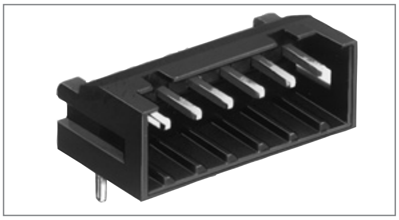

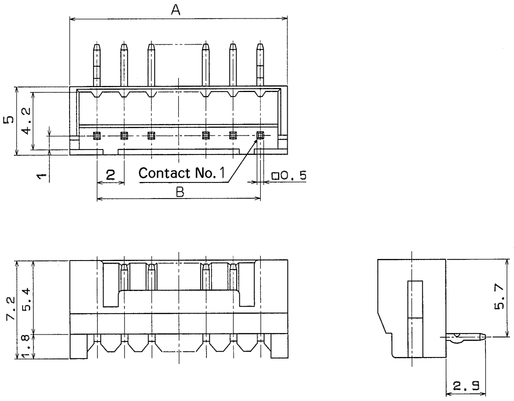

●Board Through-hole Diameter: $\phi0.8^{+0.1}_{0}$
●Kink Pre-fixed Effective Diameter: $\phi0.8^{+0.05}_{0}$

Unit : mm

Tin Plating		Gold Plating		No. of Contacts	A	B	RoHS
Part No.	HRS No.	Part No.	HRS No.				
DF3A- 2P-2DS	543-0179-4	DF3- 2P-2DS(01)	543-0031-3 01	2	6	2	YES
DF3A- 3P-2DS	543-0180-3	DF3 -3P-2DS(01)	543-0032-6 01	3	8	4	
DF3A- 4P-2DS	543-0181-6	DF3- 4P-2DS(01)	543-0033-9 01	4	10	6	
DF3A- 5P-2DS	543-0182-9	DF3- 5P-2DS(01)	543-0034-1 01	5	12	8	
DF3A- 6P-2DS	543-0183-1	DF3- 6P-2DS(01)	543-0035-4 01	6	14	10	
DF3A- 7P-2DS	543-0184-4	DF3- 7P-2DS(01)	543-0036-7 01	7	16	12	
DF3A- 8P-2DS	543-0185-7	DF3- 8P-2DS(01)	543-0037-0 01	8	18	14	
DF3A- 9P-2DS	543-0186-0	DF3- 9P-2DS(01)	543-0038-2 01	9	20	16	
DF3A-10P-2DS	543-0187-2	DF3-10P-2DS(01)	543-0039-5 01	10	22	18	
DF3A-11P-2DS	543-0188-5	DF3-11P-2DS(01)	543-0040-4 01	11	24	20	
DF3A-12P-2DS	543-0189-8	DF3-12P-2DS(01)	543-0041-7 01	12	26	22	
DF3A-13P-2DS	543-0190-7	DF3-13P-2DS(01)	543-0042-0 01	13	28	24	
DF3A-14P-2DS	543-0191-0	DF3-14P-2DS(01)	543-0043-2 01	14	30	26	
DF3A-15P-2DS	543-0192-2	DF3-15P-2DS(01)	543-0044-5 01	15	32	28	

Note : The quantity for delivery is determined per bag(100pcs.). If needed, please order the products per bag.

Nov.1.2017 Copyright 2017 HIROSE ELECTRIC CO., LTD. All Rights Reserved.

■Straight Pin Header (SMT)

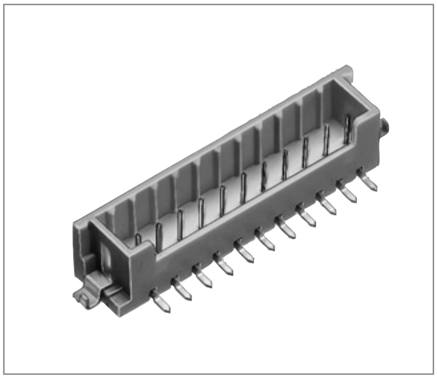

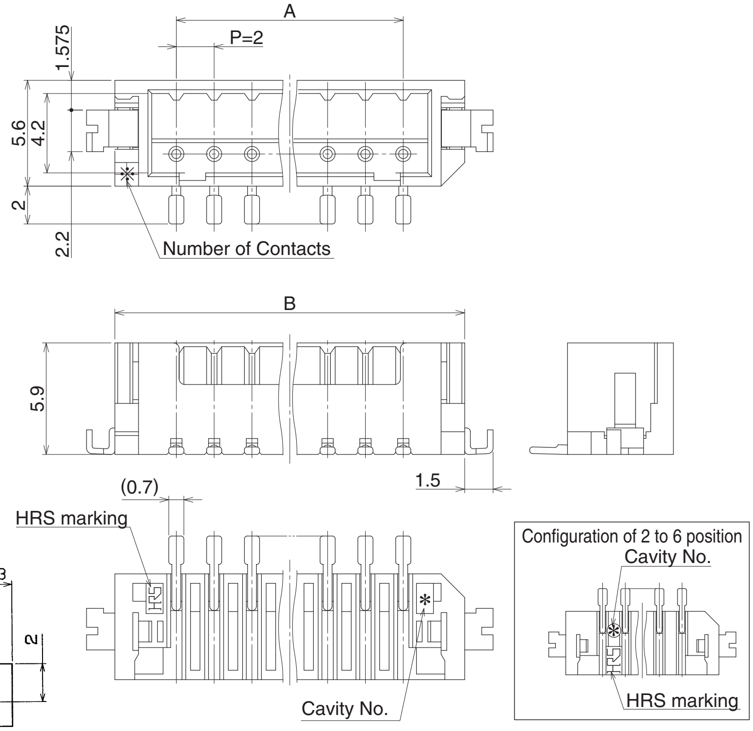

◆PCB mounting pattern

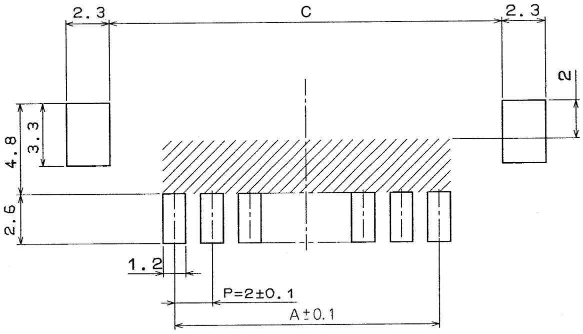

Note : ▨ If a pattern is included in the shaded area and is not treated with resist, it could touch the connector contact.

[Specific No.] (✻✻), ✻✻
(21) : Tin plated packaging
(51) : Gold plated packaging

Unit : mm

Part No.	HRS No.	No. of Contacts	A	B	C	RoHS
DF3EA-2P-2V(✻✻)	543-0315-0 ✻✻	2	2	8.5	8.74	YES
DF3EA-3P-2V(✻✻)	543-0316-0 ✻✻	3	4	10.5	10.74	
DF3EA-4P-2V(✻✻)	543-0317-0 ✻✻	4	6	12.5	12.74	
DF3EA-5P-2V(✻✻)	543-0318-0 ✻✻	5	8	14.5	14.74	
DF3EA-6P-2V(✻✻)	543-0319-0 ✻✻	6	10	16.5	16.74	
DF3EA-7P-2V(✻✻)	543-0320-0 ✻✻	7	12	18.5	18.74	
DF3EA-8P-2V(✻✻)	543-0321-0 ✻✻	8	14	20.5	20.74	
DF3EA-9P-2V(✻✻)	543-0322-0 ✻✻	9	16	22.5	22.74	
DF3EA-10P-2V(✻✻)	543-0323-0 ✻✻	10	18	24.5	24.74	
DF3EA-11P-2V(✻✻)	543-0324-0 ✻✻	11	20	26.5	26.74	
DF3EA-12P-2V(✻✻)	543-0325-0 ✻✻	12	22	28.5	28.74	
DF3EA-13P-2V(✻✻)	543-0326-0 ✻✻	13	24	30.5	30.74	
DF3EA-14P-2V(✻✻)	543-0327-0 ✻✻	14	26	32.5	32.74	
DF3EA-15P-2V(✻✻)	543-0328-0 ✻✻	15	28	34.5	34.74	

Note1 : Please order the emboss packaging product by the reel number. (1 reel : 1000pcs.)

Nov.1.2017 Copyright 2017 HIROSE ELECTRIC CO., LTD. All Rights Reserved.

■Right Angle Pin Header (SMT)

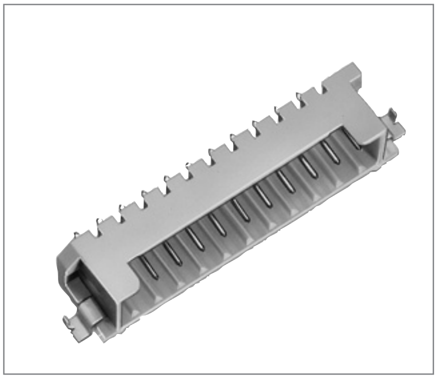

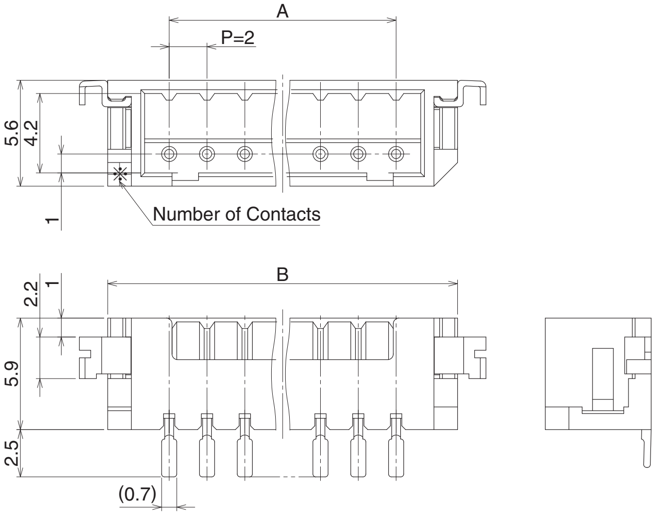

◆PCB mounting pattern

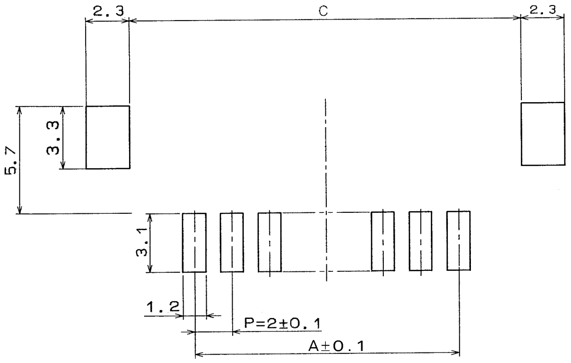

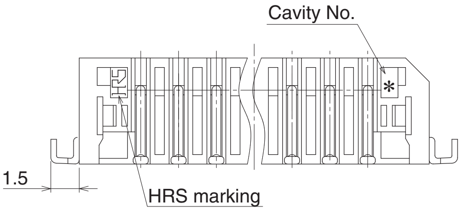

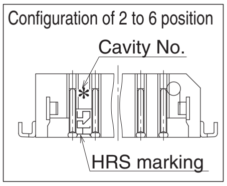

[Specific No.] (✻✻), ✻✻
(21) : Tin plated embossed packaging
(51) : Gold plated embossded packaging

Unit : mm

Part No.	HRS No.	No. of Contacts	A	B	C	RoHS
DF3EA-2P-2H(✻✻)	543-0329-0 ✻✻	2	2	8.5	8.74	YES
DF3EA-3P-2H(✻✻)	543-0330-0 ✻✻	3	4	10.5	10.74	
DF3EA-4P-2H(✻✻)	543-0331-0 ✻✻	4	6	12.5	12.74	
DF3EA-5P-2H(✻✻)	543-0332-0 ✻✻	5	8	14.5	14.74	
DF3EA-6P-2H(✻✻)	543-0333-0 ✻✻	6	10	16.5	16.74	
DF3EA-7P-2H(✻✻)	543-0334-0 ✻✻	7	12	18.5	18.74	
DF3EA-8P-2H(✻✻)	543-0335-0 ✻✻	8	14	20.5	20.74	
DF3EA-9P-2H(✻✻)	543-0336-0 ✻✻	9	16	22.5	22.74	
DF3EA-10P-2H(✻✻)	543-0337-0 ✻✻	10	18	24.5	24.74	
DF3EA-11P-2H(✻✻)	543-0338-0 ✻✻	11	20	26.5	26.74	
DF3EA-12P-2H(✻✻)	543-0339-0 ✻✻	12	22	28.5	28.74	
DF3EA-13P-2H(✻✻)	543-0340-0 ✻✻	13	24	30.5	30.74	
DF3EA-14P-2H(✻✻)	543-0341-0 ✻✻	14	26	32.5	32.74	
DF3EA-15P-2H(✻✻)	543-0342-0 ✻✻	15	28	34.5	34.74	

Note1 : Please order the emboss packaging product by the reel number. (1reel : 1000pcs.)

Nov.1.2017 Copyright 2017 HIROSE ELECTRIC CO., LTD. All Rights Reserved.

■In-line Plug

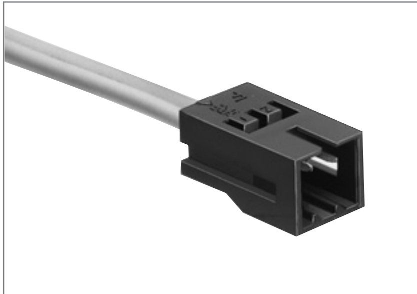

Photo: Contact inserted status

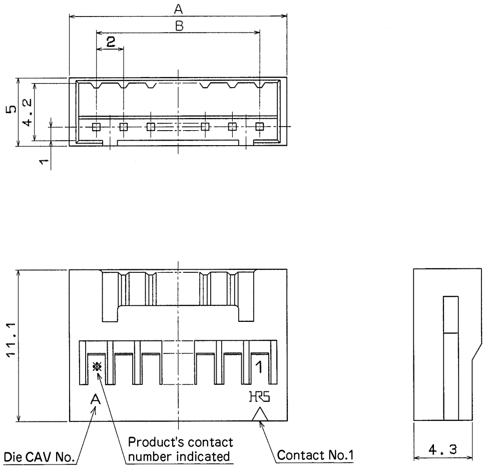

Unit : mm

Part No.	HRS No.	No. of Contacts	A	B	RoHS	Part No.	HRS No.	No. of Contacts	A	B	RoHS
DF3- 2EP-2C	543-0064-2	2	6	2		DF3- 9EP-2C	543-0071-8	9	20	16	
DF3- 3EP-2C	543-0065-5	3	8	4		DF3-10EP-2C	543-0072-0	10	22	18	
DF3- 4EP-2C	543-0066-8	4	10	6		DF3-11EP-2C	543-0073-3	11	24	20	
DF3- 5EP-2C	543-0067-0	5	12	8	YES	DF3-12EP-2C	543-0074-6	12	26	22	YES
DF3- 6EP-2C	543-0068-3	6	14	10		DF3-13EP-2C	543-0075-9	13	28	24	
DF3- 7EP-2C	543-0069-6	7	16	12		DF3-14EP-2C	543-0076-1	14	30	26	
DF3- 8EP-2C	543-0070-5	8	18	14		DF3-15EP-2C	543-0077-4	15	32	28	

Note : The specific No."None" item is delivered per bag (100pcs.). Please order products per bag unit.

■In-line Plug with Panel Lock

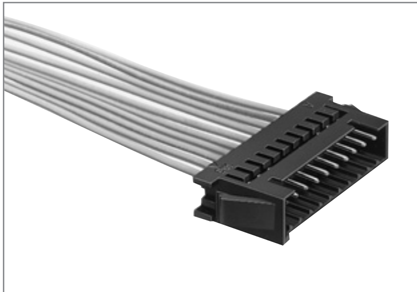

Photo: Contact insertion status

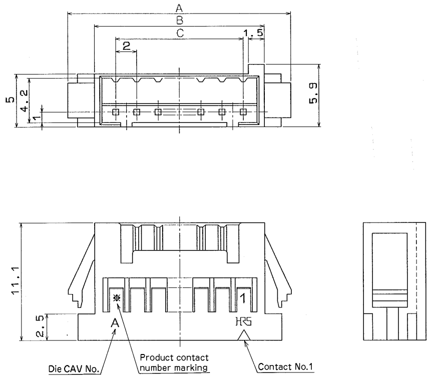

Unit : mm

Part No.	HRS No.	No. of Contacts	A	B	C	D	E	RoHS
DF3AA- 2EP-2C	543-0151-5	2	11	6	2	9.4	6.3	
DF3AA- 3EP-2C	543-0152-8	3	13	8	4	11.4	8.3	
DF3AA- 4EP-2C	543-0153-0	4	15	10	6	13.4	10.3	
DF3AA- 5EP-2C	543-0154-3	5	17	12	8	15.4	12.3	
DF3AA- 6EP-2C	543-0155-6	6	19	14	10	17.4	14.3	
DF3AA- 7EP-2C	543-0156-9	7	21	16	12	19.4	16.3	
DF3AA- 8EP-2C	543-0157-1	8	23	18	14	21.4	18.3	
DF3AA- 9EP-2C	543-0158-4	9	25	20	16	23.4	20.3	YES
DF3AA-10EP-2C	543-0159-7	10	27	22	18	25.4	22.3	
DF3AA-11EP-2C	543-0160-6	11	29	24	20	27.4	24.3	
DF3AA-12EP-2C	543-0161-9	12	31	26	22	29.4	26.3	
DF3AA-13EP-2C	543-0162-1	13	33	28	24	31.4	28.3	
DF3AA-14EP-2C	543-0163-4	14	35	30	26	33.4	30.3	
DF3AA-15EP-2C	543-0164-7	15	37	32	28	35.4	32.3	

Note : The specific No. "None" item is delivered per bag (100pcs.). Please order products per bag unit.

◆Panel Cutouts

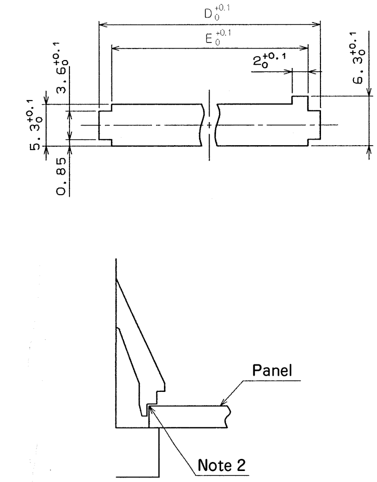

Note1 : Applicable panel thickness is 1.6 to 1.0mm. (However, a slight backlash is considered for the thickness other than 1.6 and 1.2mm.)

Note2 : Use the non-radius surface at the hooking panel edge.

Note3 : Contact No. indicates 1, 4, 8, 12, and 15.

Nov.1.2017 Copyright 2017 HIROSE ELECTRIC CO., LTD. All Rights Reserved.

■In-line Adapter with Panel Lock

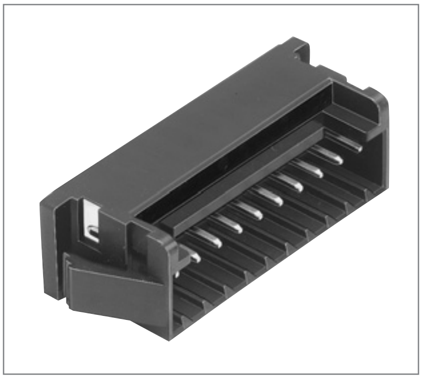

Adapter to in-line by pushing 2pcs. of socket from both sides
No.1-No.1 connection circuit type

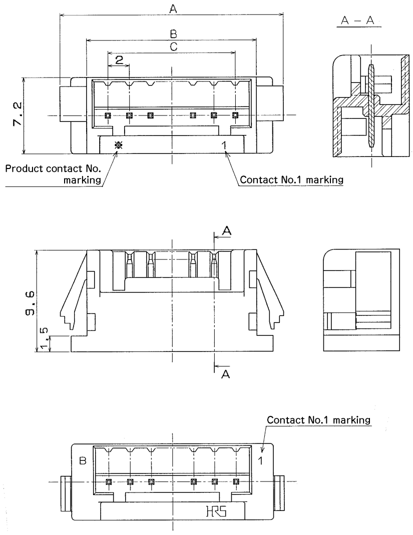

Unit : mm

Part No.	HRS No.	No. of Contacts	A	B	C	D	E	RoHS
DF3- 2EP-2A	543-0267-0	2	11	6	2	9.4	6.3	YES
DF3- 3EP-2A	543-0268-2	3	13	8	4	11.4	8.3	
DF3- 4EP-2A	543-0269-5	4	15	10	6	13.4	10.3	
DF3- 5EP-2A	543-0270-4	5	17	12	8	15.4	12.3	
DF3- 6EP-2A	543-0271-7	6	19	14	10	17.4	14.3	
DF3- 7EP-2A	543-0272-0	7	21	16	12	19.4	16.3	
DF3- 8EP-2A	543-0273-2	8	23	18	14	21.4	18.3	
DF3- 9EP-2A	543-0274-5	9	25	20	16	23.4	20.3	
DF3-10EP-2A	543-0275-8	10	27	22	18	25.4	22.3	
DF3-11EP-2A	543-0276-0	11	29	24	20	27.4	24.3	
DF3-12EP-2A	543-0277-3	12	31	26	22	29.4	26.3	
DF3-13EP-2A	543-0278-6	13	33	28	24	31.4	28.3	
DF3-14EP-2A	543-0279-9	14	35	30	26	33.4	30.3	
DF3-15EP-2A	543-0280-8	15	37	32	28	35.4	32.3	

Note : The specific No. "None" item is delivered per bag (100pcs.). Please order products per bag unit.

◆Panel Cutouts

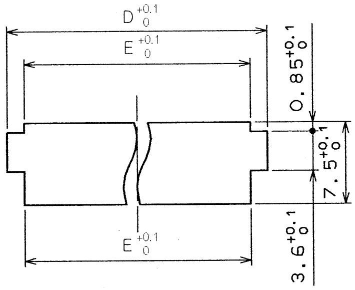

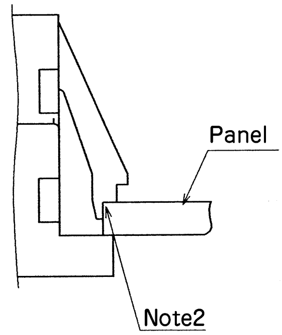

Note1 : Applicable panel thickness is 1.6 to 1.0mm.(However,backlash will occur except for 1.6, 1.2mm thickness.)
Note2 : Please use the non-radius surface at the hooking panel edge.

Nov.1.2017 Copyright 2017 HIROSE ELECTRIC CO., LTD. All Rights Reserved.

■Crimping Contact for Socket

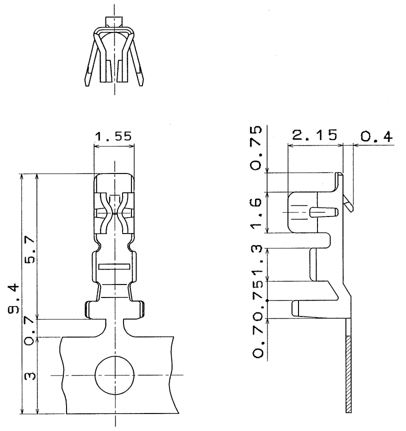

Part No.	HRS No.	Applicable Cable				Packaging	Finish	RoHS
		ULstyle	Jacket Diameter	Wire Size	Stranded Wire Conductor			
DF3-22SCF	543-0235-3	1061	1.2-1.5	22 AWG	17/0.16	10,000/reel	Tin plated	YES
DF3-22SC	543-0236-6	1061	1.3	22 AWG	17/0.16	100/pack		
DF3-22SCFC	543-0861-0	1061	1.2-1.5	22 AWG	17/0.16	10,000/reel	Gold plated	
DF3-22SCC	543-0862-3	1061	1.3	22 AWG	17/0.16	100/pack		
DF3-2428SCF	543-0001-2	1007 1061	0.75 to 1.5	24 AWG	11/0.16	10,000/reel	Tin plated	
				26 AWG	7/0.16			
				28 AWG	7/0.127			
DF3-2428SC	543-0002-5	1007	1.5	24 AWG	11/0.16	100/pack		
			1.3	26 AWG	7/0.16			
			1.2	28 AWG	7/0.127			
DF3-2428SCFC	543-0046-0	1007 1061	0.75 to 1.5	24 AWG	11/0.16	10,000/reel	Gold plated	
				26 AWG	7/0.16			
				28 AWG	7/0.127			
DF3-2428SCC	543-0047-3	1007	1.5	24 AWG	11/0.16	100/pack		
			1.3	26 AWG	7/0.16			
			1.2	28 AWG	7/0.127			

Note1 : Crimping contact Extraction Tool DF-C-PO(A)
Note2 : Applicable wire with tin plated solid soft conductor.
Note3 : When using other than the recommended wire, contact your nearest Hirose representative.

■Crimping Contact for In-line Plug

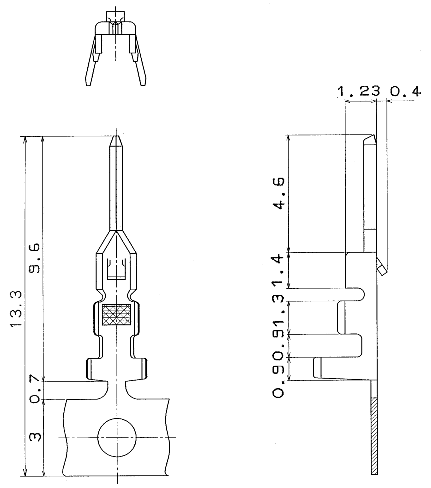

Part No.	HRS No.	Applicable Cable				Packaging	Finish	RoHS
		ULstyle	Jacket Diameter	Wire Size	Stranded Wire Conductor			
DF3-EP2428PCF	543-0062-7	1007 1061	0.75 to 1.5	24 AWG	11/0.16	10,000/reel	Tin plated	YES
				26 AWG	7/0.16			
				28 AWG	7/0.127			
DF3-EP2428PC	543-0063-0	1007	1.5	24 AWG	11/0.16	100/pack		
			1.3	26 AWG	7/0.16			
			1.2	28 AWG	7/0.127			
DF3-EP2428PCFA	543-0149-3	1007 1061	0.75 to 1.5	24 AWG	11/0.16	10,000/reel	Gold plated	
				26 AWG	7/0.16			
				28 AWG	7/0.127			
DF3-EP2428PCA	543-0150-2	1007	1.5	24 AWG	11/0.16	100/pack		
			1.3	26 AWG	7/0.16			
			1.2	28 AWG	7/0.127			

Note1 : Crimping contact Extraction Tool DF-C-PO(B)
Note2 : Applicable wire with tin plated solid soft conductor.
Note3 : When using other than the recommended wire, contact your nearest Hirose representative.

◆Applicable Crimping Tool

Type	Part No.	HRS No.	Applicable Contact
Applicator	AP105-DF3-2428S	901-4503-3	DF3-2428SCF/SCFC
	AP105-DF3-22S	901-4549-4	DF3-22SCF/SCFC
	AP105-DF3-EP2428P	901-4508-7	DF3-EP2428PCF/PCFA
Press Unit	CM-105C	901-0001-0	————————
Hand Crimping Tool	DF3-TA2428HC	550-0164-5	DF3-2428SC/SCC
	DF3EP-TA2428HC	550-0184-2	DF3-EP2428PC/PCA
	DF3-TA22HC	550-0257-4	DF3-22SC/SCFC
Extraction Tool	DF-C-PO (A)	550-0170-8	DF3-****SC(F)(C)
			DF3-EP****PC(F)(A)

Note1 : If any trouble has occurred due to tools other than designated tool, Hirose bears no respoisibility for any trouble.
Note2 : Please follow the "Crimping quality standards" and "Table of crimping condition" when crimping contacts.

Nov.1.2017 Copyright 2017 HIROSE ELECTRIC CO., LTD. All Rights Reserved.

■ Crimped contacts with cable assemblies

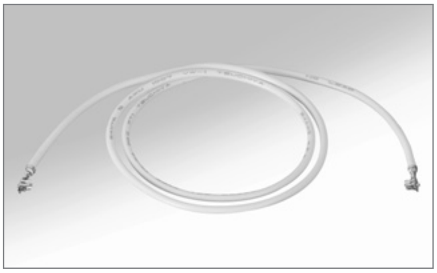

■Product Number Structure

Refer to the chart below when determining the product specifications from the product number.
Please select from the product numbers listed in this catalog when placing orders.

DF3-2428SF 1007 - 24 A 9 - 300

❶ ❷ ❸ ❹ ❺ ❻

❶ Part No.<br>DF3-2428SF : DF3-2428SCF<br>DF3-2428SFC : DF3-2428SCFC<br>DF3-2428PF : DF3-EP2428PCF<br>DF3-2428PFA : DF3-EP2428PCFA	❸ Applicable wire size<br>24 : 24 AWG	❺ Wire color<br>9 : White<br>0 : Black
❷ Wire Type<br>1007 : UL1007	❹ Crimped condition / Terminal treatment<br>A : Both-end crimped<br>B : Single-end crimped / Without terminal treatment<br>S : Single-end crimped / With terminal treatment<br>Terminal treatment : Stripped jacket	❻ Wire length<br>300 : 300mm

Part No.	HRS No.	Packaging	Finish
DF3-2428SF1007-24A9-300	543-9001-0 00	100pcs/pack	Tin plated
DF3-2428SFC1007-24A9-300	Under development	100pcs/pack	Gold plated
DF3-2428PF1007-24A9-300	Under development	100pcs/pack	Tin plated
DF3-2428PFA1007-24A9-300	Under development	100pcs/pack	Gold plated

Note1 : Bag packaging (100pcs/pack). Order by number of packs.
Note2 : Please contact our sales representative when using the other specifications.

Nov.1.2017 Copyright 2017 HIROSE ELECTRIC CO., LTD. All Rights Reserved.

Nov.1.2017 Copyright 2017 HIROSE ELECTRIC CO., LTD. All Rights Reserved.

◆Embossed Carrier Tape Dimensions

●Straight Pin Header

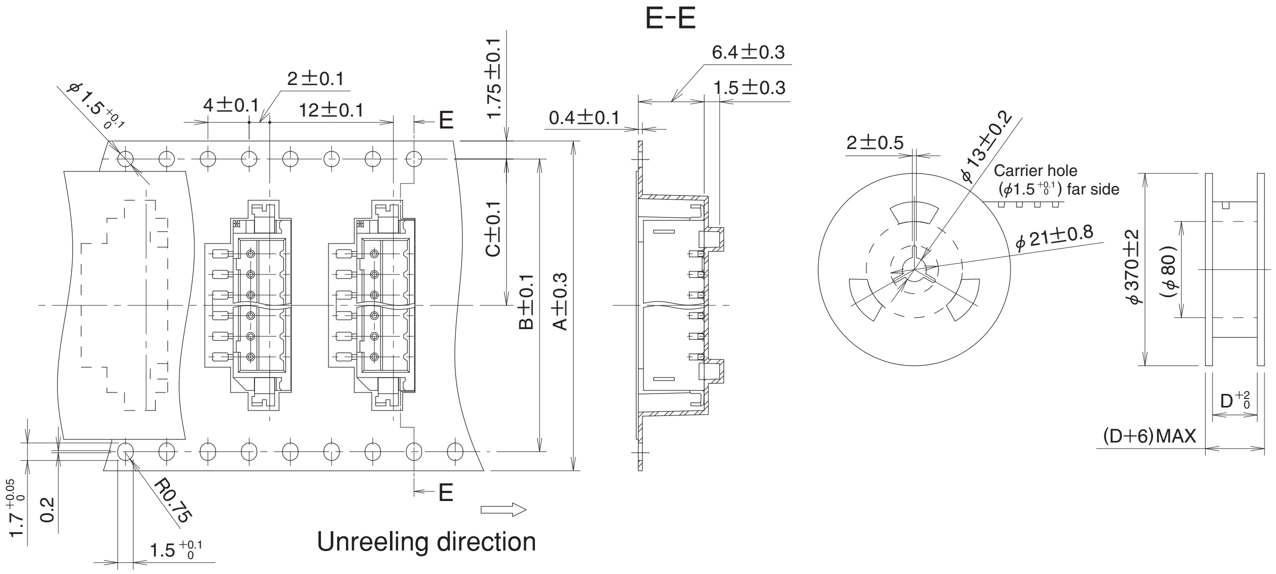

Unit : mm

Product Type / Size	No. of Contacts	A	B	C	D
DF3EA-✱P-2V(✱✱)	2 to 4	24.0	—	11.5	24.4
	5 to 7	32.0	28.4	14.2	32.4
	8 to 10	44.0	40.4	20.2	44.4
	11 to 15	56.0	52.4	26.2	56.4

●Right Angle Pin Header

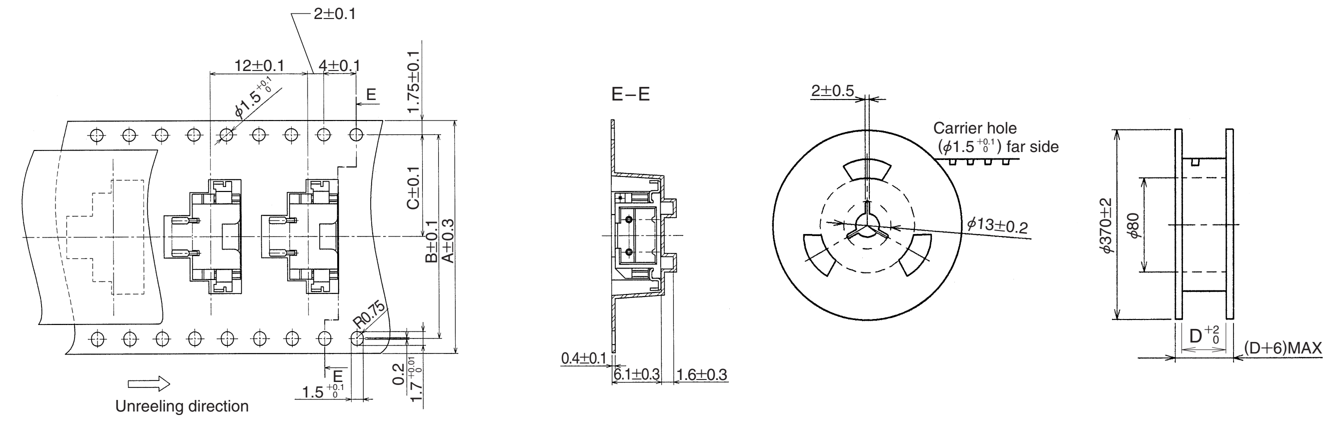

Unit : mm

Product Type / Size	No. of Contacts	A	B	C	D
DF3EA-✱P-2H(✱✱)	2 to 4	24.0	—	11.5	24.4
	5 to 7	32.0	28.4	14.2	32.4
	8 to 10	44.0	40.4	20.2	44.4
	11 to 15	56.0	52.4	26.2	56.4

◆Precautions

1. Recommended Temperature Profile (SMT)	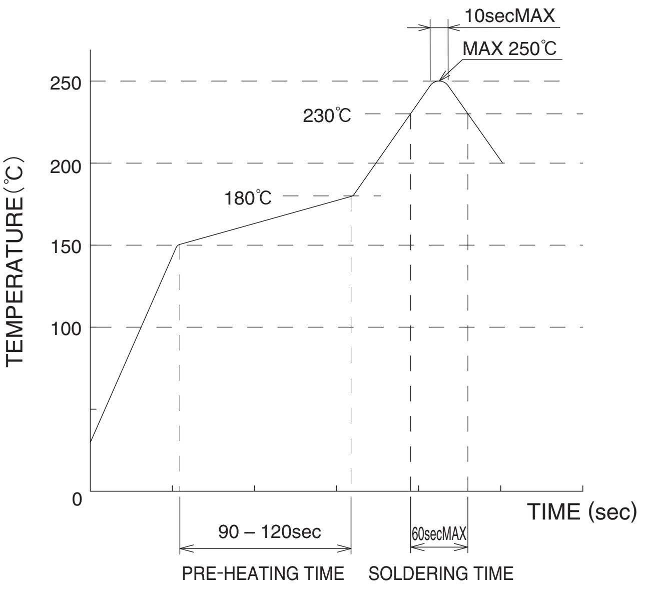<br>[Applicable Conditions]<br>1.Peak temperature : a maximum of 250℃ for no more than 10 seconds<br>2.Heating unit : no less than 230℃ for no more than 60 seconds<br>3.Preheating unit : 150 to 180℃ for 90 to 120 seconds<br>4.Number of times : no more than 2 times<br>∗ Measurement is conducted at the contact lead part<br>As it may change depending on the conditions such as solder paste types, manufacturers, PCB size, and other soldering materials, please fully check the mounting conditions before use.<br>(Note 1) This temperature profile is a recommended value.
2. Recommended Manual Soldering Condition (SMT)	Soldering iron temperature : 290±10℃, Soldering time: Within 3 seconds
3. Recommended Screen Thickness (SMT)	0.15 to 0.2mm
4. Board Warpage (SMT)	Based on both connector edges, 0.03mm max. in the connector center portion
5. Recommended Soldering Condition (DIP)	■Flow Condition with Automatic Soldering Tool. Soldering temperature : 250±5℃, Soldering time : Within 10 seconds<br>■Manual Soldering Condition. Soldering iron temperature : 290±10℃, Soldering time : Within 2 seconds
6. Cleaning Condition	Refer to the "Wire-to-Board Connector Use Hand book".<br>IPA cleaning is allowed. (Cleaning is not recommended because cleaning may change the push/pull feeling etc. Please contact us when you use other cleaning agents.)
7. Connection Condition	Refer to the "Wire-to-Board Connector Use Hand book".
8. Precations	Refer to the "Wire-to-Board Connector Use Hand book".

Nov.1.2017 Copyright 2017 HIROSE ELECTRIC CO., LTD. All Rights Reserved.
