# Supplementary material for: Imaging brain activity during complex social behaviors in Drosophila with Flyception2
Source: Nat Commun. 2020 Jan 30;11:623. doi: 10.1038/s41467-020-14487-7 (PMC6992788; doi:10.1038/s41467-020-14487-7)
Supplement: Supplementary file 20 — Supplementary Software 1 [file 41467_2020_14487_MOESM20_ESM.zip › Supplementary Software 1/Altium/ArduinoLensControlAltium.zip!/Altium/20171121 Arduino Shield Lens Control/DF3EA-8P-2H(50)/DF3_05430335050_3dstep.zip!/Read me.pdf]

3D-model has been simplified, please use the latest 2D-DWG for your design.

3D-model may not be distributed, modified, posted or otherwise used without the express written permission of Hirose Electric.

You may download 3D-model only for your personal, non-commercial use.

Hirose Electric may make improvements and changes to this data at any time without notice.

Hirose Electric does not guarantee software compatibility.

この３次元モデルデータは、形状を簡略化しております。製品の採用に当っては、最新の納入仕様書／参考図にてご確認下さい。

お客様は、この３次元モデルデータを複製・改変・譲渡・転用または営利を目的とする使用等を行うことはできません。

３次元モデルデータは、予告なく変更または削除させていただくことがあります。

ご利用にあたり、種々のエラーまたはその他の障害が起こる可能性がありますが、
弊社はこのような障害によるいかなる損害に対し、一切の責任を負いません。
